# Supplementary material for: Discovery of novel representatives of bilaterian neuropeptide families and reconstruction of neuropeptide precursor evolution in ophiuroid echinoderms
Source: Open Biol. 2017 Sep 6;7(9):170129. doi: 10.1098/rsob.170129 (PMC5627052; doi:10.1098/rsob.170129)
Supplement: Figure S2 (Ophionotus repertoire) [file rsob170129supp2.pdf]

Figure S2: *Ophionotus victoriae* neuropeptide precursor repertoire

Bursicon alpha (partial)
IRLSISMADCRPRNVYPFGCRGQCASYTRVSPANFLEIDRQCKCCQVGEQVDLQVRLDCPKLKPPVGMVTVKSAKNC
SCRPC

Bursicon beta
MEAYRKSMASHNLPVFTSVILTWMICSYFPAICSANRNRCTMSTSLVTIQDRRIIDGDTRATMYCSATVEVNQCEGL
CKSWELPSATSPNGFDKVLWKEIGQRSKGRTRGINTLGRNRKSCKCCKERYLESRTVLLTECRDLPRADGGVLLPGY
VYPLEITEPASCRCETCTI

Calcitonin (long transcript variant)
MKSGVAITLAVCSALYYAVTLVSGLEKRSIEEESTLPLTGDDLRDVANKLDIFNAVVTRIENTFPEQFKRSGNGGCA
GFTGCAQLAAGQNALRNFMHSNRASLFTGASGPGKRKRSIVDSSTLPLTGDDLRLLAEEVDLVNAVISEMAAKYPEQ
FKRNGNGGCAGFTGCAQLAAGQSALQAMIHSGRASLFGSGGPGRRRRSTDEVLH

CCHamide-like 1 (Arnp25-like)
MFSIYSFSGLARLLLLICLLIALTHPVYSTNHCKGRLPKFCFLHPGKRNSQQTTAVTDDREGQIQLDNMQLSNSDSS
VRTMKAILALLLGPVNQEERYPEVEDDDSLSRSQKVTILTRLLESRSNSY

CCHamide-like 2
MSTTSVSITLLLITLMVALLPSAYSRGICSDPLACGAAFGKRTSNIQIPHNNFDDLQIPLETNRGLTFSTDDDDDRP
VNMSFLQSLFKLLPQDRQQRIQQGLRQRMEDELFTARRR

Cholecystokinin 1
MTGDVPPLLLAIVCLFLTTTPTVSSLPSKSGNIKTSRDLTQTDIERLILNTIENVQAQAINRQNNVGTHDDLGEPLW
KPENTWKTPTLPDLIQDWKDSVKERHDSLADTGLLQDIDVEVDRDEDDIADKRSKDYGWGMAFGKRGSRTQNDRQTH
KIESRNKDYGWGMAFGKRNEYGWGHMFGKRNLEQVDYDDFVA

Cholecystokinin 2
MDFKIPLLLVTLSYACVIFMTSSTDGIPLGDVFEQEVAEELGLYKPRPDRRQDRLLASLQNSGKSLDYGFGMGFGKR
SPSLDRRATWRNTRQRLVELSGNN

Corazonin
MRNCSTIGAALLLAVILGSILGDAHNTFSFKGSNRWNAGKRAQMDNTHGLTDRLTDTSNNFVPKESAATTLRQLLVD
LRDYCDSLVRVLDESRIESERK

Corticotropin-releasing hormone 1
MELPTSHHKLSAMCLVLCLLQCVFTTPVYQQVRQDLLEQLRQRERRYASNTHYTLYPEELEDPFGFKTDRLKRTGSP
IALNPGLVVLDILRSTIDNDRRRQQMSEAAAMNSELFTRVGKR

Corticotropin-releasing hormone 2
MKDYLVMLFASLTVILCLARSGYGTPNDFMDYKRDPRTVIRSATLDRQTLKQMLTGLARERAYLVDPDHYQQQKRQM
NLDLFTTFSVLREAFESAKNERDRASALAANGRLFAAGGRR

Corticotropin-releasing hormone 3 (partial)
GLARERAYLVAPSYYQKRQMTVDPFTTMQILRDLHQTAEKERQRQKAIDINGRLFAAGGRR

Corticotropin-releasing hormone 4 (partial)
SGYGTPNAFMDYKRDPHTVSKTTTLDREALSQMLSGLARDRAYIVDPEHYQPEKRDNFEFGLFTSLDILRDAFQSAK
SERERADALAANEDLLAAAGRR

Eclosion hormone 1 (Spnp11-like)
MKTVMVSCLVLLLIGESFGAALLDIDEVDDTNAAFALNRLVQRRSDGNDVLMERERRRKSCLVECVTCSRYTLLPTT
DCYEGCNKPTKSKLALNTWTACKGMLGGQ

Eclosion hormone 2 (Spnp15-like)
MNYTSVLVFVLWGVVMLFTLVRAIPLLEAENNGLDRTAFNAETANIFNMARRTSLDLPGRGDFERQQRAKHVQLKIC
TLRCVSCNMEITGYQFDHCLGGCRLGRKNDNNCRRYITK

Glycoprotein hormone alpha-2.2

MGEDKDHRCTAEQTTLRWKLPSFWGIRIYALFLLCFYLVFSCILCPPVSSADNSALSWKKPGCHLVGYTKEVKIPGC
YTATVAMNACRGFCMTYSFPSDKDTLDRSNGDKYLTSHGSCCTIKSTHDVHVTLQCENDHQYRDTFKSAAKCDCAIC
DQSD

Glycoprotein hormone beta-5.1

MATSQQYYTQWNVSALCFLVLVAFIEAIDPSTTTGCFIHTAMKHMAEKDGCRSHEFFVRGCWGRCDTSEVPQLMPPY
VKAKHPVCTYATYDVMTVVLPDCDPEVDPTYTYLSALSCGCQVMLARKTEYSYRPDFYLQTAEQ

Glycoprotein hormone beta-5.2

MTMPQQILLEWRKTTVLCSVFCIILAACFVCVSSGRSGGSRVASLDCNVRWFLQHEAKKLGCRTQTIGLNACFGRCD
TYQVPILEPPYKTSNHDMCSYGAMEYKSVELDDCDVGVNRTYVYVNALSCRCRQCSTYDTHCLGVH

Gonadotropin-releasing hormone (partial)

MREPTKRSVLVVWALLLIFSEIHSSHAQLHSRMRWEPGGRKRSSTQATLDNRRQWTQNQRKRSYSLTQDTATMTLIH
NIAKSLARTISRKALLSRQQSLSVFAPEWSSGSIPYENERQIWQW

Insulin-like peptide (partial)

VTPLGRLCGKPLADMVAYVCEGRGYYVGETDSPMLSERQAKAFVSGRKRTGKIVTECCDNHCNIQIIESYCAPRHVH
PGQNQGSIVLSRSEQELVNEQNNVVDAPPPPPPQPEEEVVQDGTVPEITQEIIDTLSDQLDLEDSIESETNLLADAM
PALMLADEVRETSEQRLNADHEGEDIPKEKGENAVTDMTDLLETQTELVDEEQSEHRDRPHLNKTKGNTRTNRPSSR
ERNSRRKNSREKKKNKAKTTTSRERKAKRRNKSKNKKNRRRKSKHEQDDYTPPTDQLITGSEVVHPKLHIINAEPPS
R

Kisspeptin

MRSWCMLRLLPLALICAVGLPVPCSGDRSAKKDIPLLREEELQNSPDYGLESANIFIDVIERILDRLESSAAPPYDN
DINISPETSWPPLENDFLSREHIYLPKPSTNGLILSLTSFQKRQGSTACMNVMCRMIRGRPRVNANAGSRALPFGKR
ADNVRNSADDGPRSVRGRRRGRGRPRTRGSPNGHPQQHKLPFGKRHLL

Luqin

MQSKVTACVCVMAVLLVVQVVTAQGFNRDGPAKFMRWGKRGDSTDSALGSSLELPLFGNTKIICRYAGEAGLYNCAG
VSGAFE

Melanin-concentration hormone

MQVSAIILTWLAAVCLMSCYTVANAKPYHETDAQKLLQKLQELGILKEPIYNDVTPYANDEEAFAKRSSSPNDIRRY
SVCYDPIKLRWRRCRGMGSKTRHYTDIQ

Neuropeptide-F/Y (partial)

ALDAILSGQYRSHLRYGKRFNPTLLKDNSITNSAAMS

NG peptide / Neuropeptide-S

MAVGIRNVILHLILVLYVARTILGEIDTYKVRRYTDGSPVNSNHWTQSDNINELRKEIFASLPADLPGIIHKSKTHD
SRSGRHSDELSNLEQLGNVAGKDGANDAKDKLALYNFMSKQATRNNLGDELDKRNGFFFGKRNGFFYGKRDVEAVNN
EDSCVRCGPENRGQCVMFGTCCSPQFGCYLMTKESEACMSNHIGTGCRNLDMAPQCGSTGVCVAKGVCCSPQDGACH
IDVTSCLSDNTTIDKSL

Nucleobindin / Nesfatin (partial)

MAKWHYLLGILSLLTVLCNALPVVPKEPVEEEKPEEELLEDEDTGLEYDRYLRQVIKVLEKDPDMRKRMEEMSLEDL
KEGNFARELGFLSSNIRSKLDELKRMEVQRLRTVAKQRMEAATGTGLRRMDQKALEGMVGHVDPATMDKFTDTDFEK
LIKKAAADLDEADQERRKEFKRYEMEKELKRRQDLKTLDQAAQVKAEAEFNERRDRLKSHPNIKHPGSKAQMRDVWE
ETDKLDPEDFNPKTFFALHDTNNDKKLDMFELEALFIKEVEKIYQDKEADPREKFEEISRMREHVLREIDLDGDKMV
SREEFLKAADQTQFENDDGWKDINQEEQFTEDELQEYNRMVNERLER

Orexin 1

MKLLGCLLTTLILMIALIAIPTTADRACCRLTTGCQLRTDCLCVAKEVMCRDPSVGLLNMGKRFKTLETNDEQDEES
KRAQRNRAKRAYGKELLDTLLLS

Orexin 2

MPQTGPFLSLIVFTILLYLTGLLAQKQSCCRVKGCSIPPDCDCPLKQELCKDVTKGILSMGKRTRSYEENVYRQLDQ
NKDRQHQGTRTSSKVMNTILKLLQSQEQEDEPQDWKPMLSRNLWENEDFNEDLYDQQPNFYAD

Pedal peptide 1

MMKFLWSVLALAVFVCIANAVAYGTDEILEAQDDANTLMVDEAAADKRAFQNFMDPLSAGFASKRHFSNFVDPLNAG
FAGKRFSHFMDPLSSGFNNRPYKRRFSQFMDPLMSGFNGGSQKKRDFNTFMDPLSSGFNKRFQNFMDPLQSGFHVKR
DEE

Pedal peptide 2 (partial)

FNNMMDPLNAGFTGKRGFNNMMDPLNSGFSGKRGFNNMMDPLNSGYSYGKRGFNRMMDPLNAGFTG

Pedal peptide 3 (partial)

MILLRWLSGKSPSSALILVYTLTLFTSHLTATVAFDKEDSIEDQDEDGSSSSITEVDFLKDEHILVKDLLPTVEDLL
RTELARLNLKINNADESSAYRTNTDTGSAFLNNLLTSIRGSGSGEDVEDVLSSLEEKDKRGRRRFHTFVDPLSSGYW
GKNGDPEVDSKRGRVTFNGDIVDPLAAGYRDGEEEPDKRGHVNFNGPLDQLKSGWKRGHVSFNGPVDRLHSGWKRDD
ENVDVDADEDLARWNKRGGVNFNGPIDALDSGWDKRGHVNFNGPIDRLHSGWDKRGHVNFNGPMDNLHSGWD

Pigment-dispersing factor

MHITLMFTSAIVAVLIGLAASSDLLEDKRIADNDFAQMRSIADRKNEAIAFRNLLSQILKEQGKRDVQKRLSQNDFS
QLRSNVLDQELTKQLIARFLSEAGRR

Relaxin-like peptide

MTTLHKIVAITLSALLILGATSIAEGKPTYCGSDFIRVVYETCASLIKRTSPVWQRLYTAARVRRFADPEFWEDVYN
NDDVAMDKRQDQGMAQYCCRHGCSDQEISRVC

SALMFamide (L-type)

MRLQPLLVFCICALVPFAATGTIPRRRSGFEGANYNYDVLVKDTTQLEDENKEIDERRSGRRNPSLNSGLIFGKRFE
EAAEDFLNDDESRQINLVSRGRSRLPFHSGLMQGKRNPLQDNLSVKRSRPQFHTGFMMGKRFTPEADDFDLEEFKRK
AGQRLRFSDGMLFGK

SALMFamide (F-type)

MARVRNILILLAAICCHATLSHAEDEDTEELNHEQLVEFANKIMGQMKLLEYELGIQEHNDGQLDMVKSLSKRQAVR
PGGGAPMNVPVKMSGFSFGKRDAQLVRRSAGATPSKLAGFAFGKRGQPVKRSSDNEAEEEQEKRGAMDAFAFGKRPS
GDPMSAFSFGKRRNPMNSLSALAFGKRAGMDPNSLNAFNFGKRRDPLSAFSFGKRGMDSLSAFNFGKRGRDHLSAFS
FGKRGRNPMNGLSAFDFGKRGGMDAFAFGKREQEYNEEGAFDDEAEKRGYENGLSGYAFGKRDTTDDQLNHNDDTLR
TD

Somatostatin 1

MKYLNLTTFAALLLAGAVVLVQSAPRRNSLNTWPFEMEEENTAADQFQQSPLLNKLLERIYSQLEDRIGNTNNEYVN
SQSRPRLDLEQLTFAAGANDIDEAAIPVRPVKRGKCVGRFVPYMMNC

Somatostatin 2 (Spnp16-like)

MNSAISCLLLIAIVAIFTIGLSLGAGATITRDEITDSDRDENNVANVAARMVMHLWNQNLNSRLNNKRGGDYGEDDT
VMVDDPVKRRRPGCVYDIWKGRGLSRCT

Tachykinin

MASVKSWTRKMQAVALILCGVFFCCGQGQLTPDNSGVLNELTEFEDDTTYPDLDPDWIDPEDAAFLAPILTPDLTWE
RVPLQLIKRRKNNVFSAGLFGKRNGWSQGQQSGLFGKRNWIEEYVNGARDVDAATVDEAVDTMEDYGQTAGHYAKRQ
RWNQNQQPGLFGKRDNAMNTAARRTYKEMLKTLQDKVESSLDKRSSGTNNMGRVRTKSSGQHVFRSGGLFGKRSAEA
PGMQRALWPENEQRRK

Thyrotropin-releasing hormone 1

MQEFSFYRCSLLAPLFLLLALGTVGFADPGDFGLDGAANDITPLREDWSGNDLEDGAMEEMENEDKRQFSPGKRLLL
LGKRQWVGGMPEMVDMPQSRQFSAGKRQFSAGKRQFSAGKRQFSAGKRQWIGGEAVDDEEAKRQFSAGKRQFSAGKR
QFSAGKRQFSAGKRQFSAGKRNWGEDLTPEEDLTPEELLAMGLIPAPETRQFSAGKRQFAAGKRQFAAGKRQWVGGE
EEQEFNPDDFMDMETRQFSAGKRQFSAGKRQFSAGKRQWVGGDLIPDMETRQFSAGKRQFAAGKRQFAAGKRQFSAG
KRQFSAGKRDTTNVLEEFQDEDPAA

Thyrotropin-releasing hormone 2

MLSNTPVSAAILILSVAVCWTPGASATDNALTESENQYLFDRLLYNLRSSENRQKSVDDLIRNYFPGDQTSTGNKNV
LLQVLARLGNVPFNFGGPNEDPSSTVSLNQWPSAVNDDNRQPNIVHSQLVEDDMSKRWGSGPGPRGKRLAIDLRERR
RDGPGPRGKRQGPRGKKQGPRGKKEQDGDEDYCLETIANTRYLCHCSATIDPAKCQVVGDK

Vasopressin / Oxytocin

MACLCWALLLVLWVQGCLSCLVSDCPEGGKRSGYSSLRQCHSCGPDGSGQCVSTSICCGNSFGCYMGTPETIICEKE
NELSSPCEPKGDSCLSVSEGKCVSNSICCNQRSCAEDLACVIIGLPMRTSVEDLSTSGQDYKKSIQDRLKSKLLEVL
LRQP

Ovnp18 (partial)

MQSSNIAVVATLFIFAAVFAQAAYSLSFEAPQRSKRLFWVDKKAETSQPDKRLFWVDKKTPEKRLFWVDK
KTPEKRLFWVDKKSDGEHTNVDALAHCFVKVFSNYMDHVAECKTAGVDNLQTCMEAVKEKSSMANANCLS
DPEGGL

Ovnp26

MLGVKTTLAYAAIVLALVVSTGTSEDIEPETDEFELADDADDVIDFKDLEPDYQELLINLQRARAASAQD
LDNKRMSSGWKRAQGAAGWKRAQGAAGWKRTQGASGWKRAQGAAGWKRAQSASGWKRDSPVSMETRGDGN
WRNSNVKSASAGWKRTSRSANKQQRIEDVN

Ovnp27

MRLITCTTLLCVLLALIDTRQSYCEAGRIPVRYQTGQIFGKRQMTDEDVLRALDGPYGRNLLDLMKLSVYRLNQLEG
AQEVESK
