# Supplementary material for: Discovery of novel representatives of bilaterian neuropeptide families and reconstruction of neuropeptide precursor evolution in ophiuroid echinoderms
Source: Open Biol. 2017 Sep 6;7(9):170129. doi: 10.1098/rsob.170129 (PMC5627052; doi:10.1098/rsob.170129)
Supplement: Figure S3 (Amphiura repertoire) [file rsob170129supp3.pdf]

Figure S3: *Amphiura filiformis* neuropeptide precursor repertoire

AN peptide (partial)
METRIVLICISFVVFLAHISGAPVEEQEIDEDKRGNMHLQGGGGRGRNRRPGKRDGEDADIAELVDEATEEEKRGNAYL
QGGGRGRGRGNGKRDDEELQELLMEEEDKRGNRFLQGGGAGRGRGGRRPGKREDGEFTDDLLDEDKRGNRYLQGSGPGR
GRGRPGKRDDEGEEEFELLDEEDKRANRYLQGGGKGRGR

Bursicon beta (partial)
MNTAACLLQSCSSLLLVGLIAYLVPVLHASRNNRCTLSTSAVSIQDQVVDGDTQTTASCRGTVELNMCEGRCRSWEFPS
ATTSDGF

Calcitonin (short transcript variant)
MRTSLAITIAVCSALYYAVTLVSGLEKRSYIDNEVSLPLTGDDLRILADKVDLYNALIGHIENTFPEQFKRGTEKGCAG
FSGCAQLAAGQSALQAMIHGNRASLFGSGGPGKRKRSTDEA

Calcitonin (long transcript variant)
MRTSLAITIAVCSALYYAVTLVSGLEKRSYIDNEVSLPLTGDDLRILADKVDLYNALIGHIENTFPEQIKRGGGCRGFS
ACAQLAIGQDAFRNMMHNNRASIFTSARGPGRRKRSIDEQQVLPMSGDDFRLLAEEVELYNEILGQIASTHSKQFKRGT
EKGCAGFSGCAQLAAGQSALQAMIHGNRASLFGSGGPGKRKRSTDEA

CCHamide-like 1 (Arnp25-like)
MHNNSRQTGLVRLLLLICLLIALVHPVYSDRHCQNMLPKFCFVHPGKRNSQGTKAIDRNQIGEAGLEQGTMQLSNSDSS
PSLSKMEAIIALLMGPLNNDEETQTEDYQEEDTNSLTRTQQDKILTRLLLLSRKNTP

CCHamide-like 2 (partial)
MMTIRGISITWLLMVLVAALLPSAYTRGICNDPRQCGAAFGKRTGDIQIPQSFEDIQIPLQPNRDFELQSGSDADNLSF
IQSLFKLLPKERQQRIQQDLRKTLADELFSSRR

Cholecystokinin 1
MTGDVIPLLLAIACLFLTPAISLPSKTGTARDISHTDIERLILNTIETVEAQAINRKNGGTNEDEVGGTPWKAENTWKT
PTLPDLIKDWKESVKERQESLAENDILRDIPDADAALNDKRSRDYGWGMAFGKRGSRTQNDRHKLESRNKEYGWGTFFG
KRNEYGWGHMFGKRDAEEVDYDDFVA

Cholecystokinin 2
MDFKIPLMLVTLSYACVIFMTSDALPLGDAFEQEMAEELGIYKPLPDRRQDRLLASLNSGKSLDYGFGMGFGKRSSPLS
RSGLSSWRDARHRLMELNAEN

Corazonin
MRNCSVVASVLIAVIFGSILCDAHNTFSFKGSNRWSAGKRAQMDTNGLTDRLTQTTNSFVPKDSSSTTLRQLLVDLRDY
CDSLIRVLDESRIESERK

Corticotropin-releasing hormone 1
MEIPLQQSKLIAMFLALCLVQSVFTSPVSPSVRQELLEFLRSKERKYSQQLDTDVVEADDTQNTLHMDTDRLIKRNNGS
PLAFSPGLVMLDILRAEMSNNGRRQQMSELAAQNSELFTRVGRR

Corticotropin-releasing hormone 2
MKVQIKVVLVGTLTALCLVHSDQRIPEEYLDYVKRDNNSEIKSAKFDRDAISDFLMRIAKGNIRMRLSDPEQVTKRQFG
QTTNFNAGLSLVASLIDRALQEIEMRAYNTQVLENADLMRQGGKR

Corticotropin-releasing hormone 3
MKDHIKVTIFATLTVVLLCLVRSGHGLPGEYLDYIKRDSASKPEIRSAKFDRKAIKDFMNGMARERSYYYDPEQVAKRQ
FGQMTNFNPAIQSLAVALDRLLDEMDFNRRVTENKNRMQAGGRR

Corticotropin-releasing hormone 4 (partial)
MKYHIKVTIFATLTVVLLCLVRSGHGLPAEYQEYTKRDPRQEIRSAKLNLGDFIRGMPRGRVYFNGNEPGQMAKRQFAQ
MQSLNSGLGILQAKADDLLHEIDRNRRLRENAEHFRA

Eclosion hormone 1.1 (Spnp11-like)
MKTVMVSCLVLLLLGDTFAAALLDDADENDAAAFALNHLVQQRSDGQQELMERERRRKNCLVECVTCSRYTLLPTTDCY
EGCHKPTKSKLALNTWTACKGMLGSK

Eclosion hormone 1.2 (Spnp11-like)
MKSLIYLCALALLIEVVMPAPGVFDLQAFEDNDNTADLDALDLNDMARDRRSKTCLTECFACSRMVSRINPSQCVSGCQ
DGGKSIKGTAAKTWTICFMALQRRK

Eclosion hormone 2.1 (Spnp15-like)
MDKTSVILVLSGLVLILAIVGAIPPLLGDEQVDQDTALNDEMADLFSMARRASGYDFSERAKRAKFEKCTMNCVSCNKE
IKGYKFLTCLTGCRSSSKGDSNCLRYLTK

Eclosion hormone 2.2 (Spnp15-like)
MDKPTVVILVLSGLVMLLSLVSAIPLLAAENNAAETDEGVYNDDRAHYFNLARRTSPLELPGSEDFERQQRAKHMQLKI
CTLKCVSCNMEIKGYQFDHCLGGCRLGRMNDNSCLRYLTK

Glycoprotein hormone alpha-2.1
MMRSEVLNALIASLLLFGSLLGSTAQHAWERPGCHLVGYTKEVRVPGCHMEEVPMNACRGFCLSYSYPSSLARLLESEG
SQILTTTGNCCSIQETHDVNVWLRCENNEERLVTYRSAAACECSLCEV

Glycoprotein hormone alpha-2.2 (partial)
MGEAQHTCIGAIWWRIPSYGGIRLYALFLLCFYLTICSILCPPVASQRSGGPNWKKPGCHLVGYKKEVRIPGCHSASVA
MNACRGFCMTYSFPSDTDTLVRSQGYKLVTSHGSCCTIKSTHDVHVTLQCENNHQYRDTFKSAAECECAI

Glycoprotein hormone beta-5.1 (partial)
VFALLFLIYIIGVTSAIDPSTTTGCFVHTAMKHRAEKEGCRPMEFFVRGCWGRCDTNEVPELVPPFVKPEHPVCTFATY
KVTTVELPDCDPGVEPSYSYLSALTCSCRTMSASQTEYSYRPDFYL

Glycoprotein hormone beta-5.2 (partial)
MRMPQQIVFVEWRRTTVLCSVFCVLIATCIFSVTSAARSGGSGGGRPSALNCNVRWFLQHNAEKEGCRTQTIGTHACF
GRCDTYQVPILEPPYKTSNHDMCSYGAMELKSIELDDCDVGVNRTYFYVNALSC

Gonadotropin-releasing hormone
MREATQRSILVVWALLLIVSELHSSHAQIHGRIGWKPGGKKRSFSPSSLDNRRQWTQNQRKRSYSLSQDTPTMTLIHNI
AKSLA

Insulin-like peptide
MNILAWLLYIKALVCLLQPVSSMAMWGRLCGKPLADMVALVCDGRYYTGQTDSPLLSERQAKSFTSNGGRKRTGKIVTE
CCDKHCNLQIIESYCAPLPEGQTRTQLRHWFLHEKEKESANEENNTVEELPPPPPPRSEERDEEQVPDVSQDLTFSDHD
WEDSIESETLNEAIVMLADAPLTEQNTEEELLHKKDIKSGTNTTMATQDEAREEDVILSESSDRTKGKGNRTHRPTHRP
SSERKSRRRNSKEKKRNKSREKKNREGKKKNKSKNKKNRRRKNRHGEDDFTQPTDELITDIEVMHPQARVVSRSEPVHA
VDDPTDPGSRSERSFLTTITAKLLDVIGLQQNSNHDRRR

Kisspeptin (partial)
MRSCSMLRRILVAFIFALPILAIAENRHLDNDIPSLIVTEEDQDNSSDFGLQSANILLDVIERILDRLESSAVPPSQPD
NNDILQSVPETSWPPLDKDFLPRDLIYLPKSSSSSNALALGIASYGRSRQRGFAPCTKGLCRVIRGRKSGNANAGARAL
PFGKRNSDGPDTATSSVRGRRRGRGRPRSRGSLPQQNT

Luqin
MSKVTACICVMVVFLVIQVTTAQGFSRDGPAKFMRWGRKGGDDSVDTAALQLPLFGDSNIVCRSTGESNLYRCDVQ

Melanin-concentrating hormone
MQVHVVILAWLATVCLMSCYHTACATNLYDTDRQQILDSLESLGILQEPTNDDTLLYTADDAYSKRASHDTTRRFSVCY
DPIKFKWRRCRQGMERARNQKQ

Neuropeptide-F/Y 1
MKQRIAMDTKILLIVAAVMCCLISDAHGTNVRRATTGDKALDAILSGQYRHHLRYGKRFDPSLMKDNSQTDMGAFSALW
NEAAHNPELKQKLTSYVKQMWQEASRDAQ

Neuropeptide-F/Y 2
MDLKLFLLVGLCCVVRAQSNMRDKAMQAYESGQYRKPHARFGRNFEMPAVDVAEAEPNKELNEILNTISDEEGELLGPD
MSDKWHLPLDKQLLKYFWSRKSRST

NG peptide / Neuropeptide-S

MALGIRFYTILNLILVLLAARTIFGEVNTQDSSHKVRRSPVGSSGNSIQWTKDDNINKLRKEIIASLPADIPAILLNPQ
TQENKEDTPQDLTALEQVERVATGQTIDTDARDNLYNFLSRQSARNNYQAGLESKRNGFFFGKRNGFFFGKRDTEAVKS
DLCVSCGPENIGQCVMFGTCCSPQFGCYFMTQEATACTKHHIDNACWNQDLMPSCGRRGICAADALCCSPKDGACRIDL
SCSTP

Nucleobindin / Nesfatin
MARWQYLLGIFSLLLVLCNGLPVLPKEEIEDEGLLEEEDTGLEYDRYLRQVIKVLEKDPDMRKRMEELSLEDLKEGNFA
RELGFLSTNIRSKLDELKRLEVQRLRTVARQRMEAAAGKAGAKRMDPKALENMVGHVDPATMEKFTDTDFERLIKKAAA
DLDEADKERRKEFKRYEMEKELQRRQQLKDLDETKRLKAEEEYKQRKEKIKNHPKIKHPGSKAQMQEVWEETDHLDPND
FNPKTFFALHDTNGDKKLDMFELEALFIKEVDKIYRDADEADPVELAEEVSRMREHVMREIDLDGDKMVSRDEFMKAAD
QAQFENDEGWKDINQDEQFTEEELQEYNRMVQERLERKKIRMQEEHDRFVKEQNINMPNGVQMNQQQQQVNQQQQGQDP
KLRVNQPPINNQVPVNQGQVPAQQAGQPIQQGQPPVNQAGGQAPPQQQAGGQPVQQGQRGAVPPNVAQNQQQQQQLNQQ
LNQQQQINLQQQQQQQINQQQQQLNQQQQQNINVQQGQPKPPVQAQGQPVQHVIQGGQPAQPPQGGAQGKPVQNVIQGG
QQPPAQPPQGGGQPVQNVIQGGQPPAQPPQGGVQGQPPAQPGQGANPPGQ

Orexin 1
MKFLACLLASLALLVAALAVPSRGNRACCQRTQGCNLRSDCKCLAREILCRDPSVGLLNMGKRAKETLDTTFDVDEDEA
DRQTRRRVARRSFEGKYLDLFV

Orexin 2
MQRPGQFLTLILLGILVYLTGTLAQRACCKSTKGCNIPPNCDCPLKKELCKDVSKGILSMGKRTRSYEENVYKQLEQNS
RDRQPEIRTSKIMDTILQLLHTDEQEDQHQDWKPSLRNLWKPEEFNEDLYEQEPNFYTK

Pedal Peptide 1 (partial)
EEKRGTGRFNNFVDPLASGFRPNEAEEKRGTGRFNNFVDPLATGFRPNQGEEKRGTGRFNNFVDPLATGFRTNTGEEKR
GTGKFNNFVDPLASGFRNNMKGQEKRGTERFDNFVDPLKPEGVTKEEQEKRATGQFNNFMDPLESGYRNKAKATKEQSS
NDLLLNLLTRFLREDSDTRNGV

Pedal Peptide 1 (partial)
QITMMKFLWSLLVLAVMFAVASAMAFADERELAQDDTDTDTLLEDETPDKRGFHNFMDPLSAGFANKRFNQFMDPLQSG
FSARPYKRRFNSFMDPLMAGFNPSKRFNSFMDPLNAGFSNFPNKRFQTFMDPLSAGFHVKRDDD

Pedal Peptide 3 (partial)
SPLDSLGSGWKRAHVNFNGPMDALSTGWKRGHAYFNGPLDSLSSGWHKRDVSSQESGDGEQDQAEEKRGHAYFNGPMDS
LGSGWSKRGHAYFNSPLDSLASGWKRA

Pedal Peptide 3 (partial)
LSSGWKRDSENAENLEEEKRGHVNFYGPMDALRTGWDKRDGEDDGEVEEDKRGHAMFHGPLDSLSTGWKRAHVNFHGPM
DALSSGWKRGHAYFTGPMDSLGSGWKRAHANFYGPMDALSTGWKRGHAYF

Pedal Peptide 3 (partial)
YTIKMFLLECLRGKQSPILLVFALTVFTFYVEKSVAFDKEDNNDQDEDGSSYTDEDYLKQEHKLFNNILPNVENLLKTE
LYRLNQKLTNANGATFRTEANNETPFLLDNDDETDEALDNIFQDNKGGNSPNVEGYEGDIEENKRANQFIDPLASGFRQ
DTDDPGKRAHVNFHGPMDALSSGWKRDSENEADKRAHV

Pigment-dispersing factor
MHATLLFSSAILVVLLGLAASSSDDLPDKRIADNDFAQMRSQADRDFEVVAFKNLLKEYLRTYGKRDVEKRLSQNDFSQ
LRSNLLDEELTKQLIAKFLHQAGRR

Relaxin-like peptide (partial)
MATLHKVIALTLGTLLILGATSSADSAKYCGLAFSRAVMETCARQVKRTAPLWERLYTASRVKRFSDPEFWNAVLESDS
DISMDKRQP

SALMFamide (L-type)
MRLQPRIVVFICALVPIVLAGTIPRRSGNETPTFNYDAMVKDQQMENEDRDIEERRSGRRNFLNSGLLFGKRFDETGNF
LNDDEEDEDTRHFNVEIRGRSRVPFHSALMQGKRTPLEEEGLMSRSKKRSRPVFHSGIMLGKRYPLHDVVDKRKKSRLR
WSDGMLFGK

SALMFamide (F-type)
MARVRNVVLLFATLCCYASISSGDVSGDEIQDNNQEQLEELAYKIAEILKSNYENDEDLVKSLSKRQANNRPGSGLPMN
VPVKMSGFAFGKRDGQLVRRSAGAQAKPVKLAGFAFGKRGQLVKRSSDDKLMEDEEAEKRAALDAFTYGKRRDPSGLSA

FSFGKRRDPSALSALTFGKRGTNPSGYSAFTFGKRGQMDNLHAFSFGKRGMDPSSLGAFSFGKRGRDPSALGAFSFGKR
MGMNAFTFGKREDLEEDGAFEDENDDEKRAKLSSLTSYTFGKRDTQEAAAEDNRLNQEETLRTD

Somatostatin 1
MRCWNLTTVAALLLAGAIVIASAAPSRRNPQNSWPFGMDEEVNTDTLQQSSPLLDKLLDRIYRRLQDRIGNTNNDFSNT
DSRTLTDVDQLIFPGGANELDETIPARPARGKCVGRFVPYMMNC

Somatostatin 2 (Spnp16-like)
MNSAISCLIVMAIIAIFSIGLSLGAGAAISRDEITDSDMDANNVANVAAKMVMHLWNQELRSRFNNAGKRDYDEDAVMI
EEPTKRRRPGCVYDIWKGRGLSRCT

Tachykinin
MASINWTKKLPAVALLICGVLFCNGQAQVSAENTGALKEITDFDDDTQYPELDIDWIEKKDAAFLNPLVSPDLSWENVP
LQYIKRRRNQVFSAGLFGKRSGWNQGHQNGLFGKRSDWLEEYMSGGEDSDADNADSSSQYSKKQWNPNQQTGGLFGKRN
DQLTRRTYNEMLQDLHDRVESAMDKRNAAAATLGRVRTKSSGQHVFRTGGLFGKRSAENPGMQRALWPEEEQRRK

Thyrotropin-releasing hormone 1 (partial)
MQDLMHLRGCIVPLFFLVALSTVCCSADAEFEAAHDITPLEGWPGAEGETAGLLEDGEKRQFAPGKRLLLLGKRQWIGG
MPEMIDMPEERQFSAGKRQFSAGKRQFSAGKRQFSAGKRQWVGGLPEYLEDEDMKRQFSAGKRQFSAGKRQFSAGKRQF
SAGKRDGEDEDELDLMDMFPAPETRQFSAGKRQFSAGKRQFSAGKRQWVGGDAEYDPDEMLDMETRQFSAGKRQFSAGK
R

Thyrotropin-releasing hormone 2 (partial)
VTCVSSAILLLSVVNLWHTVATDDGALTDNENKYLFDRLLLNLRSSENRQQALNDLIRDYIPLDHTSSTRNKNILLEVL
ANLGNVPFDFGGPAEDSPKTGTQDFWKDDDDDLDDGRVNMQLDDLSKRWGAGPGPRGKRFAMEINSLVKRRDKPGPRGK
RQGPRGKKQGPRGKKDKDEDK

Vasopressin / Oxytocin
MACLCWALLLVLWIQGCLACLVTDCPEGGKRSGFNTLRQCQSCGPDGSGQCVGTSICCGDSFGCYMGTPETLICEKEKE
LSSPCRPQGENCLAVAQGQCVANSICCNSRSCAEDLSCASLGIPMRTSSEERSSAGNGFKRNIQDRLKSKLLEVLLRQP

Afnp18 (partial)
MQSSNIAALATLFVFAAVFAQAAYSLSYDAPDRSKRLFWVDKKADVDASNPDKRLFWVDKKTPEKRLFW

Afnp26
MLGMKTLVAYAAIVLALIVSTGTSEDIEDTEELQLPDELIDIKRVDPNYQELLSRLQRARENAALQGLDDKRMASGWKR
AGMAGGWKRGQSAAAGWKRPANAAAGWKRGQALAGGWKRGQPAAAGWKRGQAAAAGWKRDYEPIEVDTRDGDAWRNNQM
KAASGWKRTSAHLIQQNEEDEP

Afnp27
MRMITCTALLCVLLALLDTRQAVCEGGIPVRYQTGTFFGKRQVSDDDVLRALDGPYGRNLLDLMKAWVYRYSQMEAAEQ
LDTN
