# Supplementary material for: Discovery of novel representatives of bilaterian neuropeptide families and reconstruction of neuropeptide precursor evolution in ophiuroid echinoderms
Source: Open Biol. 2017 Sep 6;7(9):170129. doi: 10.1098/rsob.170129 (PMC5627052; doi:10.1098/rsob.170129)
Supplement: Figure S4 (Ophiopsila repertoire) [file rsob170129supp4.pdf]

Figure S4: *Ophiopsila aranea* neuropeptide precursor repertoire

AN peptide
MESRIFLICVAFVVLLAHISGQPVEDQTDEEKRGNRFYSGGLRGGAGRSRGGKRDGEGVVDEVDEVAALADEPDEDKRG
NRFLQNGGGRRKGGRNPGKRTDEQLQELLDEEEKRGNRYLQSTGNGRGRTRQRAGKRDGEDEDFDMDLLDEDKRGNRYL
QAYGGRQRSRTGKRDDDEGFEMELLNEDEKRGNRYLQNGGGRRGGRPGKRDGDDEELDADFLDEEDKRGNRYLSGNGAK
GRGGRKSGKRDGDLEELLEEEDKRGNRFLQSASKGRNKGGRKSG

Calcitonin (short transcript variant)
MRTSVAITIAVCSALYYAVTLVSGLEKRSYIDDESTLPLTGDDLRILADRVDLYNALIAHIETKFPEQFKRGTEKGCSG
FSGCAQLAAGQSALQAMIHGNRASLFGSGGPGKRKRSTDEA

Calcitonin (long transcript variant)
MRTSVAITIAVCSALYYAVTLVSGLEKRSYIDDESTLPLTGDDLRILADRVDLYNALIAHIETKFPEQYKRGGGGCKSF
SGCAQLVIGQNAVRNMMHSNRASIFSGPRGPGRRKRSVGEHTRQVLPLTPDDFRILDDDVELYNAILGQRATTNSKQFK
RGTEKGCSGFSGCAQLAAGQSALQAMIHGNRASLFGSGGPGKRKRSTDEA

CCHamide-like 1 (Arnp25-like)
MDTMPITTRRIGLVRLLLLIVLLTALVYPAYSQAFCANNIPKFCFIHPGKRNSKALKATDTDRMEDGRDQGSMQLSNSD
PSSAISKMEAIIALLLGPVDADETYPDEVQEDTNSLSRSKNDRFLTRLLLSRDHTS

CCHamide-like 2
MTTRGLSITLLLLVLVATLLPSAYTRGRCADPRTCGAAFGKRTGEIQIPQSFDDIQIPLQSNRDFAFKSGSDDKADNVS
FLQSLFKLLPKERQQRIQQDLRKTLQDELFSSRRR

Cholecystokinin 1 (partial)
FGKRNEYGWGQLFGKRDEEQVDYDDFVA

Cholecystokinin 2
MDFKIPLMLVTLSYACVIFLTADALPIGDVFDQEMAEELGIYKPVPERRKDRLLASLQNSGKSLDYGFGMGFGKRSSPL
HRNTAWRDARHRLMELTAEN

Corazonin
MRNCSVGATVLIAVVLGSILCDAHNTFSFKGNNRWNAGKRAQMDTNGLTDRQTTNSFVPKDSPATTLRQLLVDLRDYCD
SLVRVLDESRLETERK

Corticotropin-releasing hormone 2
MKDYLKVMVLYITLTVVVLCLVRSGHGMPDPYLDYKRDTRTAIRNARLTKQTLDDFMRGPGTARERSYLVDPYQMQKRQ
FMTLSGLSPAITQMQKSLSNLRDMMTDQEQQLRELENRKLMSEAGKR

Eclosion hormone 1.1 (Spnp11-like)
MKTVMVSCLVLLLLGESFGAALLDADDDTDAAFALNNLVQRSSDGAEILMERQRRRKSCLVECVTCSRYTLLPTTDCYE
GCNKSAKSSKLAMNTWSACKGMLGQ

Eclosion hormone 1.2 (Spnp11-like)
MKSIIYLCALALFIEAVMPAPGAYLQAFENNDNTEDLEAFDEDLTLRDRRGKTCLTECFACTRMVSKINPSQCVSGCQA
GGRGLKGQAAKTWTICFMALQRRK

Eclosion hormone 2.1 (Spnp15-like)
MDKASVILVLSGLVMILALVGAMPQLLDAQQHADDDIALNDEVAEMFNMARRSSLDYTERAKRARFEKCTMKCVSCNKE
ISGYRFIPCLTACRGGVKSDNNCLRYITK

Eclosion hormone 2.2 (Spnp15-like) (partial)
VMLLNLVGAIPLLTAENNADGDTAFHDERPNLFSLARRTSPLELPGREDFERQQRAQRQLKICTLRCVSCNMEISGYQF
DHCLGGCRLGRINDNNCLRYLTK

Glycoprotein hormone alpha-2.1 (partial)
PSEAQQQAWERPGCHLVGYIKEVRVPGCHMEEVPMNACRGFCVSYSYPSTVARVIETAGAQLTTSVG

Glycoprotein hormone alpha-2.2 (partial)
FPSDKDTLERSRGYKLVTSHGSCCTIASTHDVHVTLQCEDNHQYRDTFKSAAECECAICDQSD

Kisspeptin (partial)
SAVRGRRRGRGRPRSRGSSHGYPQQNKLPFGKRRL

Luqin
MTKVTACICLMVVLLVLQVTTAQGFNRGEGPAKFMRWGKRGDALDSSFQSGDLDIPLFGDSRVICKNTGESGLYRCVAQ

Melanin-concentrating hormone
MQVHLVILAWLATVCLMSCYTASATNLYDTDTEDLLRSLEALGILQEPADEDMLLYAEDEYSKRSSPGDSRRFSVCYDP
IKFKWRRCRQGMASKTRQSVKQ

Neuropeptide-F/Y 1
MKQRIAMDTKILLIVAAVMCCLLSNAHGTNVRRATTGDKALDAILSGQYRSHLRYGKRFSTSLLKNNSPPDMDAFTALW
NEAANNPALKQRLSQYVQQMLQEGSRDVQ

NG peptide / Neuropeptide-S
MAVGIRYAILNLILVLFAARTIFSEVNTHDSTHKVRRNTAGSSGNSIQWTKDDNIDKFRKEIFASLPAELPAILLKSHE
NKEGTNAQDLSALEQVENVGGLDNDARNELALYNYLSRQPARNIYQEGLDKRNGFFFGKRNGFFFGKRDAEACIPCGPQ
NSGQCVMFGTCCSRQFGCYLMTKESEPCMTHHVGTACWDQEFASSCAERRGVCVAENVCCSTRDGACKIDLECSTHKKT
IHYKE

Nucleobindin / Nesfatin (partial)
MAKWQYLLGILSLLTVLCNGLPVVPKEEPEEEEGLLDEEDTGLEYDRYLRQVIKVLEKDPEMRKRMEELSLEDLKEGNF
ARELNFLSTNIRSKLDELKRLEVQRLRTAARQRMEAAAGKGGKRMDPKALEGMVGHVDPASMDRFTDADFERLIKKAAA
DLDEADKERRKEFKRYEMEKELQRRQKMSKMDDDNRVKAEKEFKERKERIRNHPQIKHPGSKAQMQEVWEETDHLDPDD
FNPKTFFALHDTNGDRKLDMFELEALFIKEVEKIYKDSDADPREKFEEMSRMREHVLREIDLDGDKMVSRDEFMKAADQ
AQFEKDDGWKDINQEDQFTEDELQEYNRMVQDRLERKKVR

Orexin 1
MKLLTCLVASLALVVAVIALPTKGNRACCQRTQGCNLRTDCKCLAREVLCRDPSLGLLNMGKRTQETLDAVRLNDDEDA
EARRERRRLARRSFEGKYL

Orexin 2
MPRPGQFITLLLIGIMVYLTGVLGQRVCCRVKGCNIPPDCECPLKKELCKDVSKGILSMGKRTRTYEENVYKQLEQDRD
RHQELRKNKILDTILQLLHTEDQEEDQQVWNPSLSRNLWKTAEYNEDLYEQKPNFYTD

Pedal Peptide 1 (partial)
GKRGFSNMMDPLASGFRKVADKRGFNNMMDPLASGFRKFGGITGKRELNNYIDSLTSDDKRNEGDSDVITRQRRMNLDM
WPGFDNRDHDGGEEVIEKRWFNARKLPGFASKRDIEEDDADNSRWVHAETWPDFSEKRDFVEEELDTAEEKVVE

Pedal Peptide 1 (partial)
RERKGFTETAPKILAGTSVHQFLAGNKRVTMMKFVWSVIALAVMFCFATAVAYTGDAELVQADEADANTLMDDETADKR
GFSNFMDPLSAGFAHKRFNTFMDPLGAGFRPQKRRFNSFMDPLVSGFNYPSKRFNSFMDPLGAGFRPMKRFSHFMDPLQ
SGFHVKREDD

Pedal Peptide 2 (partial)
VGTRHRYRRSSRWCLLQDKQLVIEHTQQAAVCIFGADDQMIEIRMKMAGFHRGAFLLVMVFYLGSGLCYSEDEVPPAAV
PYQQQFVQMKETGGLDHDTQDQDEVDKRRFHYYRDPMMNVFAGKRGFTNMMDP

Pedal Peptide 3 (partial)
MILLRCLGGRPSIILLIFALSLFTSCVEKSVAFDKEESSDQDEDGSSITEEDFLNAEHNIVEDLLPSVEHLLRTELLRL
NQKITNAKDATYRTEVNNESTFLQNLLDSIQNSEDDEETEDILSSFLKDNKGGKFPRFMDPLSIGYRPPGEEDAVLDDL
NGYINKFSAEDAPDKRGHVNFHGPMDALSSGWKRDSENVDDLANDDEKRGHVNFHGPMDALSSGWKRDGNEDEGENLEE
GKRGHVNFHGPMDAL

Pedal Peptide 3 (partial)
SGWKRGHVNFHGPMDALSSGWKRGHVNFHGPMDALNSGWDKRGLVNFNSPMTALHSGWNKRDENSSKENVVEDTETETK
LSHER

Pedal Peptide 3 (partial)

GWKRGHVNFHGPMDALSSGWKRGHVNFHGPMDALSSGWKRGHVNFHGPMDALNSGWNKRDEISGAGDEPQEEDKRGHVN
FHGPMDALSSGWKRGHVNFHGPMDAL

Pigment-dispersing factor

MHTTLILSSAILAVLLGLVASSDSLPDKRIADNDFAQMRSQADRDFEVIAFKNLLREYLGKHGKRDVEKRLSQNDFSQL
RSNLLDEELTKQLIAKFLYHAGRR

Relaxin-like peptide (partial)

ATSTADSAKYCGTDFISVVMETCARHVKRAPLWERLYSASRAKRFADPGFWNTLLESDIEAPMTKKQGSPGLANYCCNT
GCSISDLAMVC

SALMFamide (L-type) (partial)

RRRGIEVPSFNYDAMVKDQQMDHEDREIEERRSGRRSPSMNSGLLFGKRFEETEDFLTDDDDTRQFNVEIRGRNRLPFH
SALMQGKRTPQQDQDNSVKRSRPVFHSGFMMGKRFPMENDNNEIEEYKRKARLRWSNAIQFGK

SALMFamide (F-type)

MARVRNIVLLFASVCCYASISSADRDEYEGTQEITHEQLVNYVRNIAEEVRDLQIELQDNDADIVKSLSKRQAVNPANG
LPMNVPVKMSGFAFGKRDGQLVRRSANAGSKPVKLAGFAFGKRGQLVKRSSDDQLEEEETEKRAAMDAFAFGKRGAHDL
SGLSALSFGKRRDPMGFSGLTFGKRGMGPSGLSAFNFGKRRGDPLSAFDFGKRGMNRDSLSAFDFGKRGRALSAFDFGK
RGMEMPNFLTFGKREDLEEDGAFEDENGDKKRGYNGMSGLTFGKRDTKAAADNSLDQNEETLRTD

Somatostatin 2 (Spnp16-like)

MNSAISCLIFMAIVAIFTIGLSIGAGAAITRDEITDSDMDANNVANVAAKMVMHLWNQELRSRFNNAGKRDYDEDAVLG
EEPLTKRRRPGCVYDIWKGRGLSRCT

Tachykinin

MVAIKNWTKKLPAVALIICGVLFCNGQGQLSPDNNGALNEITEYHDETELDPDWMQPEDEAFLRELSWDNVPLQYAKRR
KNQVFSAGLFGKRSGWNQGHQNGLFGKKRLDNWLEEYMSGREDQNEEAGNTAQFAKRQWNPNQQTGGLFGKRNNDVARR
TYEEMLQSLHDKVESALDKRSAANIGRVRTKSSGQHVFRTGGLFGKRSAEEPGMQRALWPEDEQRRK

Thyrotropin-releasing hormone 1

MQESVLVRGCLVPLLLLIGTVCCLADPADLEGTANDITPLREGWPDNGLENEALEELENEVKRQFSPGKRLLLLGKRQA
LGGMPEMVDLPETRQFSAGKRQFSAGKRQFSAGKRQFSAGKRQWVGGKLPLQFEDEDIKRQFSAGKRQFSAGKRQFSAG
KRQFSAGKRQFSAGKRDWEDELTPEDLLDLVPAPETRQFSAGKRQFSAGKRQFSAGKRQWVGGLPEYDPEDMLDMETRQ
FSAGKRQFSAGKRQFSAGKRDETNILDDILEADPDADDALEE

Vasopressin/Oxytocin (partial)

MACLCWALLLVLWIQGCLACLVTDCPEGGKRSGYNSLRQCQS

Oanp18

MHSSYIAALATIFVFAAVFAQAAYSLSYDGAQRSKRLFWVDKKAEDSQPDKRLFWVDKKTPEKRLFWVDKKTPEKRLFW
VDKKADETQYVPVENIDKVADCMINVVSQYAKHIEAECRQPGVLDESCVKRIKGKRTQANINCLTDTDGSN

Oanp26

MLGMKTLLGYAAIVLALVVSTGTSEDIEEPETEELQLADENDLMDIDPPDYQELLMQLQRARENGLQDLDDKRMAAGWK
RGQSAAAGWKRGQGAAAGWKRPASAAAGWKRGQSAAAGWKRGQSAAAGWKRGQGAAAGWKRDYPIDVDTRGGENWRNSH
VKSASGWKRTARSANRQQNAEDIH

Oanp27

MRMITCAALFCVLLALLDTRQNGCEAGIPVRYQTGTFFGKRQVSDEDVLRALDGPYGRNLMDLMKAWVYRLHQMEAAEQ
VESN
