# Supplementary material for: Discovery of novel representatives of bilaterian neuropeptide families and reconstruction of neuropeptide precursor evolution in ophiuroid echinoderms
Source: Open Biol. 2017 Sep 6;7(9):170129. doi: 10.1098/rsob.170129 (PMC5627052; doi:10.1098/rsob.170129)
Supplement: Figure S10 [file rsob170129supp10.docx]

**Alignment of pigment-dispersing factor-type precursors**

Ovic --MHITLMFTSAIVAVL-------IGLAASSDL-----LEDKRIADNDFAQMRSIADRK-NEAIAFRNLL
Arub -MTQLTLLAVCGSVLLL-------VGLTHCTDE-----QREKRLGDNDFFQATYNDAQARQRQRVLQSYL
Acal MFGYRSLLVL--LVTLSLCLL---LQSSHCSA--------VRTYGNDLD-------ARARR---EIISLA
Pdum -MGSSKT--V--QVAVVVCLVSMFVMQVVCYP------TQRSNLRNSLT-------DADRQ---EILRYA
Erow --------MKSVV--FAICLII-FAVVLV--------------SIN-AE---------YGQ---------
Nlug --MKSTYIFGFILLGLVLCVQTVLSMRYS---------ADDSSYLDTPQ--------YTAR---DLAKWL
Bmor -MKSVTLLFFLFLME-----ASTYSVANSEAKIKLNRKVSESSYGS-DE--------QYIR---QIHSLV
Dmel -MARYTYLVALVL--LAICCQWGYCGAMA--------MPDEERYVR-KE---------YNR---DLLDWF

**---------Mature peptide--------**
Ovic SQI-------------------LK--EQGKRDVQKRLSQNDFSQLRSNVLDQELTKQLIARFLSEAGRR-
Arub DDR-------------------MA--SVGKRDGLKRNFDED--VYHQEGLDNEFVRRLMAKYFDGVARRR
Acal ARL---------------IKLSMYGPE--DDSFVKRNGGTADALYN------------LPDLEKI-GKR-
Pdum AKI---------------ARIAMGDNVDFKAGPNKRNPGTLDAVLD------------MPDLMSL-GRK-
Erow ---------------DSEYD-TLASKLLSRVLAQKRNAELINSLLN------------LPQKLQEAGK--
Nlug --------------------------MESAQRTEKRNREITNSLLN------------LPKTMMEAGRK-
Bmor NAYR-----EDNSMLGENFIIETKALIDTKYRTWKRNADLINSLLA------------LPKDMNDAGR--
Dmel NNVGVGQFSPGQVATLCRYPLILENSLGPSVPIRKRNSELINSLLS------------LPKNMNDAGK--
