# Supplementary material for: Discovery of novel representatives of bilaterian neuropeptide families and reconstruction of neuropeptide precursor evolution in ophiuroid echinoderms
Source: Open Biol. 2017 Sep 6;7(9):170129. doi: 10.1098/rsob.170129 (PMC5627052; doi:10.1098/rsob.170129)
Supplement: Figure S11 [file rsob170129supp11.pdf]

AN peptide

```
Op_exim_1   METRIALISVTFVVLLAHISGAPVEEQLEEDKRGNRYLSGPGRSRGRN---GKRAEGDEIVD-EVADFANLA-DETDEEKRGNMHLQGGG---RAR
Op_exim_2   METRIALISVTFVVLLAHISGAPVEEQLEEDKRGNRYLSGPGRSRGRN---GKRAEGDEIVD-EVADFANLA-DETDEEKRGNMHLQGGG---RAR
Am_laud     MESRIFLLSLAFVVLLAQISGAPVDNELEEEKRANFHQNGNGRSRGRGK--PKR-DGE-IVD-EINEIAQLIDDETDEEKRGNMHYTGGGRGGKAG

Op_exim_1   GR-NRNGKRAEGDENFELDFLDSEEKR--------------------------------------GNRYMT--GTGG-RQRGRNGKRT-DEELQE
Op_exim_2   GR-NRNGKRAEGDENFELDFLDSEEKR--------------------------------------GNRYMT--GTGG-RQRGRNGKRT-DEELQE
Am_laud     GR-GRTGKRD--DEGFDEDFDIDEDKRGNRHLTGGGAAGGRRRKQGKRDDEGEFETDLDLEQDKRGNMHLAGAGRGGSRGRNRGGKRD-DGEFDE

Op_exim_1   LLDEEDKRGNMYLQG-GKQRGR---NGKRAD----EDAEALNMEELLDEDEKKRKYVLERW-----------
Op_exim_2   LLDEEDKRGNMYLQG-GKQRGR---NGKRAD----EDAEALNMEELLDEDEKKRKYVLERW-----------
Am_laud     EGEEAEKRGNMYLQGVGNNRGRGRGKGKRTDGEP-EDA----LDALLNDDEKRGNMFLAGGGRGGRGRGKGK
```

Calcitonin (short)

```
Am_cipu      MRTSVAITIAVCSALYYAVTLVSG--LEKRSF--IVDE-TLPLTGDDFRILADKVDLYNNLIGHLQNQFPEQ--F-KRGS--EKGCAG-FSGCAQ
Am_cons_1    MRTSVAITIAVCSALYYAVTLVSG--LEKRSY--IVNEESLPLTGDDFRILADKVDLYNTLISTLQDKFPEQ--F-KRGT--EKGCAG-FSGCAQ
Op_filo      MRTSLAITVAVCSALYYAVTLVSG--LEKRSF--IDDDALLPLTGDDFRVLADKVDIYNALISRIASQFPEQ--F-KRGS--DKGCAG-FSGCAQ
Mi_grac      ----------------------------------------LPLTGDDLRILADKVDIYNALISRVATKFPEQ--F-KRGT--EKGCAS-FSGCAQ
Am_squa      MRTSLAITVAVCSVLYYAVTLVYG--LEKRSF--IDDEALIPLTGDDLRVVADKIDLYNALISHIANKYPEQ--I-KRG----TGCKG-FAACAQ
Op_resi      MRTSVAITIAVCSALYYAVTLVSG--LEKRSY--IDNASSLPLTGDDLRVLADKVDLYNALISHIESKFPEHMGF-KRGGG-GKGCAS-FTGCAQ
Op_savi      MRTSVAITIAVCSALYYAVTLVSG--LEKRSY--IDNASSLPLTGDDLRVLADKVDLYNALISHIESKFPEQ--F-KRGG--GKGCAG-FTGCAQ
Op_abys      MKTSVAITIAVCSALYYAVTLVSG--LEKRSY--IDNESSLPLTGDDLRILADKVDLYNALISHIESKFPEQ--F-KRGG--DKGCAG-FTGCAQ
Op_angu      MRTSVAITIAVCSALYYAVTLVSG--LEKRSYNLIEQESSLPLTGDDLRVLADKVDIYNALISHIESKFPEQ--F-KRG----KGCRG-FTGCAH
Op_caes      MRTSVAITIAVCSALYYAVTLVSG--LEKRSYNTIDKESSLPLTGDDLRVLADKVDIYNALISHIESKFPEQ--F-KRG----KGCGG-FTGCAQ
Op_scha      MRTSVAITIAVCSALYYAVTLVSG--LEKRSY--IDDE-TLPLTGDDLRILADKVDLYNALIAHIENKFPEQ--F-KRGT--EKGCAG-FSGCAQ
Op_lame      MRTSVAITIAVCSALYYAVTLVSG--LEKRSY--IDDE-TLPLTGDDLRILADKVDLYNALIAHIENKFPEQ--F-KRGN--EKGCSG-FSGCAQ
Op_impr      MRTSVAITIAVCSALYYAVTLVSG--LEKRSY--IDDESTLPLTGDDLRVLADKVDLYNALIAHIQNKFPEQ--F-KRGS--EKGCSG-FSGCAQ
Op_vall      MRTSVAITIAVCSALYYAVTLVSG--LEKRSY--ID-EPVLPLTGDDLRVLADKVDLYNALIFRIQTQFPEQ--F-KRGG--ERGCAG-FSGCAQ
Op_brev      ----------------------SS--LEKRSY--IDDNIALPLTGDDLRILADKVDLYNALISEIHSQFPEQ--F-KRGG----GCRA-FGGCAQ
Op_brev      ----------------------SS--LEKRSY--IDDNIALPLTGDDLRILADKVDLYNALISEIHSQFPEQ--F-KRGN--EKGCSG-FSGCAQ
Ba_hero      MKTSVAITIAVCSALYYAVTLVSG--LEKRSY--IDDETALPLRGNDLRILADKVDLYNALIAHVENQFPEQ--F-KRGG---EGCSG-FSGCAQ
Cl_cana      ------------SALYYAVTLVSG--LEKRSY--IDDETRLPLTGNDLRVLADKVDLYNAFIAHIENQFPEQ--F-KRGG---EGCSG-FSGCAQ
Op_liod      MRTSVAITIALCSALYYAVTLVSG--SERRSY--VDDETSLPLTGNDIRVLADNVDLYNALIAHIQNQFPEQ--FIKRDG---SGCRG-FSGCAQ
Op_prol      MRTSVAITIAVCSALYYAVTLVSG--LERRSY--IDDQTTLPLTGDDLRVLADKVDLYNALILHVQNQFPEQ--FIKRGNGDNGGCKG-FSGCAQ

Am_cipu      LAAGQSALQAMIHGNRASLFGS-GGPGKRKRS-AD-------EA--
Am_cons_1    LAAGQSALQAMIHGNRASLFGS-GGPGKRKRS-TD-------EA--
Op_filo      LAAGQSALQAMIHGNRASLFGS-GGPGKRKRS-AE-------EA--
Mi_grac      LAAGQSALQALIHGNRASLFGS-SGPGKRKRS-AE-------EA--
Am_squa      LADGQSALQAMIHNNRASLFGS-SGPGKRKRS-AEL------EA--
Op_resi      LEAGQSALQAMIHANRASLFGS-SGPGKRKRS-AE-QQQPEQEA--
Op_savi      LEAGQSALQAMIHANRASLFGS-GGPGKRKRS-AE--QQPEQEA--
Op_abys      LAAGQNALQAMIHANRASLFGS-SGPGKRKRS-ADQQQQMQQEA--
Op_angu      LDAGQTALQAMIHADRASLFGS-DGPGKRKRS-VD---QTQQDA--
Op_caes      LDAGQNALQAMIHANRASLFGS-SGPGKRKRS-TD---QVQRDA--
Op_scha      LAAGQSALQAMLHGQRASMFGS-SGPGKRKRS-AE-------EA--
Op_lame      LAAGQSALQAMIHGNRATLFGS-GGPGKRKRS-AE-------EA--
Op_impr      LAAGQSALQAMIHGNRASLFGS-GGPGKRKRS-TD-------EA--
Op_vall      LAAGQSALQAMIHNGRASLFGS-GGPGKRKRS-AE-------EA--
Op_brev      LAIGQNAVRNLIHSNRASIFSSPRGPGKR-----------------
Op_brev      LAAGQSALQAMIHSGRASLFGS-SGPGKRKRS-AE-------EA--
Ba_hero      LAAGQSALQAMIHSGRASLFGS-GGPGKRKRS-AE-------EA--
Cl_cana      LAAGQSALQAMIHNGRASLFGS-GGPGKRRKRLAE-------EA--
Op_liod      LAAGQSALKAMLHHGRASLFGS-TGPGKRRRS-AE-------EA--
Op_prol      LAAGQSALQAMIHSGRASLFGS-GGPGKRKRS-AE-------EA--
```

Calcitonin (long)

```
Am_cipu     MRTSVAITIAVCSALYYAVTLVSGLEKRSF--IVDE---TLPLTGDDFRILADKVDLYNNLIGHLQNQFPEQFKRGG-GGCRGFSACAQLAIGQD
Am_cons_1   MRTSVAITIAVCSALYYAVTLVSGLEKRSY--IVNEE--SLPLTGDDFRILADKVDLYNTLISTLQDKFPEQFKR-G-GGCRDFSACAQLAIGQD
Op_savi     MRTSVAITIAVCSALYYAVTLVSGLEKRSY--IDNAS--SLPLTGDDLRVLADKVDLYNALISHIESKFPEQYKRGG-GGCWYFGGCAQLVIGQN
Op_angu     MRTSVAITIAVCSALYYAVTLVSGLEKRSYNLIEQES--SLPLTGDDLRVLADKVDIYNALISHIESKFPEQFKRGG-GGCKHFTGCAQLVIGQN
Op_caes     MRTSVAITIAVCSALYYAVTLVSGLEKRSYNTIDKES--SLPLTGDDLRVLADKVDIYNALISHIESKFPEQFKRGG-GGCKHFTGCAQLVIGQN
Op_impr     MRTSVAITIAVCSALYYAVTLVSGLEKRSY--IDDES--TLPLTGDDLRVLADKVDLYNALIAHIQNKFPEQLKRGG-GGCKSFSGCAQLVIGQN
Ba_hero     MKTSVAITIAVCSALYYAVTLVSGLEKRSY--IDDET--ALPLRGNDLRILADKVDLYNALIAHVENQFPEQFKR-G-GGCRGFGGCAQLAEGQN
Op_wend     MRTSVAITIAVCSALYYAVTLVSGLEKRSY--TDDETPATLPLTGNDLRVLADKVDLYNALIAHIETQLPEQFKR-G-GGCKGFGGCAQLAEGQN
Cl_cana     ------------SALYYAVTLVSGLEKRSY--IDDET--RLPLTGNDLRVLADKVDLYNAFIAHIENQFPEQFKR-G-GGCKGFSGCAQLTEGQN
Op_prol     MRTSVAITIAVCSALYYAVTLVSGLERRSY--IDDQT--TLPLTGDDLRVLADKVDLYNALILHVQNQFPEQFKR-G-WGCKVFGGCAQLVEGQN
As_tubi     MRTSVALTVAICSVMYYAVTLVSGFEKRSY--IDYEP--TLPLSGENLRELASNVDLYNNILTRVVNQYPEEFKR-G-GGCRGFSGCAQLAEGQS
As_bidw     MRTSVALTVAICSVMYYAVTLVSGFEKRSY--IDYEP--TLPLSGENLRELASNVDLYNNILTRVVNQYPEEFKR-G-GGCRGFSGCAQLAEGQS
Op_oedi     MRTSVALTVAVCSVMYYAITLVSGFEKRSY--IDYEP--TLPLSGEDLRVLASNVDLYNNILTRVVNQYPEEFKR-G-GGCRGFSGCAQLAEGQS
As_love     MRTSVAIIIAVCSIMYYAVTMVSGFEKRSY--IDDEP--TLPLSGEDLRILADNVDLYNNILTRVVNQYPEQFKR-G-GGCRGFSGCAQLAEGQS

Am_cipu     AFRNMMHNNRASLFTSARGPGRRKRSIDQQ--QVLPLTGDEFRLLADQVELYNDILGQIASTNLQQF-KR-GSER-GCAGFSGCAQLAAGQSALQ
Am_cons_1   AFRNMMHKNRASIFTSARGPGRRKRSIDQQ--QVLPITGDEFRLLADEVELYNDILSQIASTNLKQF-KR-GTEK-GCAGFSGCAQLAAGQSALQ
Op_savi     AVRNMIHNNRASVFSSPYGPGRRKRSVSSG--DVLPMSSNDFKLLADDVELYKDILGQITTANSKQF-KRGGGK--GCAGFTGCAQLEAGQSALQ
Op_angu     ALRNMMHKNRASVFKSPYGPGRRKRSLNDG--DELQIPNDDYRILADDVELYNVILGQITTTNSNEF-KR--GK--GCRGFTGCAHLDAGQTALQ
Op_caes     ALRNMMHKNRASVFKSPYGPGRRKRTVSDG--EELQIPNDDYRILADDVELYNVILGQITTTNSNEF-KR--GK--GCGGFTGCAQLDAGQNALQ
Op_impr     AVRNLIHSNRASIFSSPRGPGRRKRSVDVQTEQVLPISGDDFRILADELELYSAILGQMGTRNPKQF-KR-GSEK-GCSGFSGCAQLAAGQSALQ
Ba_hero     AVRNLMHINRASVFSGPRGPGKRKRSVDDQTEQYLPLTGDDLRILADEVDLYNAVIKQIASTYPEQF-KR-GGE--GCSGFSGCAQLAAGQSALQ
Op_wend     ALRNLMHLNRASVFSGPRGPGKRKRSVDDQTEQYLSITGDDLRILADEVDLYNAVLNQIATKYPEQF-KR--GE--GCSGFSGCAQLAAGQSALQ
Cl_cana     AVRNLMHSNRASIFSGPRGPGKRKRSVDDQTVQLLPITGDELRILADEVDIYNAVISQIASQYPEQF-KR-GGE--GCSGFSGCAQLAAGQSALQ
Op_prol     ALRNLILSNRAAIFSSPLGPGKRKRSVDDQIEQLLPITGDELRTLAGEVDLYNAIISPIATIYPEQFIKRGNGDNGGCKGFSGCAQLAAGQSALQ
As_tubi     ALRNMMHNNRASMFSGAGGPGKRKRSINDQ--SLLPITGDDLRTVADEVDLYNAIISQTAIRYPEQF-KR--GE--GCKGFSGCAQLAAGQSALQ
As_bidw     ALRNMMHNNRASMFSGAGGPGKRKRSINDQ--SLLPITGDDLRTVADEVDLYNAIISQTAIRYPEQF-KR--GE--GCKGFSGCAQLAAGQSALQ
Op_oedi     ALRNMMHNNRASMFSGAGGPGKRKRSINDQ--SLLPITGDDLRTVADEVDLYNAIISRTAISYPEQF-KR--GE--GCKGFSGCAQLAAGQSALQ
As_love     ALRNMMHNNRASIFSGATGPGKKKRSIDDQ--SVLPITGNDLRSVADEVDLYNAIISRTAIRYPEQL-KR--GE-GCKGFSGCAQLAAGQTALQ

Am_cipu     AMIHGNRASLFGSGGPGK-RKRSADEA-----
Am_cons_1   AMIHGNRASLFGSGGPGK-RKRSTDEA-----
Op_savi     AMIHANRASLFGSGGPGK-RKRSAEQQPEQEA
Op_angu     AMIHADRASLFGSDGPGK-RKRSVDQT-QQDA
Op_caes     AMIHANRASLFGSSGPGK-RKRSTDQV-QRDA
Op_impr     AMIHGNRASLFGSGGPGK-RKRSTDEA-----
Ba_hero     AMIHSGRASLFGSGGPGK-RKRSAEEA-----
Op_wend     AMIHNGRASLFGSGGPGK-RKRSAVEA-----
Cl_cana     AMIHNGRASLFGSGGPGKRRKRLAEEA-----
Op_prol     AMIHSGRASLFGSGGPGK-RKRSAEEA-----
As_tubi     AMIHNNRAPLFGSSGPGK-RKRSAEEV-----
As_bidw     AMIHNNRAPLFGSSGPGK-RKRSAEEV-----
Op_oedi     AMIHNNRAPLFGSSGPGK-RKRSAEEV-----
As_love     AMIHSNRAPLFGSSGPGK-RKRSVEEVSK---
```

CCK 1

```
Am_cons_1   ------PLLLAIACLFLTPAATLPSKTGK---TRDLSHTDIERLILNTIETVESQAINRKSGTTNDEEVGGTPWKAENTWKTPTLPDLIKDWKESVKERQENL
Am_cons_2   MTGDVLPLLLAIACLFLTPAATLPSKTGK---TRDLSHTDIERLILNTIETVESQAINRKSGTTNDEEVGGTPWKAENTWKTPTLPDLIKDWKESVKERQENL
Am_squa     MTGDVLPLLLAIACLFLTPAATLPSKTGT---SRDLSHTDIERLILNTIETVEAKAVNRDRGDSNDDEVGGTPWKADNTWKTPTLPDLIKDWKESVKERQENL
Op_savi     MTGDVLPLLLAIACLFLTPAASLPSKTGNSKSSRDISHTDLERLILNTIETVETQ--NR----PHDEETEGTPWKSEN---TPTLPDLIKDWK----ERRESL
Op_abys     ---------------------------GNSKSSRDISHTDLERLILNTIETVETEALNR----PHDEETEGTPWKAEN---TPTLPDLIKDWK----ERRENL
Op_angu     MTGDVLPLLVATACLFLSSCAALPSKTGNTKLGRDISHTELERLILNTIETVKNQAFSR----ADEEETEGTSWDNEN---TPTLPDVLKDWT----GRHKSL
Op_reti     --------------------------------------------------TVEAQAINSRQ--THD-EVEGTPWKAENTWKTPTLPDLIKDWKESVKERQESL
Ba_hero     -----------IACLFLTPAASLPSKTGNSKASRDISQTDIERLILNTIETVQAQALNRQSG-THD-EVGGTPWKAENTWKTPTLPDLIKDWKESVKERHESL
Op_perf     MTGDVLPLLLALVCLFLTPAASLPSKSGNSKASRDISQTDIERLILNTIETVQAQAINRQSG-TRD-E--GTPWKVENTWKTQTLPDLIKDWKENVQERQESL
As_love     -------------CLFLTPAASLPSKTGSLKAGRDISQTDLERLLLNTIATAQVQVINRQSG-THD-KLGDT-WKAENTWKTPTLPDLIKDWKASVKEQQDNL

Am_cons_1   -AEND-LLRDVPETD---RNLNDKRS--RDYGWGMAFGKRGSRTQKER-----LQTRNKE--YGWGTFFGKR--N-EYGWGHMFGKR--D-EEDIDYDDFVA-
Am_cons_2   -AEND-LLRDVPETD---RNLNDKRS--RDYGWGMAFGKRGSRTQKER-----LQTRNKE--YGWGTFFGKR--N-EYGWGHMFGKR--D-EEDIDYDDFVA-
Am_squa     -AEND-ILRDLPVPDADLSDLNEKRS--RDYGWGMAFGKRGSRTQNER---HKMETRNKE--YGWGTFFGKR--G-EYGWGHMFGKRNVN-EDEVDYDDFVA-
Op_savi     AAGDN-FLRGLEEDD---ADIVDKRG--KDYGWGFLMGKRNSRTQSQRQQSFQMESKRDH-MYGWGTTFGKRSGDSKYGWGQFFGKR--NGDEEVDYDDFVAL
Op_abys     -AGNN-LLGDIEEDD---TDIIDKRG--KDYGWGFLMGKRSSWTQSKRQQSYKMQTKKDSPNYQWGMTFGKR--NHNYGWGQIFGKR--D-NEEADYDDFVAL
Op_angu     -ADNK-LLNGFSEDDA--VNLLDKRSRPKDYNWGILFGKRSQLSHRQREQDFKVETKKSN-DYGLGLLFGKRS-NGQYGWGNLFGKR--DVADDGDYDDFVAL
Op_reti     -AENDLLLGDIADED---TDITEKRN--RDYGWGMAFGKRGSRTQNQRQ-SHKVESRNKE--YGWGTFFGKR--N-EYGWGHMFGKR--D-EEQVDYDDFVA-
Ba_hero     -AEND-LLRDIPEED---EDIAEKRN--RDYGWGMAFGKRGSRTQNQRQ-SHKKETRNKE--YGWGTFFGKR--N-EYGWGHMFGKR--D-EEQVDYDDFVA-
Op_perf     -AEND-LLRDLPQDD---EDIMEKRN--RDYGWGMAFGKRGTRTQNQRQ-SHKMETRNKE--YGWGTFFGKR--N-EYGWGHMFGKR--D-EEQVDYDDF---
As_love     -AEHG-IFQDIPEE----EGLNDKRS--RDYGWGMAFGKRGSRTQNERQ-NHKMETRNKE--YGWGTFFGKR--N-EYGWGHMFGKR--N-EEQMDYDDFVA-
```

Kisspeptin

```
Am_squa    MRSCCMSRRILPLAWFICFSVAFLPRLSIAENRNLDQNDISSLLVADEDQEHSSDLGFQSANILLDVIERILDRLESSAVPPLQ--ETNDFLQSAP-ETSWPPLENDFLS
Op_resi    MRSCFML-RILPVA-FICF--FGLPILSIADSRNLDQ-DIP-LIVREEEESNSSDLGLQSASILLDAIERLLNRLDSSAVPP----QDNDI-QRAP-ETSWPPLENDFLS
Op_savi    MRSCFML-RILPVA-FICF--FGLPLLSIADSRNSDQ-DIP-LIVREGEELNSSDLGLQSASILLDAIERLLNRLDSSAVPP----QDNDI-QRAP-ETSWPPLENDFLS
Op_abys    MRSCFLL-RILPVA-FICF--FGLPILSIADSRNSDQ-DIP-LIVREEEEPHSTDFELQSTGFVLDALERLLHRLDASAVPP----QDNDI-QSAPGETSWPPLENDFLS
Op_reti    ------------------------------------------------EQQNSPDYGLHSANILLDVIERILDRLESSAVPP----EDNDI-HSAP-ETSWPPLENDFLS
Op_impr    MSCCSML-RIVPVA-VICF--FVLPFLSIADDRNSDQ-DIP-LVAAEDEEQNSSDFGLHSASILLNVIERILDRLESSAVPP----QDNDI-YSAP-ETSWPPLENDFLS
Op_fune    ---------------LICS--LGLPVISSADTRSTDQ-DIP-FF-RGEEQENPSDFGIHSTSFLFDVIERILDRLESSALPP----QDNDI-NIAP-ETSWPPLENDFLS
As_bidw    MRSCSML-RILPVA-LICS--LGLHVLCSGD-RNTQQ-DLP-LN-RGEQQQNPSGFRLHSADILLDVIEQIVDRLESSALPP----SDNDI-YNAP-ETSWPLLEQDMLS
Op_oedi    -------------------------------------------------------------------------------------------------------LEQDMLS
As_love    MRSCSML-RILPVA-LICS--LGLPVLCSGD-RHTEQ-DTS-LN-RGDEQQNSSDFGLHSASILLDVLERILDQMDSSAVPP----NENDI-YSPP-DASWPPLERDLLS

Am_squa    RDVIYLPKSSSSSNTVTLG-ITSIGKSRHRGFAPCNKG-LCRVIRG--RR-R--NANAGARMLPFGKRSNTVRNS-EDSGTSSV-RGRRRGRGRPRSRGS---FP-PQKPLPFGKRRL
Op_resi    RDHIYLPK-SSSSNALKLKHMTSYGKSQR---TSCTEGFLCRVIRG--R-----NPNAGARALPFGKRGGNKRNSGDESGTSSV-RGRRRGRGRPP------PVPRRQRQLPFGKRRV
Op_savi    RDHIYLPK-SSSSNALKLKHMTLYDKSQR---TSCSEGFLCRVIRG--RG-RG-NANAGARALPFGKRGGNKRNSGDESGTGSV-RGRRRGRGRPRSRGI--PQP-QQKQLPFGKRRV
Op_abys    RDHIYLPK--SSSNALKFG-MTSYGKSQR---ASCSGGIICRVIRG--RGGRG-NVNAGARALPFGKRGGNKRNSGDESGTSSV-RGRRRGRNRPRSRGSSNGFP-QQNKLPFGKRRV
Op_reti    RDHTYLPK--SGTNSLMLG-MTSYGSRH----VPCSGG-LCRVIRG--RQ-RGNNVNAGARALPFGKR-ANVRNS-DEASTSSV-RGRRRGRGRPRSRGS--SYP-QQNKLPFGKRRL
Op_impr    RDHVYLPK--SSSNALTFG-VTSYGKNQR---TSCSEG-LCRVIRG--RQ-RG-NANAGARALPFGKR-GSVRNS-DD-GTSAV-RGRRRGRGRPRSRGSL-GYP-KQ-KLPFGKRRL
Op_fune    RDHVYLPK--SSSKALTLG-VTSYGNWQR---APCT-GIMCRVTRG--KY-RG-NINAGARALPYGKR--------------------------------------------------
As_bidw    QEPLYPPM--SSGNGLTYG-MTSYEKRQQ---APCN-DMMCAMKRGSRRQ-RI-NPNVNGNMLPFGKR-SDARNS-DEAGMRSV-RGRRRSRAKPRTRGQ------QQKKLPFGKRRL
Op_oedi    QEPLYPPM--SSGNGFTYG-MTSYENRQQ---APCN-DVMCAMKRG--RQ-RI-NPNVNGNMLPFGKR-SGARNS-DEAGMRSV-RGRRRGRAKPRTRGH------QQNKLPFGKRRL
As_love    QDPLHPPK--SLSNDLTFS-MTSYEKRPQ---APCN-DVMCRMVRG--KQ-RV-NANAGSRMLPFGKR--DNRNS-DEAGMRSAFRGRRRGRGRPRTRGH------QHNKLPFGKRRL
```

NG peptide

```
Am_cipu      --------------------------------------SPVGSSGNS-IQWTKDDNINKLRKEIIASLPADIPAILLKPQTQENKEDT-PQDWTA
Am_cons_1    MALGIKFYTILNLILVLLAARTIFGEVNTHETSNKVRRSAVGSSGNS-IQWTKDDNINKLRKEIIASLPADIPAILLKPQTQENKEDS-PQDLTA
Am_cons_2    MALGIKFYTILNLILVLLAARTIFGEVNTHETSNKVRRSAVGSSGNS-IQWTKDDNINKLRKEIIASFPADIPAILLKPQTQENKEDS-PQDLTA
Op_filo      --VGIRYCVIFNLILVLLAARTIFGEVNTHDISHKVRRSPVGSSVNS-FQWTTDDNINKLRKDILASIPDDIPAILL----QDDQEDT-PQDLTA
Mi_grac      MAVGIRYYAILNLILVLLAARTIFGEVHTQDSTHKVRRSPVGTSVNS-FQWTKDDNINKLRKDILSSIPDEIPAILFNPQTQEDEKDT-LQDLSA
Am_squa      MAVGIRYYAIFNLILVLFATQTVFGEVNTHESSNKVRRSPVGSPVNS-FQWTKDNNINKLRKDILASIPADYQAALLKKHSEEEQEDT-PQDLAA
Op_resi      MAVGIRY-AILNLVLVLIAARTIFCELHTQDSTHKVRRNTAGVSGNS-NQWTKDDNIHKLRQEIIASLPADLPAYLLNPKIHESKDQGTSQELSA
Op_savi      MAVGIRY-AILNLVLVLIAARTIFCEIHTKDSTHKVRRNTAGVSGNS-NHFTKDDNIHKLTQELIASLPADHPAYLLNPKIHESKDQGTSPELSA
Op_abys      MAVGIRS-AILNLVLALIAARTTFCEVHTEDLTHKVRRNTIGVPGNS-NQWTKDNNIHKLRQEIIAALPADLPAYLLNPKIHESKDQGTSPELSA
Op_angu      MAGGIRH-VILNLLLVLIAARISVSELHISDATQKVRRSTGGITGNS-NQWTKDESINKLRQDFIASLPGNLPAYLIDSKLLKGKDKDVSPALT-
Op_caes      MAGGIRH-AILNLLLVLIAARISVSELQISDATHKVRRYTGGITGNS-NQWTKNESINKLRQELIASLPANLPAYLLHSKISEGNDKDLSPELSA
Op_reti      MAVGIRY-AILNLILVLIAARTTFGEVNTHDSSHKVRRNTGDSSGNS-LQWTKDNNIDKLRKEIFASLPADLPAVLLNSQTHEGAEGT--HDLSA
Op_lame      MAVGIRY-AILNLILVLIAARTTFGEVNTHDSSHKVRRNTGDSSGNS-LQWTKDNNIDKLRKEIFASLPADLPAVLLNSQTHEGKEGT--QDLSA
Op_impr      MAVGIRY-AILNLILVLIAGRTIFGEVITHDSTHKVRRNTDGPSGNS-IQWAKDDNIDKLRKEIIASLPADLPAVLLKSPTHERKEGT--QDLST
Op_vall      MAVGIRY-AILNLILVLLAVRTIYGEDNTHELTKKVRRDTGGSSGHSQTQWTKDNNINKLRKEIFA----DLPAILLQSQTHEDNEGSHLQELSA
Op_brev      MAVGIRY-AIFNLILVLLAARTIYGNEDTHDLTKKVRRDTIDSSANS-NQWIENDNIDKLRKEIFASFPADIPAILLKSQTHEESEGTNIKDLPA
Ba_hero      MAVGIRN-AILNLILVLFAARTIFGETNTNDLTHKVRRDTGGSSGHS-NHWTKNDNIDKLRKEIFASLPADLPGILLNSKTHETQEGTHTDDLSP
Op_wend      ----------------------------------------------------KNDNIDKLRKEIFASLPADLPGILLKSKIHASQEGTQTDDLPS
Cl_cana      -----------------------------------------------------------------------------------------------
Op_exim_1    -----------------------------------------------------------------------------------------------
Op_liod      -----------------------------------------------------------------------------------------------
As_tubi      ----------------L----TRYGEIRV------------GTRDTQ-TIGQKKNNVNKFRNEILASLPANLPNILLDSQTRDTESTQ-TEELSP
As_bidw      ----------------L----TRYGEIRV------------GTRDTQ-TIGQKKNNVNKFRNEILASLPANLPNILLDSQTRDTESTQ-TEELSP
As_bidw      ----------------L----TRYGEIRV------------GTRDTQ-TIGQKKNNVNKFRNEILASLPANLPNILLDSQTRDTESTQ-TEELSP
Op_oedi      MAVGIRY-AILNLILVLFAA-TLFGEIVTDDLTYKVRRDTGGNSGHS-NYWSKKNNVNQFRNEILASLPANLPNIILDSQTRDTESTQ-TEELSP
Op_oedi      MAVGIRY-AILNLILVLFAA-TLFGEIVTDDLTYKVRRDTGGNSGHS-NYWSKKNNVNQFRNEILASLPANLPNIILDSQTRDTESTQ-TEELSP
As_love      MAVGIRY-AILNLILVLFATSSIFGEIVTDDLTYKVRRDTGGTSENS-KYWSNNNNIDEFRNEILASLPADIPSILLNSQTRETKSTQ-TEELSP
```

```
Am_cipu     L--EQ--VGKVAAGQAIDA--DGRDN--LYNFLSRQSAR-NNYQ-GLE-------SKRNGFFFGKRNGFFFGKRDTEAVKSDSCVSCGPQNSGQC
Am_cons_1   L--EQ--VEKVAIGQAIDA--DARDN--LYNFLSRQSAR-NNYQ-GLE-------SKRNGFFFGKRNGFFFGKRDTDAVKSDSCVSCGPQNSGQC
Am_cons_2   L--EQ--VEKVAIGQAIDA--DARDN--LYNFLSRQSAR-NNYQ-GLE-------SKRNGFFFGKRNGFFFGKRDTDAVKSDSCVSCGPQNSGQC
Op_filo     L--KQ--VGRVTGGRAITNT-DARDN--LFHFLSRPSGR-NNYQEGLQ-------SKRNGFFFGKRNGFFFGKRDTEAVESNLCVRCGPENSGQC
Mi_grac     L--EQ--VGRVASGHTITDTDDARDN--LLHFLSRQTARNNNHQEGLE-------SKRNGFFFGKRNGFFFGKRDTEAVKSDLCVRCGPDNSGQC
Am_squa     L--DQ--VGRL--GHET----DARDD--LIHFLSRQSAWNNNHEAGIE-------SKRNGFFFGKRNGFFFGKRDTEAVNSDLCTSCGPHSAGQC
Op_resi     LK-QQ--VENFANIRGN----DANDNLALQTYLSKHPAR-NNNQEGLD--------KRNGFFYGKRNGFFFGKRDTEAVESNSCIKCGPNNSGQC
Op_savi     LKQQQ--VENFANKYGN----DESDNLALQTYLSKLLAR-NNHIEGLE--------KKNGFFYGKRNGFFFGKRDTEAVESNSCIKCGPNNSGHC
Op_abys     LK-QE--VENFADVNGN----DASDNLAVQTYLSRHPAW-NNYQEGLD--------KRNGFFYGKRNGFFFGKRDTEAVDYNSCIKCGPNNSGQC
Op_angu     ---QP--VEHFTDGN------NAIDNLRLPAYLSRHPSR-NHFEEALD--------KRNGFFYGKRNGFFFGKRDTEAVESKSCIRCGPDDSGRC
Op_caes     LT-QP--VEHFTDGN------NAIDNLRLPTYLPRHPSR-NQYEEALD--------KRNGFFYGKRNGFFFGKRDTEAVEQKSCIRCGPNDSGRC
Op_reti     L--EQ--VENVA-GQGNDN--DARDKLALYNYLSRQSAR-NIYQEGLD--------KRNGFFFGKRNGFFFGKRDTEAVKSDLCVPCGPQNSGQC
Op_lame     L--EQ--VENIA-GQGNNN--DARDKLALYNYLSRQSAQ-NIYPEGLD--------KRNGFFFGKRNGFFFGKRDTEAVESDSCVHCGPQNSGQC
Op_impr     L--EQ--VENVA-GQANDN--DARDKLALYNYLSRQPAR-NIYQEGLD--------KRNGFFFGKRNGFFFGKRDNEAVKSDSCVSCGPQSSGQC
Op_vall     L--QH--VGSVA-GVDNQN--DAKDKLALYNYLSRQPAR-NIYQEGLD--------KRNGFFFGKRNGFFYGKRDTETVKSDSCISCGPQNSGQC
Op_brev     L--EH--TESVA-GLGNTK--EARDKLALYNYLSRQPSR-NIYQEGLD--------KRNGFFFGKRNGFFFGKRDTEAVKSNYCVSCGPEKSGRC
Ba_hero     L--EL--VGNVA-GLESAN--DAREKLALYNYLSRQPAR-NIYQEGLD--------KRNGFFFGKRNGFFFGKRGTAAVESDSCVSCGPDNSGQC
Op_wend     L--EQ--VGNVA-GLESAN--DAKEQLALYNYLAKQSAR-NIYQEGLD--------KRNGFFFGKRNGFFFGKRDNEAVESDSCVSCGPQNSGKC
Cl_cana     --------------------------MALYNYLSRQPAR-NIYQEGLD--------KRNGFFFGKRNGFFFGKRDTEAVESHSCVSCGPQNSGQC
Op_exim_1   ------------------------------NYLSRQPAR-NIYQEGLD--------KRNGFFFGKRNGFFFGKRDTEAVESHSCVSCGPQNSGQC
Op_liod     --------------------------MALYNYLSRQPAR-NIYQEGLD--------KRNGFFFGKRNGFFFGKRDAKAAESDMCVRCGPRNSGQ-
As_tubi     L--EQVPVGNLQ-GYGSEE--DAREHLDLYNFMSRNPAR-SVYRDESH--------KRNGFFFGKRNGFFFGKRDVEAVSSDSCVSCGPENDGQC
As_bidw     L--EQVPVGNLQ-GYGSEE--DAREHLDLYNFMSRNPAR-SVYRDESHKRNGFFFGKRNGFFFGKRNGFFFGKRDVEAVSSDSCVSCGPENDGQC
As_bidw     L--EQVPVGNLQ-GYGSEE--DAREHLDLYNFMSRNPAR-SVYRDESH--------KRNGFFFGKRNGFFFGKRDVEAVSSDSCVSCGPENDGQC
Op_oedi     L--EQVPVGNLQ-GYGSED--DAREHLDLYNFMSRNPAR-SVYRDESH--------KRNGFFYGKRNGFFYGKRDVEAVSSDSCVSCGPENDGQC
Op_oedi     L--EQVPVGNLQ-GYGSED--DAREHLDLYNFMSRNPAR-SVYRDESH----------------KRNGFFYGKRDVEAVSSDSCVSCGPENDGQC
As_love     L--EQ--VVNVQ-GFGSDN--DAREQLDLYKFMSRNPAR-SIYRDESH--------KRNGFFFGKRNGFFFGKRDVEAVTSDSCVRCGPENDGQC
```

```
Am_cipu     VMFGTCCSPQFGCYFMTQEASACTTHHI--ANTCWKQD--LMPSC--GRR--GICVADAVCCSPKDGACRIDLSCSMQEQTNIEYKN
Am_cons_1   VMYGTCCGRQFGCYMMTPEASACITHHI--ANTCQKQD--IMPSC--GGR--GICVSDAMCCSPKDGACRIDLSCSKQQQSNIEYKN
Am_cons_2   VMYGTCCGRQFGCYMMTPEASACITHHI--ANTCQKQD--IMPSC--GGR--GICVSDAMCCSPKDGACRIDLSCSKQQQSNIEYTK
Op_filo     VMFGTCCSPQFGCYFMTPEASPCTQRHI--NNSCWRPD--LMPSC--GRR--GVCVADALCCSPNDGVFRIDFS-------------
Mi_grac     VMFGTCCSSKYGCYFMTPEASPCTQHHI--KNTCWRRD--LMTSC--GRR--GICVAEALCCSPQDGVCRIDLSCLTPQQNNIDNEM
Am_squa     VMYGTCCIPQYGCYIMTPEASSCTQHHV--DSSCWRQD--TIRSCGIGRR--GICAGEALCFLPKDGAFRVDLSCSTTQQKNINDKD
Op_resi     VMFGTCCSAEFGCYFMTKESEACMTPRV---DTCFSQE--KRPTC--SRR--GICVANGICCSPKDGACKIDAVCRSNAKKTTN---
Op_savi     VMFGTCCSPEFGCYFMNKESEACMTPRV---NTCVSEE--KRPPC--SGR--GICVAPGICCSPRDGACKIDAVCRSNAKKTN----
Op_abys     VMFGTCCSPELGCYFMTKESAACMTSRV---DTCLSQE--RRESC--SRR--GICVADGVCCSPRDGACKIDASCSSNAKKTTE---
Op_angu     VGYGMCCSEENGCYIKTKEAAVCRTPDV---GACLNEE--KPQQC--GVG--GICVADGVCCSPRDGACKPEPKCNIHTKQATE---
Op_caes     IGYGMCCSEEHGCYMMTKEAAVCRTSAV---STCVSDE--RAKRC--GIR--GICVADGVCCSPRDGACKPEPKCSILTEKATE---
Op_reti     VMYGTCCSPKFGCYMMTKESEACMSHHA--NNGCWNQE-LMTTSC--GR---GLCVAEGVCCSPRDGACKIDLSCST----------
Op_lame     VMFGTCCSPQFGCYLMTKESEACMSHHV--GNACWDQE-LMTTSC--GRRGVGLCVAEAVCCSPRDGACKIDLSCST-QAKTLGYNE
Op_impr     VMFGTCCSPQFGCYLMTKESEACMTHHV--GTECWNQD--LMPSC--GRR--GICVAEAVCCSPRDGACKIDYSCST-QEKTIDYTE
Op_vall     VMYGTCCSPQYGCYIMTSESEACMRQNLITHSGCWNHE--STPTC--GSR--GICVTDAVCCSPQDGACIIDLTCST-QKKTIN---
Op_brev     VMYGTCCSPQFGCYIMTSEADACKTRHVGTHSDCWKQE--STPSC--GRR--GMCVSESFCCSPEDRACFIDSSCSL---KTLD---
Ba_hero     VMFGTCCSGKLGCYLMTTESEACTKHHV--GTGCWNQE--LMQPC--GRR--AMCVAEGVCCSPQDGACKIDFSCSAAQKKTIE---
Op_wend     VMFGTCCSPEFGCFLMTQESEPCMKHHV--GTGCFDEG--LTPSC--GRRGLGLCVAKDLCCSPQDGACRIDPSCLA-PKKIND---
Cl_cana     VMFGTCCSLQFGCYEMTEESKACMTHHV--GTGCWDLDHELPPTCD-GRR--GVCVAEAVCCSPQDGACKIDLSCSA-QTKTID---
Op_exim_1   VMFGTCCSLQFGCYVMTEESKACMTHHV--STGCWDLA-------------------------------------------------
Op_liod     ---------------------------------------------------------------------------------------
As_tubi     VMYGTCCSPKFGCYLMTKESEACMSNHV--GRECWDQE--SSPSC--GRG--GLCVTNSICCSPKASSCHIDVSCST-QTIPLD---
As_bidw     VMYGTCCSPKFGCYLMTKESEACMTNHV--GRECWDQE--SSPSC--GRG--GLCVTNSICCSPKASSCHIDVSCST-QTIPLD---
As_bidw     VMYGTCCSPKFGCYLMTKESEACMTNHV--GRECWDQE--SSPSC--GRG--GLCVTNSICCSPKASSCHIDVSCST-QTIPLD---
Op_oedi     VMYGTCCSPKFGCYLMTKESEACMTNHV--GRECWDYE--SSPSC--GRG--GLCVANSICCSPKASSCHIDVSCST-QTIPLD---
Op_oedi     VMYGTCCSPKFGCYLMTKESEACMTNHV--GRECWDYE--SSPSC--GRG--GLCVANSICCSPKASSCHIDVSCST-QTIPLD---
As_love     VTYGTCCSPKFGCFLMTKESEACLTNHV--GRECWNHE--SSPLC--GRG--GVCVTNNVCCSPQAGSCHIDVSCST-QTTTTE----
```

NP18

```
Op_filo     MQSSNIAALATFFVFAAVFAQAAYSLSYDAPQRSKRLFW-VDKK-A-DASN--PDKRLFWVDKKTPEKRLFWVDKKSP---------------EK
Am_squa     MQSSNIAALATLCVFVAVFAQAAYSLSYDAPQRSKRLFW-VDKK-S-DASN--PDKRLFWVDKKTPEKRLFWVDKKSP---------------EK
Op_resi     MQSSNIAALATVFVFVAVFAQAAYSLSYNPAERSKRLFW-VDKKSADENSP--IDKRLFWVDKKSPEKRLFWVDKKSPEKRLFWVDKKASPDLEK
Op_savi     MQSSNIAALATVFVFVAVFAQAAYSLSYNPAERSKRLFW-VDKKSADENSP--IDKRLFWVDKKSPEKRLFWVDKKSS------------PELEK
Op_abys     MQSSNIAALATVFVFAAVFAQAAYSLSYNPAERSKRLFW-VDKKSA-DADT--VDKRLFWVDKKSPEKRLFWVDKKSPEKRLFWVDKKS-ADLEK
Op_angu     MQSSNIIALATLFVFVAVFAQAAYSLSYNPAERSKRLFW-VDKKSA--DSP--IDKRLFWVDKKSPEKRLFWVDKKSP---------------EK
Op_caes     MQSSNIIALATLFVFVAVFAQAAYSLSYNPAERSKKLFW-VDKKSA--DSP--IDKRLFWVDKKSPEKRLFWVDKKSPEKRLFWVDKKSGDELEK
Op_fasc     MQSSNIAALATIFVFAAVFAQAAYSLSYDAPQRSKRLFW-VDKK-A-ENSQ--PDKRLFWVDKKTPEKRLFWVDKKTP---------------EK
Op_scha     MQSSNIAALATIFVFAAVFAQAAYSLSYDAPQRSKRLFW-VDKK-A-ENSQ--PDKRLFWVDKKTPEKRLFWVDKKTP---------------EK
Op_reti     MQSSNIAALATIFVFAAVFAQAAYSLSYDAPQRSKRLFW-VDKK-A-ENSQ--PDKRLFWVDKKTPEKRLFWVDKKTP---------------EK
Op_lame     MQSSNIAALATIFVFAAVFAQAAYSLSYDAPQRSKRLFW-VDKK-A-EDSQ--PDKRLFWVDKKTPEKRLFWVDKKTP---------------EK
Op_bisp     MQSSNIAALATLFVFAAVFAQAAYSLSFDAPQRSKRLFW-VDKK-A-ENSQ--PDKRLFWVDKKTPEKRLFWVDKKTP---------------EK
Op_vall     MQSSNIAVLATIFVFAAVFAQAAYSLSYDAPQRTKRLFW-VDKKAA-DNSV--PDKRLFWVDKKTPEKRLFWVDKKSP---------------EK
Op_brev     MQSSNIAALATIFVFVAVFAQAAYSLSYDAPQRSKRLFW-VDKKAA-DNSS--PDKRLFWVDKKTPEKRLFWVDKKTP---------------EK
Ba_hero     MQSSNIAAMATICVFAAVFAQAAYSLSYDTQQRSKRLFW-VDKK-ADEDSL--PDKRLFWVDKKTPEKRLFWVDKKTP---------------EK
Op_appr     MQSSNIAALATIFVFAAVFAQAAYSLSYDTQQRSKRLFW-VDKK-SDEDSL--PDKRLFWVDKKTPEKRLFWVDKKTP---------------EK
Op_vivi     MQSSSIAALATIFVFAAVFAQAAYSLSYDTQQRAKRLFW-VDKK-S-EDSL--PDKRLFWVDKKTPEKRLFWVDKKTP---------------EK
Op_aust     MQSSNIAALATIFVFAAVFAQAAYSLSYDTQQRGKRLFW-VDKK-S-EDSL--PDKRLFWVDKKTPEKRLFWVDKKTP---------------EK
Op_cyli     MQSSNIAALATIFVFAAVFAQAAYSLSYDTQQRSKRLFW-VDKK-A-EDSL--PDKRLFWVDKKTPEKRLFWVDKKTP---------------EK
Op_wend     MQSSNIAALATIFVFAAVFAQAAYSLSYDTQQRSKRLFW-VDKK-A-EDSLPSPDKRLFWVDKKTPEKRLFWVDKKTP---------------EK
Op_plic     MQSSNIAVVATLFVFAAVFAQAAYSLTYNA-DRSKRLFW-VDKK-A-ENSS--PDKRLFWVDKKTPEKRLFWVDKKTP---------------EK
Op_fune     MQSSNIAVLATLFVFAAVFAQAAYSLTYDA-DRSKRLFW-VDKK-A-DNSS--PDKRLFWVDKKTPEKRLFWVDKKTP---------------EK
Op_perf     MQSSNIAVLATLFVFAAIFAQAAYSLTYDA-DRSKRLFW-VDKK-A-ENNV--PDKRLFWVDKKTPEKRLFWVDKKTP---------------EK
Cl_cana     MQSSNIAVLATLFVFAAIFAQAAYSLSYDT-ERSKRLFW-VDKK-A-ENAV--PDKRLFWVDKKTPEKRLFWVDKKTP---------------EK
Op_exim_1   MQSSNIAALATLFVFAAIFAQAAHSLSYDA-DRSKRLFW-VDKK-A-DNSV--PDKRLFWVDKKTPEKRLFWVDKKTP---------------EK
Op_prol     MQSSNIAALATIFVFAAVFAQAAYSLSYNTQERAKRLFW-VDKKAA-ENSL--PDKRLFWVDKKTPEKRLFWVDKKTP---------------EK
Gl_sp_no    MQSSNIAALATLFVFAAVFAQAAYSLSYESPQRTKRLFW-VDKK-A-ETDQ--PEKRLFWVDKKTPEKRLFWVDKKTP---------------EK
Am_laud     MQSSNIAVLATLFVFAAVFAQAAYSLSYETPQRTKRLFW-VDKK-A-ENDQ--PEKRLFWVDKKTPEKRLFWVDKKTP---------------EK
Op_john     MQSSNIAALATLFVFAAIFAQAAYSLSFDAPQRSKRLFW-VDKK-A-ENSQ--PDKRLFWVDKKSPEKRLFWVDKKSP---------------EK
```

```
Op_filo     RLFWVDKKDAKDECVPISNIDKVADCMISVVSQYAKHIEAECKSSGTLS-ESCVNDMNVKRTEANINCLYDTEGGNN--
Am_squa     RLFWVDKKDGEMEL----DYNKIADCMLQVVEELSKHIESQCRVEGSLS-QSCVTDMSNKRAAANKNCLIDPTGGSS--
Op_resi     RLFWVDKKGADVFPVSLNNIDKIADCMIQVVSQYGKHIEEQCKQDGTLS-QSCVEEMNEKRTQANTNCLT--EEGH---
Op_savi     RLFWVDKKGADVVPVSLHDIDKIADCMIQVVSQYGKHIEEQCKQDGTLS-QSCVEEMNEKRTQANTNCLT--EGGQ---
Op_abys     RLFWVDKKGADVVPVSLNDIDKISDCMIQVVSQYAKHIEETCKQDGSLS-QSCVEGMNGKRTQANFNCLTNYEGGVGGE
Op_angu     RLFWVDKKNADVVPVSIDEIDKIADCIIQVVTQYGKHIEEQCKQEDSLS-KSCVEEMNDKRTQANFNCLTNYA------
Op_caes     RLFWVDKKNADVVPVSIDEIDKIADCIIQVVTQYGKHIEEQCKQEESLS-KSCVEEMNEKRTQANFNCLTNYT------
Op_fasc     RLFWVDKKDSDTECVPVTNIDKVADCMINVVSQYAKHIEAECKEPGVLN-EACVKQVNEKRTQANINCLYDTDGN----
Op_scha     RLFWVDKKDSDTECVPVTNIDKVADCMINVVSQYAKHIEAECKEPGVLN-EACVKQVNEKRTQAN-------------
Op_reti     RLFWVDKKDADTECVPVTNIDKVADCMINVVSQYAKHIEAECKEPGVLN-EACVKQVNEKRTQANINCLYDTDGN----
Op_lame     RLFWVDKKDSDKESVLISNIDKIADCMINVVSQYAKHIESDCKQTGTLS-KSCVEKVNEERTQANINCLYETNEGN---
Op_bisp     RLFWVDKKDADAECVPVSNIDKVADCMINVVSQYAKHIEEECKAPGTLS-ESCVKELNQKRTQANINCLYDAEGGN---
Op_vall     RLFWVDKKDSDTQCVPVSNIDKLADCMISVVSQYAKHIETECKEPGTLS-ESCVKQVNEKRTRANINCLYDTEGGN---
Op_brev     RLFWVDKKDADAECVPVTNIDKVADCMISVVSEYAKHIETECKAPGTLS-ESCVKDVNAKRTQANINCLYDTDGSK---
Ba_hero     RLFWVDKKDEDSVQVQVTHINKIADCMIKVVSEYGKHIQAECKQPGVNT-ETCVEKLNVKRAEANMNCLYDYEGGQP--
Op_appr     RLFWVDKKDDDAVQVQVTHINKIADCMIKVVAEYGKHIQAECKQQGVNT-ETCVEQLNVKRAEANMNCLYDYEGGQP--
Op_vivi     RLFWVDKKDDDAAQVQISHINKIADCMIKVVAEYGKHIQAECKQPGVNT-ETCLVDMNVKRAESNMNCLYDFEGGHA--
Op_aust     RLFWVDKKDDDSAQVQIAHINKIADCMIKVVSEYGKHIQADCKQPGVST-ETCIEEMNVKRAESNMNCLYDFEGGQA--
Op_cyli     RLFWVDKKDDDSVLVQVKHINKIADCMIKVVAEYGKHVQAECKQPGVDA-QTCVEELSIKRAEANMNCMSDIEGGQS--
Op_wend     RLFWVDKKSDETIQVPISHINKVADCMLKVVTEYAKHVKAECEQPGVNA-ESCVEQRNVERAEANINCLYDYEGGQP--
Op_plic     RLFWVDKKDDETVTIELKYINKIADCMIKVVSEYAKHIEAECKQPGVET-QTCVEQLNVKRTEANINCLYDFEGGAP--
Op_fune     RLFWVDKKDDDTVTIEIKYINKIADCMIKVVSEYAKHIEAECKQPGVET-QTCVEELNVKRTEANINCLYNFEGGAP--
Op_perf     RLFWVDKKDDDAVTIEIKYINKIADCMIKVVSEYAKHIEAECKQPGVET-QTCVEQLNVKRTEANINCLYDFEGGAP--
Cl_cana     RLFWVDKKDDESVPVHIKYINKVADCMIKVVSEYAKHIEAECKEPGVET-QTCVEKLNVKRTEANINCLYDFEGGAP--
Op_exim_1   RLFWVDKKADETASILIANINKIADCMIKVVSEYAKHIEAECKQPGVET-QACVEQLNVKRTEANINCLYDYEGGHP--
Op_prol     RLFWVDKKDSDTVPAAAGNINKVADCMIKVVSEYAKHIEDECKEQGRIT-QDCVEQLNVKRAEANINCLYDSGGGNH--
Gl_sp_no    RLFWVDKKAADG----LSNINALADCFVKVFSKYLDHVSA-CKEAGLENVKECMEDVNQKRTIANTNCMHDTEGSSK--
Am_laud     RLFWVDKK-ADG----FSKIDALADCMVKVFSKYMEHVSA-CKDEGIDKVKECMSEINAKRTIANTNCMYDTEGAE---
Op_john     RLFWVDKK-ADAPNFPVSNINDIADCMIKVFAKYAEHVEA-CKNSGVEGLKECMEEVNQKRTISNTNCLSDTEGGM---
```

NP26

```
Am_cipu      MLGMKTLVAYAAIVLALIV-TTGTSEDIE----DTEDLQIADE-NELTYI-----EPN-YQELLISLQKAREN--GA-LQDIDD-----KRMAS-
Mi_grac      MLGMRSLLAYAAIVLALVV-SIGTSEDIE----DTAE-ELLDE-NVITDVD----EPN-YQDLLLSLQKARRN--GL--QDVDD-----KRMAA-
Am_squa      MLGMRSLLAYAAVVLAVIV-STGTSEDLE----NTEE--FVDE-NALTDFE----QPDYQQELLSRVQKAREN--DF--EDVDD-----KRMAA-
Op_savi      MLGMKTLLAYAAIVLALVV-TTGTSEDIE--DPETEELQLADE-NEITDI-----EPD-YEALLASLQRAREI--GYDGDDVDD-----KRMAS-
Op_abys      MLGMKTLLAYAAIVLALVV-TTGTSEDIE--DLDTEELQLGDE-NEITDI-----EPN-YQALLSSLQRAREN--GY--DDVDD-----KRMAS-
Op_angu      MLPLKSLLASVAIVLALIVTTTGTTEDLE--DTDTQNIEIPND-NDIMDI-----EPDDFEALLSNLQKAREN-LYE--DDIDA-----KRMAS-
Op_caes      MLPMRTLIASAAIVLTLIVTTTGTTEDIE--DTETHNLEIPDD-NDIMDI-----EPD-FEALLSSLQKARDN-LYE--DDIDD-----KRMAS-
Op_reti      MLGMKTLLAYAAIVLALVV-TTGTSEDIE--EPDTQELQLADE-NELMDI-----EPD-YPELLMNLQKAREN-GGL--QDVDD-----KRMAS-
Op_lame      MLGMKTLLAYAAIVLALVV-TTGTSEDIE--EPETQELQLADE-NELMDI-----EPD-YQELLMNLQRAREN--GL--QEVDD-----KRMAS-
Op_impr      MLGMKTLLAYAAVVFALVV-STGTSEDIE--EPEIEELQLADE-NELMDI-----EPD-YQELLLNLQKAREN--GV--QDIED-----KRMAS-
Op_bisp      MLGMKTLLAYAAIVLALVV-SIATSEDIE--EPDSEELQLADE-NELMDI-----EPD-YQELLMNLQKAREN--GV--QDIDY-----KRMAA-
Op_brev      MLGVKTLVAYAAILLALIV-STGTSEDID--EPDTQQLQLLDE-NELMDI-----EPD-YEELLMNLQRAREN--G---KEFDE-----KRMSS-
Ba_hero      MLGVKAVLAYAAIVLALVV-SIGTSEDVI--DPETEDLELADE-NELMDL-----ETD-YQELLLNLQRARAN-AGE--QELDE-----KRMSS-
Op_cyli      MLGVKTVLAYAAIVLALVV-STGTSEDVT--EPETEELELADQ-NELMDL-----EPD-YQELLLNLQRARANAAGE--QEVDE-----KRMSS-
Op_wend      MLGMKTILAYAAIVLALVV-TTGTSEDVI--EPETEELELGNE-NEFVDL-----EPD-YQELLLNLQRAREQ-AGE--HELND-----KRMASS
Op_plic      MLGVKTILAYAAVVLALVV-STGTSEDIT--EPEAEELELANE-NELMDL-----EPE-YQELLMNLQRARSN-AGD--QEIDE-----KRMSAS
Op_fune      MLGVKTILAYAAIVLALVV-STGTSEDIT--EPEAEELELANE-NELMDL-----EPD-YQELLLNLQRARSN-AGD--QEIDE-----KRMSAS
Op_perf      MLGVKTILAYAAVVLALVV-STGTSEDIT--EPEAEELEL-NE-NELLDL-----EPD-YQELLMSLQRARAN-AGD--QEIDE-----KRMSAS
Cl_cana      MLGVKTILAYAAVVLALVV-STGTSEDIT--EPEAEELELANE-NELMDL-----EPD-YQELLMNLQRARSN-AAE--QELDE-----KRMAAS
Op_exim_1    MLGVKTILAYAAVVLALVV-STGTSEDIT--EPESEEFELANE-NELMDL-----EPD-YQELLMNLQRARSN-AGD--QELDE-----KRMSAS
Op_exim_2    MLGVKTILAYAAVVLALVV-STGTSEDIT--EPESEELELANE-NELMDL-----EPD-YQELLMNLQRARSN-AGD--QELDE-----KRMSAS
Op_liod      MLGMKTVLAYGAIVLALVV-STGTSEDIV--EPESEELELANQ-NEFMDL-----EPD-VQELLMNLQRARAS--GE--QEIDD-----KRMTSA
Op_prol      MLGMKTVLAYAAIVLALVV-STGTSEDIV--EPEEDELELANE-NDLMDL-----EPD-YQELLMNLQKARAN--GE--LEVDD-----KRMASS
As_tubi      MLGVKTVLAYFAIVLALVV-TAGTSEDVD--ESDTEKYDQSDD-NNVIDFDFKDSEPD-YQELLMNLQNARAI--EL---EADD-----KRARGA
Op_oedi      MLGVKTVLAYSAIVLALVV-TAGTSEDVD--ESDTEKYDQSDD-NNVIDFDFKDSEPD-YQELLMNLQNARAT--EL--EEADD-----KRARGA
As_love      MLRVKTVLAYSALVLALVV-SAGTSEDVE--ESDAEKYDQVDD-NNFIDFDFKDIEPD-YQELLLNLQNARAK--QL---EFDDMSSRWKKAQEA
Gl_sp_no     MLGVKSMLAYAAIVLALVV-STGTSEDI---EPETDEYGLADDSDDVIDF--KDIEPD-YQELLLNLQRARAN--SA--QDFNN-----KRMASS
Op_john      MLGVKTVLAYAAIVLALVV-STGTSEDVRPIEPEADEFELADD-NDLIDF--KDIEPE---ELLMNLQRARAI--GQ--QELDE-----KRMSS-
```

```
Am_cipu    -GWKRSGM-AAGWKRGQNAAAGWKR---------------------------------------------------GSN---AAAGWKR-GQALP
Mi_grac    -GWKRADM-AAGWKRGQSAAAGWKRGQSAAAGWKR-----------------------------------------PAN---AAAGWKR-G-AMA
Am_squa    -GWKRGNSAAAGWKRGNSAAAGWKRGQTAAAGWKR-----------------------------------------PANA--AAAGWKRGGTSMA
Op_savi    -GWKRGQSAAAGWKRGQSAAAGWKR---------------------------------------------------GHA---AAPGWKR-GHAAA
Op_abys    -GWKRGQSAAAGWKRGQSAAAGWKR---------------------------------------------------PANPANAAAGWKR-GQAAA
Op_angu    -GWKRGHSAVSGWKRDQP---QPRR-QMAASGWKR-----------------------------------------GNA---AASGWKR-GQAAA
Op_caes    -GWKRGHSAAAGWKRDQS---QPR-GQMAASGWKRDQSQPRGQSAASGWKRDQSQPRGQSAASGWKRPANAAGWKRGHA---AASGWKR-GQAAV
Op_reti    -GWKRGQSAAAGWKRGQSAAAGWKR---------------------------------------------------PA----SAAGWKR-GQAAA
Op_lame    -GWKRGQSAAAGWKRGQSAAAGWKR---------------------------------------------------PA----SAAGWKR-GQAAA
Op_impr    -GWKRGPSAAAGWKRGQSAAAGWKR---------------------------------------------------PAS---AAAGWKR-GQSAA
Op_bisp    -GWKRGQSAAAGWKRGQSAAAGWKR---------------------------------------------------PAS---AAAGWKR-GQSAA
Op_brev    -GWKRAQS-AAGWKRAQSA-AGWKRAPSAAAGWKR-----------------------------------------PAS---AASGWKR-APSAA
Ba_hero    -GWKRAQG-AAGWKRTQSAAAGWKR---------------------------------------------------PAS---AAAGWKR-GQSAA
Op_cyli    -GWKRAQS-AAGWKRTQSAAAGWKR---------------------------------------------------PAS---AAAGWKR-GQSAA
Op_wend    -GWKRAQG-AAGWKRTQNAAAGWKR---------------------------------------------------PAS---AAAGWKR-GQSAA
Op_plic    -GWKRAQ--AAGWKRAQSAA-GWKR---------------------------------------------------PAS---AAAGWKR-GQPAA
Op_fune    -GWKRAQ--AAGWKRAQSAA-GWKR---------------------------------------------------PAS---AAAGWKR-GQAAA
Op_perf    AGWKRAQG-AAGWKRAQSAA-GWKR---------------------------------------------------PAS---AAAGWKRNGQSAA
Cl_cana    -GWKRAQG-AAGWKRMQSAA-GWKR---------------------------------------------------PAS---AAAGWKR-GQAAA
Op_exim_1  -GWKRAQG-AAGWKRTQSAA-GWKR---------------------------------------------------PAS---AAAGWKR-GQAAA
Op_exim_2  -GWKRAQG-AAGWKRTQSAA-GWKR---------------------------------------------------PAS---AAAGWKR-GQAAA
Op_liod    -GWKRAQG-AAGWKRAQGAA-GWKR---------------------------------------------------PAS---AAAGWKR-GQSAA
Op_prol    -GWKRAQG-AAGWKRGQSAA-GWKR---------------------------------------------------PAS---AAAGWKR-GQSAA
As_tubi    AGWKRGQT-AAGWKRQPHAAAGWKR---------------------------------------------------GQT---AAAGWKR-RQTAA
Op_oedi    AGWKRGQT-AAGWKRQPHAAAGWKR---------------------------------------------------GQT---AAAGWKR-RQTAA
As_love    AGWKRAQA-AAGWKRQPSAAAGWKR---------------------------------------------------GQS---AAAGWKR-EQSAA
Gl_sp_no   -GWKRAQG-AAGWKRAQGAA-GWKR---------------------------------------------------SQ----GASGWKR-AQG-A
Op_john    -GWKRAQG-AAGWKRGQSAA-GWKR---------------------------------------------------APN---AAAGWKR-GQS-A
```

```
Am_cipu     GGWKRGQSAAASGWKRGHA-AAGWKR---------DFEVDPIDVDTRGGETWRNNQVK-TASGWKRTSRSANLLQQ--NDEDEP
Mi_grac     GGWKRGQAAAA-GWKRGPAAAAGWKR---------DFE--PIEVDTRGDGNWRDNQIK-TASGWKRTSRSVNNVQE-NAEEEEP
Am_squa     GGWKRGQAAAA-GWKRGNSAAAGWKR---------DFE--PIEVDTRDNGNYRNNQIK-AASGWKRTSRSVNLVQD-NTEEEEP
Op_savi     PGWKRGHAAAP-GWKRGQYAAAGWKR-AQAAGWKRDY---PIEVDTRAGENWRNSQMK-AASGWKRT---------------LH
Op_abys     AGWKRGQAAAA-GWKRGQSAAAGWKRAAQAAGWKRDY---PIDVETRG-ENRRDPQMK-AASGWKRT---------------LH
Op_angu     SGWKRGQAAA-SGWKRGQAAASGWKR---------D----PIEVDTRGNNNWRNSQVK-SASGWKRTVY---------------
Op_caes     SGWKRGQAAA-SGWKR-------------------D----PIEVDARGDNNWRNSQIK-SASGWKRTPY---------------
Op_reti     AGWKRGQSAAA-GWKRGQSAAAGWKR---------DYN--PIEVDTRGGENWRKANMNAAASGWKRTARSANRQQ---NANQLP
Op_lame     AGWKRGQSAAA-GWKR-------------------DYN--PIEVDTRGGENWRNANMKAAASGWKRTARSASRQQNVENANQLP
Op_impr     AGWKRGQSAAA-GWKRAK--PAGWKR---------DY---PIEVDTRGGENWRNGNMKSASSGWKRTARSATRQQNVENANQLP
Op_bisp     AGWKRGQSAAA-GWKRAK--AAGWKR---------DY---PIEVDTRAGENWRNGNVKSASSGWKRTARSATRQQYVENANQLP
Op_brev     AGWKRAQGAA--GWKR-------------------DY---PIDIDTRGDENWKNANMKNAASGWKRTSRSASR--NVDNIDQLP
Ba_hero     AGWKRAKG--ASGWKR-------------------DN---PIDVDTRGGENWRNSNLKTASSGWKRTARSANRQQRVENTNQLP
Op_cyli     AGWKRAKG--ASGWKR-------------------DN---PIDVDTRGGENWRNTNLKTASSGWKRTARSANRQQRVD---QLP
Op_wend     AGWKRAQSAA--GWKR-------------------DN---PIDVDTRGGDNWRNNNLK-SASGWKRTARSATRQQRVDNSNQLP
Op_plic     AGWKRAKG--ASGWKR-------------------DN---PIDVDTRGGENWRH-NEKTASAGWKRTARSATRQQRVDNTYQFP
Op_fune     AGWKRAKSAA--GWKR-------------------DN---PIDLDTRGGENWRHNDKTSASSGWKRTARSATRQQRADNTYQLP
Op_perf     AGWKRAKG--ASGWKR-------------------DN---PIDVDTRGGENWRH-NEKTASSGWKRTARSAPRQQRVDNTYQLP
Cl_cana     AGWKRAKG--ASGWKR-------------------DN---PIDVDTRGGENWRNSNEKTASSGWKRTARSATRQQRVDNTYQLP
Op_exim_1   AGWKRAKG--ASGWKR-------------------DN---PIDVDTRGGENWRNTNEKTASSGWK-------------------
Op_exim_2   AGWKRAKG--ASGWKR-------------------DN---PIDVDTRGGENWRNTNEKTASSGWKRTARSATRQQRADNTYQLP
Op_liod     AGWKRAKG--ASGWKR-------------------DN---PIDMETRGGENWKNPNLKTASAGWKRTARSANIQQRVDNTNQLP
Op_prol     AGWKRAKG--ASGWKR-------------------DN---PIDLDTRGGENWRNPNLKTASSGWKRTPRSASRQQRVDNTNQLP
As_tubi     AGWKRAKGAA--GWKR-------------------DN---QIDLDTRGGNSWRNSNLK-AAAGWKRTSRSANRQQEEDTIDQIP
Op_oedi     AGWKRAKGAA--GWKR-------------------DN---QIDLNTRGGNSWRNSNLK-AASGWKRTSRSANRQQEEDTIDQIP
As_love     AGWKRAKG--ASGWKR-------------------DN---PIDLNTRGGENWRNSNSKSAASGWKRTTRSANRQQIENTINQLP
Gl_sp_no    AGWKRAKS--ASGWKR-------------------DN---PISMETRGDENWRHNNIKSASSGWKRTSRSANKEQRIEDVNQLP
Op_john     AGWKRGKS--ASGWKR-------------------DS---PISLETRSGENWRNANLKTASSGWKRTARSANRQQTV--VNQHP
```

NP27

```
Am_cipu      MRMITC-TALLCVLLAL-LDTRQAVC--EAG-IPVRYQTGTFFGKRT-VTDEDALRALDGPYGRNLLDLLKAWVYRYSQMEAAEQVDAK
Am_cons_1    MRMITC-TALLCVLLAL-LDTRQAVC--EGG-IPVRYQTGTFFGKRT-VTDEDALRALDGPYGRNLLDLLKAWVYRYNQMEAAEQLDSK
Op_filo      MRLVTCATALLCVLFAL-LDTRQAVC--EAG-IPVRYQTGTFFGKRQ-VSDEDVLRALDGPYGRNLLDL-------------------
Mi_grac      MRLITCATALICVLLAL-LDTRQAVC--EAG-IPVRYQTGTFFGKRQ-VSDEDVLRALDGPYGRNLMDLLKTWVYRYSQMEAADQLETN
Am_squa      MRLIACTTALICVLLAL-LDTRQAVC--EGG-IPVRYQTGTFFGKRQ-VSDADVLRALNGPYGRNWMDLLKEA-YRYSQLEAADQLEGN
Op_resi      MRMITC-AALLCVLLAL-LDTRQNGC--EAG-IPVRYQTGTFFGKRQVTSDEDVLRALAGPSNRNLLNLMKAW-YQLNQLQGVDQVESN
Op_savi      MRMITC-AALFCVLIAL-LDTRQNGC--EAG-IPVRYQTGTFFGKRQVTSDEDVLRALAGPSDRNLLNLMKAW-YQLNQLQGVDQVESN
Op_abys      MRMITC-AALFCVLLALILDTRQNGC--EAG-IPVRYQTGTFFGKRNVISDEDVLRALEGPYGRKLLDLMKAW-YQLNQLEAVEQVESN
Op_angu      MRMITC-AAVLCLLLALILDTRQNGC--EAN-IPVRYQTGTFFGKRKAATDEEIMRSLEGAHGRNVLDLVKTW-YQLNQLQGVEQLDSN
Op_scha      --------------LAL-IDTRQNGC--EAG-IPVRYQTGTFFGKRQ-VSDEDVLRALDGPYGRNLLDLMKAWVYRLHQMEAAEQVESN
Op_reti      MRMLTC-AALLCVLLAL-IDTRQNGC--EAG-IPVRYQTGTFFGKRQ-VSDEDVLRALDGPYGRNLLDLMKAWVYRLHQMEATEQVESN
Op_impr      MRMITC-TALLCVLLAL-LDTRQSGC--EAG-IPVRYQTGTFFGKRQ-ISDEDVLRALDGPYGRNLLDLMKARVYR--QMEAAEEVESN
Op_bisp      MRMITC-AALLCVLLAL-LDTRQSGC--EAG-IPVRYQTGTFFGKRQ-VSDEDVMRALDGPYGRNLLDLMKAWVYRLNQMEVAEQVESN
Op_vall      MRMITC-AALVCVLLAL-LDTRQNGC--EAG-IPVRYQTGTFFGKRQ-VSDEDVMRTLDGPYGRNLLDLMKAWVYRLNQMETADQIEAN
Op_brev      MRVITC-AALLCMLLAL-LDTRQSGC--EAG-IPVRYQTGTFFGKRQ-VSDDDVLRALDGPYGRNLLDLMKAWVYRLNQLEVGEQVEAN
Ba_hero      MRMLTC-AALLCILLAL-LDTRQSGC--EAG-IPVRYQTGTFFGKRQ-MSDEDVLRALDGPYGRNLLDLMKAWVYRLKQMEAAEEVESN
Op_wend      MRMLTC-AALLCVLLAL-LDTRQSGCVDAAG-IPVRYQTGTFFGKRQ-MSDEDVLRALDGPYGRNLLDLMKAWVYRLKQMEAVDEVEAH
Op_fune      MRVVTC-AALFCVLLAL-LDTRQNGC--EAG-IPVRYQTGTFFGKRQ-TSDEDVMRALDGPYGRNLLDLMKAWLYRLNQIEAAQEVESN
Op_perf      MRMITC-AALFCVLLAL-LDTRQNGC--EAG-IPVRYQTGTFFGKRQ-TSDEDVMRALDGPYGRNLLDLMKAWLYRLNQMEAAQEVESN
Cl_cana      MRMITC-AALFCVLLAL-LDTRQNGC--EAG-IPVRYQTGTFFGKRQ-TTDEDVMRALDGPYGRNLLDLMKAWLYRLNQMEAAQEVESN
Op_liod      MRMITC-AALLCVLLALILDTRQSGC--EAG-IPVRYQTGTFFGKRQ-MSDEDILRALNGPYGRNMMDRMKSWVYR--QMEAAEEVESN
Op_prol      MRMITC-AALLCVLLAL-LDTRQNGC--EAG-IPVRYQTGTFFGKRS-MSDEDVLRALDGPYGRNLLDLMKAWVYRLNQMEAAEEVESN
As_tubi      MRMLIF-AALLCVLLAL-LDTRQNGC--EAG-IPVRYQTGTFFGKRQTLTDEDVLRALDGPYGRNLMDLMKSWLYRLNQIKVAKEVESN
As_bidw      MRMLIF-AALLCVLLAL-LDTRQNGC--EAG-IPVRYQTGTFFGKRQTLTDEDVLRALDGPYGRNLMDLMKSWLYRLNQIKVANEVESN
As_love      MRMITC-ATLLCVLLAM-LDTRQNGC--EAG-IPVRYQTGTFFGKRQTMTDEDVLRALDGPFGRNLMDLMKSWLYRLNQLQVTKEVESN
Gl_sp_no     MRVITC-TVLMCVLLAL-IDTRQSYC--EAGRIPVRYQTGQIFGKRQ-MSDEDVLRALDGPYGRNLLDLMKLSVYRLNQLEGTDEVESK
Op_john      MRVITC-ATLLCVLLAL-IDTRQSGC--EAGRIPVRYQTGQIFGKRQ-MTDEDVLKVLDGPYGRNLLDLMKLSAYRLNQLEGADDVESN
Op_lyma      MRVITC-AALLCVLLAL-IDTRQSGC--EAGRIPVRYQTGQIFGKRQ-MTDDDVLRALEGPYGRNLLDLMK-----------------
```

PDF

```
Am_cipu     MHATLLFSSAIVVVLLGLAASSSDDLPDKRIADNDFAQMRSQADRDFEVVAFKNLLKEYLRTYGKRD--VEKRLSQNDFSQLRSNQLDEELTKQLIARFLHQAGRR
Am_cons_1   MHATLLFSSAILVVLLGLVA-SSDDLPDKRIADNDFAQMRSQADRDFEVVAFKNLLKEYLRTYGKRD--VEKRLSQNDFSQLRSNQLDEELTKQLIAKFLHQAGRR
Mi_grac     MHATLLFSSAILVVLLGLAASSSDDLPDKRIADNDFAQMRSQADRDFEVVAFKNLLKEYLSKYGKRN--VEKRLSQNDFSQLRSNLLDEELTKQLIAKFLEQAGRR
Am_squa     MHATLLFSSAILVVLLGLAASSSDNLPDKRIADNDFAQMRSQADRDFEVVAFKNLLKEYLRTYGKRD--VEKRLSQNDFSQLRSNQLDEELTKQLIAKFLQQAGRR
Op_savi     MHTTLIFSSAVLAVLLGVVI-STDSLPDKRIADNDFAQMRSQADRDFEVIAFKNLLREYLSNYGKRN--VEKRLSQNDFAQQRSNQMEEDIMKQMIAQILKNAGRR
Op_abys     MHTTLIFSSAILAVLLGLVA-SSDSLPDKRIADNDFAQMRSQADRDFEVIAFKNLLREYLGKYGKRD--VEKRLSQNDFAEQRSNQIDEEIMKQMIAQFLNRAGRR
Op_caes     MHTTLMFSSAIAVVLLALTV-STESLPDKRIADNDFAQMRSQADRDFEVLAFKNLLREYLGKHGKRN--VEKRLSQNDFAEQRSNQNDEEIMKQMIAQFLQKTGRR
Op_scha     MHTTLIFSSAILAVLLGLAA-SSDSLPDKRIADNDFAQMRSQADRDFEVIAFKNLLREYLGRQGKRD--VEKRLSQNDFSQMRSNLLDEELTKQLIAKFLYQAGRR
Op_reti     MHTTLIFSSAILAVLLGLAA-SSDSLPDKRIADNDFAQMRSQADRDFEVIAFKNLLREYLGRQGKR --VEKRLSQNDFSQIRSNLLDEELTKQLIAKFLYQAGRR
Op_impr     MHTTLIFSSAILAVLLGLAA-SSDSLPDKRIADNDFAQMRSQADRDFEVIAFKNLLREYLGRQGKRD--VEKRLSQNDFSQLRSNLLDEELTKQLIAKFLYQAGRR
Op_brev     MHTSLIFSSAILAVLLCLVASTSDLLPDKRIADNDFAAMRSQADRAHENILFKNLLKQILFEQGKRD--VDKRLSQNDFSQLRSNLLDEELTKQLIAKFLYQAGRR
Ba_hero     MHTTLIFSSAILAVLLCLAASSSDLLTEKRIADNDFAQMRSAADRANEDIKFRNILKQILFEQGKRD--VDKRLSQNDFSQLRSNLLDEELTKQLIAKFLFQAGRR
Op_wend     MHTTLIFSSAILAVLLGLAASSSDLLTEKRIADNDFAQMRSAADRANEDIKFRNILKQILFEQGKRD--VDKRLSQNDFSQLRSNMLDEELTKQLIAKFLFQAGRR
Op_fune     MHTTIVFLSAILAVLLGLAT-STDLLPEKRIADNDFAQMRSAADRANEAIAFRNLLKQILFEQGKRDV-VDKRLSQNDFSQLRSNQLDEELTRQLIAKFLFQAGRR
Op_perf     MHTTIVFSSAILAVLLGLAA-SSDLIPEKRIADNDFAQMRSAADRANEAIAFRNLLKQILFEQGKRD--VDKRLSQNDYSQLRSNQLDEELTKQLIAKFLFQAGRR
Cl_cana     MHTTLIFSSAILAVLLGLAA-SSDLLPEKRIADNDFAQMRSAADRANEAIAFRNLLKQILFEQGKRD--VDKRLSQNDFSQLRSNQLDEELTKQLIAKFLFQAGRR
As_tubi     MHTSLILSSAILAVLLSLAA-SSDLLPDKRIADNDFAQMRSEADRANEVIAFRNLLQQILKEQGKRSY-IDKRLAQNDFSQLRNNQLDEELTKQLIAKFLSQAGRR
As_bidw     MHTSLILSSAILAVLLSLVA-SSDLLPDKRIADNDFAQMRSEADRANEVIAFRNLLQQILKEQGKRSY-IDKRLAQNDFSQLRNNQLDEELTKQLIAKFLSQAGRR
As_love     MHTSLLFSSAILAVLLSLVA-SSDLLPDKRIADNDFAQMRSEADRANEVIAFRNLLQQILKEQGKRSY-IEKRLAQNDFSQIRNNQLDEELTKQLIAKFLNQAGRR
Op_john     MHITLIFSSAILAVLLCLTA-SSDLLEEKRIADNDFAQMRSIADRKNEAIAFRNLLSQILKEQGKRD--VEKRLSQNDFSQLRSNLLDEELTKQLIARFLYRAGRR
```

F-type SALMFa

```
Am_cipu     MARVRNVVLLFATL-CCYASISS-GD-ENQ-V-GDNEEIDH-EQLVHLV-KRVANQVQFLEDQ-LGM---SD-------NNEEL-VKSLSKRQASN-QPA-NGLPMNVPVKMSGFAFGKRD-G
Am_cten     MARVRNVVLLFATL-CCYASISS-GD-ENQ-V-GDNEEIDH-QQLVHFV-KRVANQVQFLEDQ-LGM---SD-------NNEEL-VKSLSKRQASN-QPA-NGLPMNVPVKMSGFAFGKRD-G
Am_cons_1   MARVRNVVLLFATL-CCYASISS-GD-ENQ-V-REKEEIDH-DQLVDFV-RNLAEQVELLEDHVLSM---SE-------NNEGL-VKSLSKRQASN-RPA-SGLPMNVPVKMSGFAFGKRDGG
Am_cons_2   MARVRNVLLLFATL-CCYASISS-GD-ENQ-V-REKEEIDH-DQLVDFV-RNLAEQVELLEDHVLSM---SE-------NNEGL-VKSLSKRQASN-RPA-SGLPMNVPVKMSGFAFGKRDGG
Op_filo     MARVRNIVLLFATL-CCYASISS-GD-GNN-F-DDNQENNQ-DEFIDLA-RRIEREVQLLNKQ-LGI---TE----Y-NEDENL-VKSLSKRQANK-GSG-SGLPMNVPVKMSGFIFGKRD-A
Mi_grac     MARVRNIVLLFATL-CCYASISS-GD-GND-L-DGNQQNNQ-EKVLEHA-RRIEREVQLLNKE-LGF---TD----Y-NEDEDL-VKSLIKRQANK-GSG-NGLPMNVPVKMSGFSWGKRD-A
Am_squa     MARVRNIVLLFATL-CCYASISS-GD-GNN-L-DEQQ--NQ-KEVLDLAIERIEREVHLLQEE---L---SE----Y-KQDEDLSVKSLHKRQANK-GSGNSGLPMNVPVKMSGFAFGKRD-A
Op_resi     MARVRHVVLLFATL-CCYASISY-AN-ENE-Y-EPSQQLDH-EKLEDLA-SRIGEELRILELE-LGM---QD------SNDVDM-VKSLQKRQAAT-RSGGSGLPMNVPVKMSGFAFGKRD-G
Op_savi     MAMVRHVVLLFATL-CCYASISH-ADEENE-Y-EPSQKLDH-EKLVALA-SRIAEEVRILELG-LGM---QD------SNDVDI-VKSLQKRQAAT-RSGGSGLPMNVPVKMSGFAFGKRD-G
Op_abys     MARVRHVVLLFATL-CCYASISY-AD-ENE-Y-EPSQELDHEEKLVALA-SRIIDEVR--NFE-LGL---KD-------NDADM-VKSLQKRQATT-RS--SGLP--VPVKMSGFAFGKRD-G
Op_angu     MARVRHVVLLFAAL-CCYTSISH-AE-ENE-Y-QTNQELEH-DKLVELA-SRIAKEVRILELG-LGI---QQYNEDDTEDNTDI-IKSLQKRQAATPRSGGSGLPMNVPVKMSGFAFGKRD-G
Op_scha     MARVRNIVLLFATL-CCYASISYAVD-EEE-Y-EGTPELNN-EQLVQLV-KGIADEVRILELE-LGV---QQ-------KDVDM-VRSLSKRQAVN-NPA-NGLPMNVPAKMSGFVFGKRDAS
Op_reti     MARVRNIVLLFATL-CCYASISYGVD-EDE-Y-QGTQD-NT-EQLKQLV-EGIGDEVRILELV-LGG---HP-------NKVNM-VRSLSKRQAVN-NPA-NGLPMNVPAKMSGFVFGKRDTS
Op_lame     MARVRNIVLLFATL-CCYASISHAAD-EDE-Y-EGTQDLNH-EQLVQFA-RDMEHGLWRLEQE-LGM---QR-------DE-DM-VKSLSKRQAV--NPA-NGLPMNVPVKMSGFAFGKRDNS
Op_impr     --------LLFATL-CCYASISY-AD-ENE-F-GATEDIDH-EQLVQLA-RKIAEEVRILELE-LGM---QE-------NDVDI-VKSLSKRQAVN-RPA-NGLPMNVPVKMSGFAFGKRD-G
Op_bisp     MARMRNIVLLFATL-FCYTSISY-AN-EDE-Y-GGNQDLDH-EQLVELA-RRIATEVRILEQE-LGM---QE-------NDVDI-VKSLSKRQAVN-RPANNGLPMNVPVKMSGFAFGKRD-G
Op_vall     MARARNLVLLFATI-CCYASISY-AD-EQE-Y-EGTQDLDH-EQLVELA-RRITEEVRILEMELEDL---QD-------NHVPS-VKSLSKRQAVN------GLPMNVPVKMSGFAFGKRDGS
Op_brev     MARVRNLVLLLATI-CCYASISY-AD-EQE-F-EGSPDIDH-EQLVELA-SRIANEVRILELE-LSR---QD-------NDADI-VKSLSKRQA-------GGLPMNVPVKMSGFAFGKRDGG
Ba_hero     MARVRNIVLLFVTL-CCYTSISH-AN-EP--------DVDQ-ENLVELA-RQIGEEVRILKVG-LGI---HD------NNDV---VKSLSKRQAVN-HPA-SGLPMNVPVKMSGFAFGKRD-S
Op_appr     MARVRNIVLLFVTL-CCYTSISQ-AN-EP--------DVDQ-ENLLDLA-RKIEEEVRILERE-LN------------ENDVDN-VKSLSKRQAVN-NPA-SGLPMNVPVKMSGFAFGKRD-S
Op_macu     MARVRNIVLLFVTL-CCYTSISH-AN-EP--------DVDR-EDLAELA-KKIEDEVRLLEIG-LGI---RD------NNDVDI-VKSLSKRQAAN--PA-SGLPMNVPVKMSGFAFGKRD-S
Op_vivi     MARLRNIVLLFATL-CCYTSISH-AD-EPN-Y-VVNPDVDQ-ENLVELA-RQIGEEVRILEVG-LGI---RD------NDDVDI-VKSLSKRQAGN-NPA-SGLPMNVPVKMSGFAFGKRD-S
Op_cyli     MDRVRNILLLFVSL-CCYTSISH-AN-EPN-Y-GVAENADQ-ENLVELA-RKIGEEVRILELE-LGI---HG------NDDVDI-VKSLSKRQAVN--PA-SGLPMNVPVKMSGFAFGKRD-S
Op_wend     MARVRNLVLLCVTA-CCYASLCH-AN-ESK-Y-GESPDLDQ-ENLVELA-KKIGEQVRLLELG-LRM---PA------DDDVDI-VKSLSKRQAKD--PE-SGLPMNVPVKMSGFAFGKRD-T
Op_plic     MAGVRNLLVLFTTL-CCYASISH-AN-EPK-Y-EGTQDLDQ-ENLVEFA-RKIGDELRILELG-LGIQ--NN------DDNEDI-VKSLSKRQAGN-NPA-GGLPMNVPVKMSGFSFGKRD-A
Op_fune     MAGVRNLLLLFTTL-CCYASISH-AN-ESK-Y-EGTQDLDQ-ENLVEFA-RKIGDELRILELR-LG-----N------DDNEDI-VKSLSKRQA-N-NPA-SGLPMNVPVKMSGFSFGKRD-A
Op_perf     MARVRNLLLLFATL-CCYTSISH-AN-EPK-Y-EATPDLDQ-ENLVELA-RQIGDKLRLLQFG-LGI---EN------NDE----VKSVSKRQAGS-NPG-SGVPMNVPVKMSGFAFGKRD-A
Cl_cana     MAGVRNLLLVFATL-CFYASISH-AN-EPK-Y-EGTQDIDQ-ENLVELV-RKFGHDLRILEEG-LGI---KN------IDDEGS-VKSLSKRQAGN-NPA-SGLPMNVPVKMSGFAFGKRD-A
Op_exim_1   MAGVRNLLLLFATL-CCYASISH-AN-EPK-Y-GGNPDMDQ-DNLVELA-RKIGDDWRLLELG-LGIQHVQN------IDDEDI-VKSLSKRQAGN-NPA-SGLPMNVPVKMSGFTFGKRD-A
Op_exim_2   MAGVRNLLLLFATL-CCYASISH-AN-EPK-Y-GGNPDMDQ-DNLVELA-RKIGDDWRLLELG-LGIQHVQN------IDDEDI-VKSLSKRQAGN-NPA-SGLPMNVPVKMSGFTFGKRD-A
Op_liod     MARVRNIVLLFATL-CCYTSISH-AN-EQG-Y---EEEINK-ENLEDFA-RKLAAEVRILQLE-LGM----E------DNEENI-VKSLSKRQADT-RPG-SALPMNVPVKMSGFAFGKRD-A
Op_prol     MARVRNVVLLFATL-CCYTLISH-AH-AEG-YEATPEEVSK-ENLIEFA-RKIAEEVRILKLG-LGM----------DNDVDI-VKSLSKRQADD-HIG-SGLPMNVPVKMSGFAFGKRD-A
As_tubi     MAQMRNLVVLFVTL-FCYASTSN-AD-DNN-FAANEEELNH-EQLVELA-RRISDEVRILELE-LGF---QD-KN---DDELET-VKSLSKRQAGN-RPG-SGVPMHVPVKMSGFSFGKRD-A
As_bidw     MAQMRNLVVLFVTL-FCYASTSN-AD-DNN-FAANEEELNH-EQLVELA-RRISDEVRILELE-LGF---QD-KN---DDELET-VKSLSKRQAGN-RPG-SGVPMHVPVKMSGFSFGKRD-A
Op_oedi     MAQMRNIVVLFVTL-FCYASTSN-AD-DNN-FAANEEEPNH-EQLVELA-RRISDEVRILELE-LGF---QD-KN---DDELET-VKSLSKRQAGN-RPG-SGIPMHVPVKMSGFPFGKRD-A
Go_pust     MARVRSLVLLLATL-CCYSSTSH-AD-DND-Y-GATEELNH-EQLVELG-RRITNEVRLLELE-LGI---QD-KND--QNQVDT-VKSLSKRQAVH-RAG-SGVPMNVPAKMSGFPFGKRN-A
As_love     MARMRNLVLLFVTL-CCSAPTSH-AN-DNG-Y-DATEERNR-EQLVELE-RIITD-VRLLEHE-LGL---QH-TN----DELDT-VKSLSKRQADN-RPG-SGVPMHVPVKMSGFAFGKRD-A
Op_john     MARVRNIVILFVTL-CCHASISY-AE-D-----ETTQELNK-EQLVEFA-RKIAGEVEFLKHT-LGM---QE-NN---GHQLDI-VKSLSKRQAV---------PMNVPVKMSGFTFGKRD-A
Op_lyma     MARVRNLVILFVTL-CCHASVSH-AE-DIE-F-EGTQDFSH-EQLVEFA-RRIAGEMELLELE-LGI---QD-KN---EDQMDI-VKSLSKRQAVD-R---HGVPMNVPVKMSGFAFGKRD-A
```

```
Am_cipu     QLVRR----------------------------SAQ--AKPVKLAGFAFGKR-GQLVKRSSDDQLMEEDET---EKRGALDAAFTFGKRR----DPSALSAFSFGKRRDPM-GLNALTFGKR
Am_cten     QLVRR----------------------------SAQ--AKPVKLAGFAFGKR-GQLVKRSSDDQLMEEDET---EKRGALDAAFTFGKRR----DPSALSAFSFGKRRDPM-GLNALTFGKR
Am_cons_1   QLVRR----------------------------SAQ--AKPVKLAGFAFGKR-GQLVKRSSDDQLLEEEET---EKRDALH-AFTFGKRR----DPSALSSFTFGKRRDPM-GLSALTFGKR
Am_cons_2   QLVRR----------------------------SAQ--AKPVKLAGFAFGKR-GQLVKRSSDDQLLEEDDT---EKRDALH-AFTFGKRR----DPSALSSFTFGKRRDPM-GLSALTFGKR
Op_filo     PLVRR----------------------------SAQ--AKPVKLTGFQFGKR-GQLEKRSADDKLMEEDET---EKRAALD-AFTFGKRR----DPSGLTAFSFGKRRDPL-GLNALTFGKR
Mi_grac     PLVRR----------------------------SAP--SKPVKLSGFIFGKR-AQLEKRSADDKLMEEDET---EKRAAFD-AFTFGKRR----DPSGLSAFSFGKRRDPT-RLSALTFGKR
Am_squa     PLVRR----------------------------SAQ--SKPVKLAGFAFGKR-GQLEKRSADDKLMEEDET---EKRALSS-AFTFGKRR----DPSGLSALTFGKRRDPM-GLSALTFGKR
Op_resi     QLVRR----------------------------SASSGAKPVKLAGFAFGKRAGQLVKRSSDDQLVEEDGA---EKRAAMD-AFTFGKRY----DPSGLSAFSFGKRRDPL-GLSALTFGKR
Op_savi     QLVRR----------------------------SASSGAKPVKLAGFAFGKRAGQLVKRSSDDQLLHEDGA---EKRAAMD-AFTFGKRY----DPSGLSAFSFGKRRDPL-GLSALTFGKR
Op_abys     SLVRR---------------------------SASSGGSKPVKLAGFAFGKR-GQLVKRSSDDQLLEEDST---EKRAAMD-AFTFGKRM---SDPSGLSAFSFGKRRDPM-GLSALTFGKR
Op_angu     QLVRR---------------------------SAKSGGDKPVKLAGFAFGKR-GQPVKRSTNDELEEDGE----EKRAAMD-AFTFGKRI---SDQE-LSPFSFEKRRDPT-GLSALTFGKR
Op_scha     QLVRR----------------------------SAGSGSKPVKLAGFAFGKR-GQLVKRSSDDQLEEEDEA---EKRAAMD-AFTFGKRL--SKDPSALSAFNFGKRRDPM-GLSALTFGKR
Op_reti     QLVRR----------------------------SAGSSSKPVKLAGFAFGKR-GQLVKRSSDDQLEAEDEA---EKRAAMD-AFTFGKRL--SNDPSALSAFNFGKRRDPM-GLSALTFGKR
Op_lame     QLVRR----------------------------SAGAGSKPVKLAGFAFGKR-GQLVKRSSDDQLEEEDEA---EKRASMD-AFTFGKRL--SNDPSALSAFSFGKRRDPM-GLSALTFGKR
Op_impr     QLVRR-----------------------------SASASKPVKLAGFAFGKR-GQPVKRSSDDQLEEEQDA---EKRAAMD-AFTFGKRP--SGDPTGLSAFNFGKRRDPM-SLSALTFGKR
Op_bisp     QLVRR----------------------------SAVAGSKPVKLAGFAFGKR-GQLVKRSSDDQLEEQDDA---EKRAAMD-AFTFGKRP--SGDPTGLSAFSFGKRRDPM-SLSALTFGKR
Op_vall     QLVKR---------------------------SAGNTGSKPVKLAGFAFGKR-GQLVKRSSDDQLEDDETAAATEKRASMD-AFTFGKRP--SSD---LSSFSFGKRRDP---LSALTFGKR
Op_brev     QLVRR----------------------------SAGAGSKPVKLAGFAFGKR-GQLVKRSSDDQLDEEQTT---EKRANLD-AFTFGKRK--AGD---LSAFSFGKRRDP---LSALTFGKR
Ba_hero     QLVRR----------------------------SAGAGNKPVKLAGFAFGKR-NQPVKRSSDDRTEEEE-----NKRGAMD-AFTFGKRP--SGNPTGLSAFSFGKRREPVGSLSALTFGKR
Op_appr     QLVRR----------------------------SAGAGSKPVKLAGFAFGKR-NQPVKRSSDDRADEEE-----DKRGAMG-AFTFGKRP--SGNPSGLSAFSFGKRREPLGSLSALTFGKR
Op_macu     QLVRR----------------------------SAGTGSKPVKLAGFAFGKR-N-PVKRSSDDRADEEE-----DKRGSMD-AFTFGKRP--SGNPSGLSAFSFGKRREPVGSLSALTFGKR
Op_vivi     QPVRR----------------------------SAGAGGKPVKLAGFAFGKR-NPLVKRSSDDKVEEQD-----DKRGAMD-AFTFGKRPSVSGDPSALSAFSFGKRRDPVGSLSALTFGKR
Op_cyli     QLVRR----------------------------SAGAGRKPVKLAGFAFGKR-NQLVKRSSDDQAEEEE-----DKRGAMD-AFTFGKRP--SGDPTGLSAFSFGKRRDPVGSLSALTFGKR
Op_wend     NLVRR----------------------------SAGAGSKPVKLAGFAFGKR-NQPVKRSSDDQIEEEE-----DKRGAMD-AFNFAKRP--SGDPSGLSAFSFGKRRDPVGSLSALTFGKR
Op_plic     QLVRR----------------------------SA----KPVKLAGFQFGKR-GQPVKRSSDDQAHEEE-----EKRGRMD-AFAFGKRL--SGDPSALSAFSFGKRRDPVSSLSALTFGKR
Op_fune     QLVRR----------------------------SA---SKPVKLAGFAFGKR-GQPVKRSPDDQAQEED-----EKRGPMD-AFAFGKRL--SGDPSALSSFSFGKRRDPVSGLSALTFGKR
Op_perf     QLVRR----------------------------SAG--SKPVKLAGFAFGKR-GQPVKRSSDDQLQEED-----EKRGALD-AFAFGKRR--SGDPSGLSAFSFGKRRDPASSLSALTFGKR
Cl_cana     QLVRR----------------------------SAGAGSKPVKLAGFAFGKR-GQPVKRSSDDQAQEEE-----DKRGSMD-AFTFGKRL--SGGKSALSAFSFGKRRDPVGSLSALTFGKR
Op_exim_1   QLVRR----------------------------SAGAGSKPVKLAGFAFGKR-NQPVKRSSDDQAQEEE-----DKRGSMD-AFTFGKRL--PGDPSALSAFSFGKRRDPVSSLSALTFGKR
Op_exim_2   QLVRR----------------------------SAGAGSKPVKLAGFAFGKR-NQPVKRSSDDQAQEEE-----DKRGSMD-AFTFGKRL--PGDPSALSAFSFGKRRDPVSSLSALTFGKR
Op_liod     QLVRRSAS--SGSKPKMSGFAFGKRDVQLVRR-SAGGSSKPVKLAGFAFGKR-SQPVKRSSDDQVEAQE-----DKRGALD-AFHFGKRL--SNDPSGLSAFSFGKR-EPMGSLSGLTFGKR
Op_prol     QLVRR----------------------------SAGAGSKPVKLAGFAFGKR-GQPVKRSSDDQA-EEE-----DKRGALD-AFTFGKRL--SSDP--LSAFNFGKRREPVSSLSALTFGKR
As_tubi     PLVRRSAG--AGAS-KMSGFAFGKRDSELVKR-SA---GKPVKLAGFAFGKR-SQLVKRSSDNVAENEE-----EKRGAMD-AFTFGKRL--SGDPSGLSTFSFGKRRNPGTSLSALTFGKR
As_bidw     PLVRRSAG--AGAS-KMSGFAFGKRDSELVKR-SA---GKPVKLAGFAFGKR-SQLVKRSSDNVAENEE-----EKRGAMD-AFTFGKRL--SGDPSGLSTFSFGKRRNPGTSLSALTFGKR
Op_oedi     PLVRRSAG--AGAS-KMSGFAFGKRDSELVKR-SA---GKPVKLAGFAFGKR-SQLVKRSSDNVAENEE-----EKRGAMD-AFTFGKRL--SGDPSGLSTFSFGKRRNPGTSLSALTFGKR
Go_pust     PLVRRSAKAAAGSA-KMSGFVFGKRDSELVKR-SASAGSKPVKLAGFAFGKR-SQLVKRSLDYEAENDE-----EKRGAMN-AFTFGKRL--SSDP------------------AAVTFEKR
As_love     QLVRRSAG--AGAA-KMSGFAFGKRDSEIVKR-SAGARSKPVKLAGFAFGKR-SQLVKRSSDNEEENDE-----EKRGARN-AFTFGKRL--SGNPSALSAFSFGKRREPGSALSALTFGKR
Op_john     QLVRR----------------------------SAG--SKPTKLAGFAFGKR-GQPVKRSSDNEAEDGQ-----EKRGTMD-AFAFGKRP--SGDPTGLSAFSFGKRRDPMSSLSALAFGKR
Op_lyma     PLVRR----------------------------SAGAGSKPVKLAGFAFGKR--NPVKRSSDNEANDKE-----EKRVPMD-AFAFGKRP--SGDPTGLSAFSFGKRRDPLSSLSALAFGKR
```

```
Am_cipu     -GMNPASGYSAFTFGKRGQMDNLHAFSFGKR-GMDPSGLSAFSFGKRGRDPSALSAFSFGKR-----------------MG-M-NAFTFGKREGL--E-EDGAFE-EENDD--EKRNQLSSLTGYTFGKR
Am_cten     -GMNPASRHSAFTFGKRGQMDNLHAFSFGKR-GMDPSGLSAFSFGKRDRDPSALSAFSFGKR-----------------MG-M-NAFTFGKREGL--E-EDGAFE-EENDD--EKRNQLSSLTGYTFGKR
Am_cons_1   -G-SP-SGYSAFTFGKRGQMDNLHAFSFGKR-GRDPSGLSAFSFGKRGRDPSALSAFSFGKR-----------------MGGM-KAFTFGKREDL--E-EEGAFA-EENDD--EKRNQLSSLTGYTFGKR
Am_cons_2   -G-SP-SGYSAFTFGKRGQMDNLHAFSFGKR-GRDPSGLSAFSFGKRGRDPSALSAFSFGKR-----------------MGGM-KAFTFGKREDL--E-EEGAFA-EENDD--EKRNQLSSLTGYTFGKR
Op_filo     --MSP-SGYSAFTFGKRGQMDNLHAFSFGKR-GMDPSSLSALTFGKRGRDPSSLSAFSFGKR-----------------MG-M-NAFTFGKRDEL--E-EDGAFE-DENDD--EKRSRLSSLTGYTFGKR
Mi_grac     -GMSP-SGYSAFTFGKRGRMDNLNAFSFGKR-GMDPSTLSAFSFGKRGRDPSALSAFSFGKR-----------------MG-M-NAFTFGKRDEL--E-EDGAFE-EENDD--EKR---------SYSKR
Am_squa     -GMNP-SGYSAFTFGKRGRMDNLNAFSFGKR-GMDPSGLSAFSFGKRGRDPSALSAFSFGKR-----------------MG---PAFTFGKRDE-----EDGAFE-EENYD--EKRSRIGALTGLTYGKR
Op_resi     -GMNP-SGMSAFSFGKR-RMEPLSAFSFGRKRGMDPSGLSAFSFGKRGMDPSGLSAFSFGKR-----------------MG-M-NAFTFGKREGG--EEEDPAFE-EENNN-EEKRAGYNGLSQFTFGKR
Op_savi     -GMNP-SGMSAFSFGKR-RMEPLSAFSFGRKRGMDPSGLSAFSFGKRGMDPSGLSAFSFGKR-----------------MG-M-NAFTFGKRESG--EEEDPAFE-EENNN-EEKRAGYNGLSQFTFGKR
Op_abys     -GMTP-SGMSAFSFGKR-RMEPLSAFSFGRKRGMDPSGLSAFSFGKRGMDPLGLNAFSFGKR-----------------MG-M-NAFTFGKREGL--EEEDAALE-EEDNNDDEKRAGYNGLSQFTFGKR
Op_angu     -GMHP-SSMSAFSFGKR-RMDPLSAFSFGKKRAMDPAGLSAFSFGKRGMDPSALSAFSFGKRGTGPS-GLSAFSFGKR-MG-M-NAFTFGKREGE--E-EETAFKKNTNDD--EKRAGYNGLSQFTFGKR
Op_scha     -GMDP-SGFSAFSFGKR-R-EPYSAFSFGKR-GMDPSALSAFSFGKRARDPSALSAFNFGKR-----------------MGGMTNAFTFGKREGL---EEDGAFE-EENQDEEEKRGGYNGIAGYTFGKR
Op_reti     -GMDP-SGFSAFSFGKR-R-EPYSAFSFGKR-GMDPSALSAFSFGKRARDPSALSAFNFGKR-----------------MGGMTNAFTFGKREDL---EEEGAFE-EENQDEEEKRGGYNGISGYTFGKR
Op_lame     -GMNP-SGFSAFTYGKR-R-EPLSAFSFGKR-GMDPSALSAFSFGKRGRDPSALSAFNFGKR----------------ANMGMTNAFTFGKRDDL---EEDGAFE-EEENQEEEKRGGYNGISGYTFGKR
Op_impr     -GMDP-SGFSAFNFGKR-R-DPFSAFNFGKR-GMDPTALSAFSFGKR-RDPSALSAFSFGKR-----------------MGGLTNAFTFGKRDDT---EEDGAFE-EENNN--EKR-DFSALAGYTFGKR
Op_bisp     -GMDP-SGFSAFSFGKR-R-DPFSALTFGKR-GMDPSALSAYSFGKRGRDPSALSAFNFGKR-----------------MGGLTNAFTFGKRDDA---EEDGAFE-EDNND--EKR-GFNGISGYTFGKR
Op_vall     -GMNP-AGFNAFSFGKR-R-DPFSAFSFGKR-GRDPSALGAFSFGKRGRDSNALGAFSFGKR-------------------GM-DAFTFGKREDL--D-VEGAF--DENED--EKRAYNNGMSGFSFGKR
Op_brev     -GMKP-SAFDAFSFGKR-R-DPLSAFSFGKR-GMDPNALGAFSFGKRGRD-NALGAFSFGKR-------------------GM-DAFTFGKRDD-----EEGAFE-DED----EKR-AYNPISAYTFGKR
Ba_hero     -GMDP-AGFSAFNFGKR-R-DPLSAFNFGKR-GMDPSGLSAFSFGKRGRDPSGLSAFSFGKRSRVP--SLSAFDFGKR--G-M-DAFTFGKREDL--D-EEGAFE-DENDD--EKR-GFNGISGYTFGKR
Op_appr     -GTDP-AGFSAFNFGKR-R-DPLSAFNFGKR-GMDATGLSAFSFGKRGRDPSGLSAFSFGKRGRVP--SLSAFDFGKR--G-M-DAFAFGKREDL--D-EDGAFE-DENED--EKR-GFNGISGYTFGKR
Op_macu     -GMDP-SGFSAFNFGKR-R-DPLSAFAFGKR-GMDASGLSAFSFGKRGRDASGLSAFNFGKRGRVP--QLSAFDFGKR--G-M-HAFTFGKREDL--D-EEGAFEDDENDD--EKR-GFNGISGYTFGKR
Op_vivi     -A-NP-SGFSAFNFGKR-R-DPLTAFNFGKR-AMDASGLSAFSFGKRGRDSNGLSAFSFGKRGRMP--SLGAFDFGKR--G-M-DAFTFGKREEL--D-DEGAFE-EENED--EKR-NFNGISGYTFGKR
Op_cyli     -GMDP-AGYSAFSFGKR-R-DPLSAFSFGKR-GMDATGLSAFSFGKRGRDPSGLSAFNFGKRGRMP--SLSAFDFGKR--G-M-DAFTFGKREDL--D-DEGAFE-DENDD--EKR-GFNGISGYTFGKR
Op_wend     -AMEP-AGFSAFSFGKR-R-DPLGAFSFGKR-GMDASGLSAFNFGKRGRDATGLSAFSFGKRGRVP--SLSAFDFGKR--GRM-DAFAFGKREDL--EEEDGAFE-DENDN--EKR-GYQGISGYTLGKR
Op_plic     -GMDP-SGFSAFNFGKR-R-DPLGAFSFGKRGGMDATGLSAFSFGKRGRDAAGLSAFSFGKRGRMP--SLSAFDFGKR--G-Y-DAFTFGKREGL--D-EEGAFE--ENDD--EKR--FNGISGLTFGKR
Op_fune     -GMDP-SGFSAFNFGKR-R-DPLGALTFGKRGGMDASGLSAFSFGKRGRDGAGLSAFSFGKRGRMP--SLSAFDFGKR--G-F-DAFTFGKREGL--DEEEGAFE--ENDD--EKR--FNGISGLTFGKR
Op_perf     -GMDP-SGFNAFNFGKR-R-DPLSAFNFGKRGGMDTSGLSAFSFGKRGRDASGLSAFSFGKRGRMP--SLSAFDFGKR--G-F-DAFTFGKREGL--DEGEGAFL-DENDD--EKR--FNGISGLTFGKR
Cl_cana     -GMDP-SGFSAFNFGKR-R-NPLSDFNLDKRGGMDASGLSAFSFGKRGRDATGLSAFSFGKRGRMP--SLSAFDFGKR--G-M-DAFTFGKREGL--D-EEGAFE-EENDD--EKR--FNGISGYTFGKR
Op_exim_1   -GMDP-SGFSAFNFGKR-R-DPLSAFNFGKRGGMDASGLSAFSFGKRGRDAAGLSAFSFGKRGRMP--SLSAFDFGKR--G-M-DAFTFGKREGL--D-EEGAFE-DENDD--EKR--FNGISGYTFGKR
Op_exim_2   -GMDP-SGFSAFNFGKR-R-DPLSAFNFGKRGGMDASGLSAFSFGKRGRDAAGLSAFSFGKRGRMP--SLSAFDFGKR--G-M-DAFTFGKREGL--D-EEGAFE-DENDD--EKR--FNGISGYTFGKR
Op_liod     -GMDP-SGLGAFSFGKR-R-DPLGAFNFGKRGGMDASGLSAFSFGKRGRNPTGISAFSFGKRGRVP--NLSAFDFGKR--G-M-DAFTFGKREDM--D-EEGAFE-DENEN--EKR-AYNGISGLTFGKR
Op_prol     -GMDP-SGFSAFSFGKR-R-DPLGAFNFGKRGGLDASGLSAFSFGKRGRDPSGMGAFSFGKRGRVP--NLSAFDFGKR--G-M-DAFTFGKREDM--D-EEGAFE-GENDD--EKR-AYSGISGYTFGKR
As_tubi     -GMYP-SGLSAFNFGKR-R-DPLSTFSFGKR-GVE-SGLSAFNFGKRGYDQSGLSAFSFGKRR-MPTGSLSAFNFGKR--G-M-NAFTFGKREDL--D-EEAAFE-DENND--EKR-AFNGMSGYTFGKR
As_bidw     -GMYP-SGLSAFNFGKR-R-DPLSTFSFGKR-GVE-SGLSAFNFGKRGYDQSGLSAFSFGKRR-MPTGSLSAFNFGKR--G-M-NAFTFGKREDL--D-EEAAFE-DENND--EKR-AFNGMSGYTFGKR
Op_oedi     -GMYP-SGLSAFNFGKR-R-DPLSTFSFGKR-GME-SGLSAFNFGKRGYDQSGLSAFSFGKRR-MPTGSLSAFNFGKR--G-M-NAFTFGKREDL--D-EEAAFE-DENND--EKR-AFNGMSGYTFGKR
Go_pust     -GMNP-SGISAFNFGKR-R-DPFSTFSFGKR-GMESTGLSAFNFGKRGYDQSGLSAFSFGKRR-WPTNSLSAFDFGKR--G-M-NAFTFGKRKYL--D-EEGAFG-DENKD--EKR-AYNAMYGYTFGKR
As_love     -GMNP-SALSAFNFGKR-R-DPLSAFSFGKR-GMQ-SGLSAFNFGKRGYDENGLSSFSFGKRR-MPTGSLSGFDFGKR--G-M-DAFTFGKREDL--N-EEGAFD-DENND--EKR-AFNGISGYTFGKR
Op_john     -GMDR-SGFNAFSFGKR-R-DPLSAFSFGKR-GMD--RLNAFNFGKRGRNLGSLSAFDFGKR------------------G-M-DAFAFGKRENL--D-EDGAFE-DED----EKR-AFDGLSAYAFGKR
Op_lyma     -GMDP-SGFNAFSFGKR-R-DPLSAFSFGKR-GMD--GLNAFNFGKRGRDSASLSAFNFGKRGRMPMGSLSAFDFGKR--G-M-DAFAFGKREDL--D-EEGAFQ-DENDD--KKR-AFNGLSGYAFGKR
```

```
Am_cipu    DTQ-----------AAAD--DNTLN-QEETLRTN---
Am_cten    DTQ-----------AAAE--DNTLN-QEETLRTN---
Am_cons_1  DTQE---------AAAAD--DNTLN-QEETLRTN---
Am_cons_2  DTQE---------AAAAD--DNTLN-QEETLRTN---
Op_filo    DTH----------EAAAE--DNALN-QEETMRTD---
Mi_grac    DTQ----------EAAAE--DNTLY-QEETLRTD---
Am_squa    DTQ----------EAAAD--DNTLN-QEETLRTE---
Op_resi    DTQ----------AAAAE---ETLNHNDETLRTD---
Op_savi    DTQ----------AAAAE---ETLNHNDETLRTD---
Op_abys    DTQ-----------AAAE---ETLNHNDEILRTD---
Op_angu    DTE------------AVE---ERLN-NDETLRDD---
Op_scha    DTK-----------AAAD---ESLNHNEETLRTD---
Op_reti    DTK-----------AAAD---ESLNHNEETLRTD---
Op_lame    DTK-----------AAAE---DTLNHNDETLRTD---
Op_impr    DTK-----------AAAD---DTLNQNEETLRTD---
Op_bisp    DTK-----------AAAE---DRLNHNDETIRTE---
Op_vall    DTK-----------AAAE---DTLNENDETLRTD---
Op_brev    DTE-----------AAGD---DTLNHNEETLRTD---
Ba_hero    DTN-----------AAAEDDADTLNHNDETLRTD---
Op_appr    DTD-----------AAAEDDADTLNHNDETLRTN---
Op_macu    DTN-----------AAAEDDADTLNHNDETLRTD---
Op_vivi    DTH-----------AAAADNADTLNHNDETLRTD---
Op_cyli    DTN-----------AAAE-DADTLNHKDETLRTD---
Op_wend    DTN-----------AAAE-DAERLDHNDETLRTD---
Op_plic    DTD-----------AAAN-DADTLN-NDDILRTD---
Op_fune    DTD-----------AAAN-DADTLN-TDDTLRTN---
Op_perf    DTN-----------AAAD-EADILNHNDEILRTD---
Cl_cana    DTN-----------AAAE-DADTLNHNDETLRTD---
Op_exim_1  DTN-----------AAAE-DADTLNHHDETLRTD---
Op_exim_2  DTN-----------AAAE-DADTLNHHDETLRTD---
Op_liod    DTN-----------AAVE-NADTLNNNDETLRTD---
Op_prol    DTN-----------AAAE-DADTLNHNDETLRTD---
As_tubi    DTD-----------AADD-TEDTLNHNDEILRTDN--
As_bidw    DTD-----------AADD-TEDTLNHNDEILRTDN--
Op_oedi    DTD-----------AADD-TEDTLHHNDEILRTDN--
Go_pust    DTD-----------AAED-AEETLNHNDETQRTDN--
As_love    DTE-----------EA----EDTLNNNADTLRTDN--
Op_john    DT------------VAAD---DTLNHNEETLRTDN--
Op_lyma    DTD-----------AAAE---DTLNHNDETLRTDN--
```

L-type SALMFa

```
Am_cons_1   MRLQPRFVWFFICALVPIVLAGTIPRRNSGGKEVPTSFHYDVLVKDQ-QMENEDRDIEERRSGRGRTY-LNSGLLFGKRFDDTG-DFLLT-DD-----DL
Op_pilo     MRLQPRFV-FFICALVPIVLAGTIPRR--GGNEVPA-FNYDAMVKDK-QMENEDRDIEERRSGR-RTY-LNSGLLFGKRFDET--GDFLT-DE-----EE
Op_resi     MRLIPGLV-LFICALVPIIAAGTIPRR--TSKELPT-YNYDAMVKDQ-QMENEDREIEERRSGRSRTY-LNSGLLFGKRFAETA-DDFIN-ED-----NT
Op_savi     MRLIPGLV-LFICALVPIIAAGTIPRR--TSKELPT-YNYDAKVKDQ-QMENEDREIEERRSARSRTY-LNSGLLFGKRFGETA-DDFIN-ED-----NT
Op_abys     MRLQPRLV-LFICALVPIIAAGTIPRR--TSKELPT-YNYDAMVKDQ-QMENEDREIEERRSGR-RTY-LNSGLLFGKRFGETA-DAFIN-DD-----DT
Op_angu     MRLQARLV-LFICAIIPVIAAGVVPRR--ASKESPT-YNYDAMVKDP-QMANQDKEIEERRSGRSRTY-LNSGLLFGKRFEEAA-DDFIN-DE-----NT
Op_caes     MRLQARLV-LFICALIPVIAAGVAPRR--STKELPT-YNYDAMVKDP-QMANQDREIEERRSGRSRTY-LNSGLLFGKRFEEAA-DDFIN-DD-----DP
Op_impr     MRLQPRLV-FFICALVPIIAAGTIPRR--TGIEVPT-FNYDAKVKDQ-QMENEDREIEERRSGR-RTHSLNSGLLFGKRFEET--EDFLN-DD-----DT
Ba_hero     MRLQPLLV-FFICALVPFVVAGTIPRR---NFEVPN-YNYDAMVKDQ-QMENEDKEVEERRSGR-RTHSLNSGLLFGKRFEET--DDFIN-DD-----ET
Op_plic     MRLQPVLV-FFICAIVPFVAAGTIPRR--TGFEVPN-YNYDAMVKNQ-QMENEDKEVEERRSGR-RTHTLNSGLLFGKRFEET--DDFIN-DD-----EL
Op_fune     MRLQPVTV-FFICAIVPFVAAGTIPRR--TGFEVPN-YNYDAMVKNQ-QMENEDKEVEERRSGR-RTHTLNSGLLFGKRFEET--DDFIN-DD-----ES
Op_perf     MRLQPMLV-FFICAIVPFVAAGTIPRR--TGFEVPN-YNYDAMVQDK-PMENQNKEVEERRSGR-RTHTLNSGLLFGKRFEET--DDFIN-DD-----ES
Cl_cana     MRLQPLLV-FFICAIVPFVAAGTIPRR--TGFEVPN-YNYDAMVKDQ-QMENEDKEVEERRSGR-RTHTLNSGLLFGKRFEET--DDFIN-DD-----ET
Op_liod     MRLQPLLV-FVICALVPFVAAGTIPRR--TGFEVPN-YNYDAMVKDQ-QMENEDKEIDERRSGR-RTHTINSGLLFGKRFEET--DDFIN-DD-----ET
As_tubi     MRLQPLFV-CFICALVPFVATGRIPRR----LEAPQ-YNYAAMVKS--HLEDDNKEIEERRSGRRNNPSLNSGLFFGKRFDGT--DEFVN-DD-----ET
As_bidw     MRLQPLFV-CFICALVPFVATGRIPRR----LEAPQ-YNYAAMVKS--HLEDDNKEIEERRSGRRNNPSLNSGLFFGKRFDGT--DEFVN-DD-----ET
Op_oedi     MRLQPLFV-CFICALVPFVATGRIPRR----LEAPQ-YNYAAMVKS--HMEDDNKEIEERRSGRRNNPSLNSGLFFGKRFDGT--DEFVN-DD-----ET
As_love     MRLQPLFV-CFICAIVPFVATGRLPRR----FEAPQ-YNYAAMVKDR-TMEDDNKEIEERRSGRRNPPGLNSGLFFGKRFDGT--DDFVN-DD-----ET
Gl_sp_no    MRLQPLLV-FCICALVPFAATGTIPRR-RSGFEGAN-YNYDVLVKDTAQLEDEDKEIEERRSGR-RNPSLNSGLIFGRRFEETAADDFLN-DD-----ES
Op_john     MRLQPLLV-CFICALVPFVATGTIPRR--TAFEGHN-YNYDAMVKDT-QMENENKEIDERRSGRRRGPALNSALIFGKRLEGT--DDFLN-DD-----ET
```

```
Am_cons_1   DEDTRHFNVETRGRNRLPFHSALMQGKRTPPLDEEDMLTSRSKKRSRP--LFHSGIMMGKRYPSLFSEDAAVD-------KRKS---RLRWSDGMLFGK-
Op_pilo     D--NRHFNVEIRGRNRLPFHSALMQGKRT-PLEERDL-MSRSKKRTRP--VFHSGIMMGKRYQL-YSDDAVD--------KRKGS--RLRWSDGMLFGK-
Op_resi     R-QQQQFTVDIRGRNRLPFHSGLMQGKRS-PEVEREP-ESGNRKRTRP--AFHSAMLLGKRFPN-FEFDAADD--DLEEFKRKAS--RLRWSTGTTFGRK
Op_savi     RQQQQQFTVDIRGRNRLPFHSGLMQGKRN-PQDEREP-ESSI-KRGRP--KFHSAMAFGKRYPN-FEFDAADD--DLEEFKRKAS--RLRWATGTQFGRK
Op_abys     R-QQQQFTVDIRGRNKLPFHSGLMQGKRS-PQQENGP-DSNDWKRSRP--KFHSAMLLGKRLPN-FEFDAADDAQDIDEFKRKAS--RLRWSNGMAFGRK
Op_angu     N-QQQQFTVEVRGRSKLPFHSALMQGKRS-SLEDEF--DSSFSKRGKQKAKFHSAMLLGKRYPE-FGIDAVDGVQNIDEFKRKGS--RLRWSNGMAFGRK
Op_caes     N-QPQQFTVEVRGRSKLPFHSALMQGKRN-SLEDES--DSSFNKRGKPKAKFHSAMLLGKRYPE-FGINEVDDHQDIEEFKRKGS--RLRWSNGISFGRK
Op_impr     R----EINVEIRGRNRLPFHSALMQGKRT-PQQEE---ENSAKKRSRP--VFHSGFMMGKRFPM--EYDAADDA-NIEEFKRKAA--RLRWSNGMQFGK-
Ba_hero     R----QINVDIRGRNKLPFHSGLMQGKRN-PEQEE----ADNAKRTRP--IFHSGFLMGKRYP----SESDDD---LEEFKRKAG-ARLRWSDGMQFGK-
Op_plic     R----QFNVDIRGRNKLPFHSGLMQGKRD-PQQ------GISDKRSRP--AFHSGFLMGKRYPP---FEADND---LEEFKRR---QRLMWSNGISFGK-
Op_fune     R----QFNVDIRGRNKLPFHSGLMQGKRD-SQQ------EISNKRSRP--VFHSGFMMGKRYPP---YEIDND---LEDFKRKAG-QRLMWSNGISFGK-
Op_perf     R----QINLDIRGRNKLPFHSGLMQGKRD-PQQEQ---REISNKRSRP--VFHSGFLMGKRYP----YENDND---LEEFKRKAG-QRLMWSNGISFGK-
Cl_cana     R----QINVDIRGRNKLPFHSGLMQGKRN-PQQDQ---QEISDKRSRP--VFHSGFLMGKRYP----YEIDDD---LEEFKRKAG-QRLMWSNGIQFGK-
Op_liod     K----QINVDIRGRHKLPFHSALMQGKRN--QQSG---EEISAKRTRP--VFHSGFMMGKRYQ----MESADDA-NLEDVKRKAGYARLRWSDGMQFGK-
As_tubi     R----QINMEIRGRSKLPFHSGLMQGKRN-QQEEEGM----TEKRTRP--VFHSGIMMGKRYPS------IGDANDLEEFKRKAG-QRLRWSNGMQFGK-
As_bidw     R----QINMEIRGRSKLPFHSGLMQGKRN-QQEEEGM----TEKRTRP--VFHSGIMMGKRYPS------IGDANDLEEFKRKAG-QRLRWSNGMQFGK-
Op_oedi     R----QINMEIRGRSKLPFHSGLMQGKRN-QQDEEGM----TEKRTRP--VFHSGIMMGKRYPS------IGDANDLEEFKRKAG-QRLRWSNGMQFGK-
As_love     R----QINVDVRGRNKLPFHSGLMQGKRN-QQEEQGM----TEKRSRP--VFHSGIMMGKRFPD----------DDLEEFKRKAG-QRLRWSDGMQFGK-
Gl_sp_no    R----QINLEIRGRSRLPFHSGLMQGKRN-PQQDDL-----SVKRSRP--AFHTGFLMGKRFPE------ADDL-DLEEFKRRTG-QRLRFSDGMLFGK-
Op_john     K----QIKVDIRGRSRLPFHTGFMQGKRN-PQQVDQ-----SAKRTRP--VFHSGFMMGKRFPT------ADEM-DLEEFKRKAG--RLRFSDGMLFGK-
```

Tachykinin

```
Am_cipu       MASI-DWTKNLPAVALIVCGVLFCNGQA-QLAAENTGALKEITDFDDETQ-YPELDL-DWIQPENAAFMK--LSPE---L-WENVPLQ-YVKRRKNQVFSAGLFGKR--SGWNQGHQNGLFGKRS-DWLEEFMSG---
Am_cons_1     MAFR-NWTQNLPAVALIVCGVLFCHGQG-QLTADNTGALKEITEFEDETQ-YPEVDL-DWIEPED-DFLNP--------ISF-NVPLQ-YVKRRKNQVFSAGLFGKR--SGWNQGHQNGLFGKRS-DWLEEFMSG---
Am_cons_2     MAFI-NWTQNLPAVALIVCGVLFCHGQG-QLTADNTGALKEITEFEDETQ-YPEVDL-DWIEPED-DFLNP--------ISF-NVPLQ-YVKRRKNQVFSAGLFGKR--SGWNQGHQNGLFGKRS-DWLEEFMSG---
Op_filo       MASI-DWTKRLPAITLIICGVLFCHGQA-QLPADSNGALKEITEFDNDT---PELDL-DWIEPEDAAF-----------LSWDNVPLQ-YVKRRKNQVFSAGLFGKR--SGWNQGHQNGLFGKRS-DWLEDYMSG---
Mi_grac       MASM-DWTKKLPAVALIICGVLFCHGQA-QLSAENTGALKEITEFEDENQ-YPELDL--VFEPEDAALLQPLLSPD---LSWENVPLQ-FVKRRKNHVFSAGLFGKR--SGWNQGHQNGLFGKRS-DWFEEYMSG---
Am_squa       MASI-DWTKKLPAAVLIIFGVLFCHGQA-EFSAENAGALKEITDFNDETQ-FPEVDQRDWIEPEEADFLKPLLSQDFQVLNWENVPLQ-YVKRRKNQVFSAGLFGKR--SGWNQGHQNGLFGKRS-DWLEEYMSG---
Op_resi       MVAI--WTRKLPAVALFICGVLFCNGQG-QLSHENNGALNEITEFNDDTQ-YPELDP-EWIRQDDTAALKPLLSPE---LSWENVPLQ-YVKRGKSQVFSAGLFGKR-SSGWNQGHQNGLFGKRTSDWLDAYLSG---
Op_abys       MVAIKDWTRKLPAVALFICGVLFCNVQG-QLSPENTGALNEITQFNDDTQ-YPDLDP-EWITQEDTAALNPLLSPE---LSWENVPLQ-YVKRRKNQVFSAGLFGKR-SSGWNQGHQNGLFGKRTTDWLEEYLSG---
Op_impr       MASIKNWTKKLPAVALIICGVLFCSGQG-QLSPENTGALNEITEFDEETQ-YPELEP-DWIQPEDAAFLNPFLSPN---LSWENVPLQ-YVKRRKNQVYSAGLFGKR--SGWNQGHQNGLFGKR--NWLEEYMSG---
Op_vall       MASMKNWINKLIAVTLILCGVLFSNGQGLQLSSENAGALTEITEFDDETQ-YPDADP-DWIEPEDAAFLNPLLSPD---LSWENVPLQ-YVKRRKNQVFSAGLFGKR--SGWNQGHQNGLFGKRSNTWLEEYMNG---
Ba_hero       MASIKSWTKKLLAVALIICGVLFCNGQG-QLTPENTGALNEIIEFDDETQ-YPDLDP-DWIEPEDAAFLGPLLSPD---ISWENVPLQ-YVKRRKNQVFSAGLFGKR--SGWNQGHQNGLFGKRSNNWLEEYVSG---
Op_cyli       MASIKSWTKKLPAVALIICGVLFCNGQG-QLSPENTGALNEITEFDDETQ-YPDLDP-DWIEPEDAAFLGPLLSPD---LPWEKVPLQ-YVKRRKNQVFSAGLFGKR--SGWNQGHQNGLFGKRSNNWLEEYVSG---
Op_wend       MASIKSWTKRLPAVALIICGVLFCHGQG-QLTPEDPGALNEITEFDDDTQ-YPDLDP-DWIEPEDAAFLGPLLSPD---LSWDNVPLQ-YVKRRKNQVFSAGLFGKR--SGWNQGHQNGLFGKRSNNWLEEYVSG---
Op_plic       MASMKCWTKKLPAIALIICGVLVCNGQC-QLTADDTGALNEITEFDDETQ-YPNLDP-DWIAPEDAAFLGHLLSPD---LSWENVPLQ-YVKRRKHQVFSAGLFGKR--SGWNQGHQNGLFGKRSNNWLEEYVTG---
Cl_cana       MASMKSWTKKLPAIALIICGVLVCNGQC-QLTPDNTGALNEITEFDDETQ-YPNLDP-DWIEPEDAAFLGPLLSPD---LSWENVPLQ-YVKRRKNQVFSAGLFGKR--SGWNQGHQNGLFGKRSNNWLEEYVTG---
Op_prol       MASIKSWTKKLPAVALIICGVLFCNGQG-QLTPEKTGALNEITEFDDDTQ-YPDLDP-DWIEPDDAAFLGPLLSPD---LSWENVPLQ-YVKRRKNQVFSAGLFGKR--SGWNQGHQNGLFGKRSNNWLEEYVSG---
As_tubi       MASLKNWTRNLPALTLIICGSLFCNGQG-QLAPDSTGALNDLTEFDDETQ-FPKIDP-DWINQEDAVFLGPLLSND---LTLDNVPLQ-YVKRRKNQVFSAGLFGKR--SGWNQGHQNGLFGKRSNNWLKEYVSG---
Gl_sp_no      --SVKSWTRNMQAVALIFCGVFFCSGQG-QLTPDKSGALNELTEFNDDTT-YPDMDP-DWIDPEDAAFLAPVLTPE---LTWEHVPLQ-LVKRRKNNVFSAGLFGKR--NGWNQGQQTGLFGKR--NWFEEYVSSARA
Op_john       MASVKSWTKKLPAVALIICGVLFCNGQG-QLTSDNTGALNEITGFNDDTQ-FPDLDP-DWIEQEDAAFLGPILSPD---SSWETVPLQ-FIKRRKNNVFSAGLFGKR--SGWNEGHQNGLFGKRSTNWLEEYVTG---

Am_cipu       -------GE-DGDDNED------SSSQYA-KK-QWNPNQQTGGLFGKRN------DQFAKRTYGDMLQSLHDRVESAM-DKRKAA---ATLG---RVRTKSSGQHVFRTGGLFGKRSVEDPGMQRALWPEDEQRRK---
Am_cons_1     -------GD-ESDDNSE-------SSQYA-KK-QWN-DQQKSGLFGKRS------DQFARRTYSDMLQSLHDRVETAM-DKRKAAA--ATLG---RVRTKSSGQHVFRTGGLFGKRSAEEPGMQRALWPEEEQRRK---
Am_cons_2     -------GD-ESDDNSE-------SSQYA-KK-QWN-DQQKSGLFGKRS------DQFARRTYSDMLQSLHDRVETAM-DKRKAAA--ATLG---RVRTKSSGQHVFRTGGLFGKRSAEEPGMQRALWPEEEQRRK---
Op_filo       -------TD-DSADNTE-------SNQYA-KK-QQNPNQQTGGLFGKRN------DQVMRRTYDVMLQSLHDRVASAM-EKRKASA--ASLG---RVRTKSSGQHVFRTGGLFGKRSAEEPGMQRALWPEDEQRRK---
Mi_grac       -------SE-DSGDNAE-------SSQYA-KK-QWNPNQQTGGLFGKRN------DQLTRRTYDVMLQNLHDRVESAM-DKRKAS---PAIG---RVRTKSSGQHVFRTGGLFGKRSAEEPGMQRALWPEDEQRRK---
Am_squa       -------SD-DSGDNAAE----SSSSQYA-KK-QWNPNQQTGGLFGKRS------DQLARRTYDLMLQNLHDRVESAI-DKRRAS---DSVG---RVRTVKSGQHVFRSGGLFGKRSAEEADMQRALWPEDEQRRK---
Op_resi       -------DD--------------EADHYSEKR-EWNNNQKT-GMFGKRH------SDVEQRTYEDILQSLHDKLM----DKRTA-----NVG---RVRTKSSGQHVFRSAGLFGKRSAEEPEMEKALWPEDEQRRK---
Op_abys       -------NN-DEADNEY-------------KR-EWNPNQKT-GMFGKRN------SEVKRRTYGDILQSLQDKLV----EKRGAA----SLG---RVRTKLSGQHVFRTGGLFGKRSAEEPDMEQALWPEEEQRRK---
Op_impr       -------RE-QEDAADA-------GNTQYAKRQ-WSPNQQTGGLWGKRN------SEAARRTYEEMLQSLHDKVESEM-DKRSSP----NLG---RVRTKSSGQHVFRTGGLFGKRSVEEPEMQRALWPEDEQRRK---
Op_vall       -------RENDGDEAAD-------AVLQYAKRQRWNPNQQTGGLFGKRN------SHVV-RTYEDMLQSLHDKVESAMVEKRANAA--ANSG---RVRTKSSGQHVFRTGGLFGKRSTEDAGMQRALWPEDEQRRE---
Ba_hero       -------RD-DADDKAD--------TTKYSKRQRWNPNQQTGGLFGKRN------SEVVKRTYEEMLQTLQDKVESAM-DKRSPA----NLG---KIRTKSSGQHVFRTGGLFGKRSAEEPDMQRALWPENEQRRK---
Op_cyli       -------RD-DADDEAD--------TTKYAKRQSWNPKQHTTGLFGKRN------SEVKRRTYEEMLKTLQDKVQSAM-DKRSAA----NLG---RVRTKSSGQHVFRTGGLFGKRSAEEPEMQRALWPENQQRRK---
Op_wend       -------RD-GADGEAD--------TTKYAKRQ-WNPNQHTGGLFGKRN------SEVIRRTYEEMLQSLQDKVQSAV-DKRSAA----NLG---RVRTKSSGQHVFRTGGLFGKRSAEEPEMQRALWPEEEQRRK---
Op_plic       -------RD-DED-KED--------ATNYSKRQRWNQNQQTGGLFGKRN------SDDLRRTYEEMLQTLQDKVESAV-DKRSAA----NLG---RVRTKSSGQHVFRTGGLFGKRSVEDPQMQRALWPEDEQ------
Cl_cana       -------RD-DED-EAN--------TTHYSKRQRWNPNQQTGGLFGKRN------SDVLRRTYEEMLQTLQDKVESAV-DKRSPA----NLG---RVRTKSSGQHVFRTGGLFGKRSAEEPAMQRALWPEEEQRRK---
Op_prol       -------RD-DEKDKSD--------TTQYAKRQRWNQNQQSTGLFGKRN------SEVVRRTYEDMLQSLQDKVETAM-DKRSAA----NLG---RVRTKSSGQHVFRTGGLFGKRSVEDPGMQRALWPEDAQRRK---
As_tubi       -------SDEAAADSPLE---YGQAAGQYAKRQRWNPNQQN-GLFGKRN------SDLVKRTYDNMLQTLREKVKSAI-DKRSAA---TNLG---RVRTKSSGQHVFRAGGLFGKRSAEEPELQRALWPENEQRRQTNA
Gl_sp_no      VDAAAANAD-EAVDTTDE---YGLNAGQYAKRQRWNQNQQP-GLFGKRNHAADDTAAAARRTYKEMLKTLQDKVESTI-DKRT-----NNPG---RVRTKSSGQHVFRSGGLFGKRSAEAPGMQRALWPENEQRRK---
Op_john       -------RD-DVEEAGGTAQDFGQTAGQYAKRQRWNQNQQTGGLFGKRN-----TDDAARRTYEEMLKTLQEKVASTM-DKRSPT---NNIG---RVRTKSSGQHIFRTGGLFGKRSAEAPGMERALWPENEQRR----
```

TRH-1

```
Am_cipu     MQDLMNIRGGLV-PLFILVALSTVCCLAD----------------TE-GWPNE--EVETVDLLDDEEKRQFAPGKRLLLLGKREWVGGEE---GY
Mi_grac     MQNLVTLRGGLG-PLFLFVVLSSVCCLAEPGELE---AANDITPLREDGWPDD--EGEAL--YEDEAKRQFAPGKRLLLLGKRQWVGGMP---EM
Op_angu     --------------ICLLVLAGCMSCFGDPVDLE--NTANDITPLKE-GWPDN--EVQTFEELEDEVKRQFAPGKRLLLLGKREWVGGMP---EM
Op_lame     MQDPTIVRGCLV-PLFLLLALGTVCCLADPADLE---GTNDITPLRE-GWPDN--EDEALEELENEVKRQFSPGKRLLLLGKRQWVGGMP---EM
Op_impr     MQELIIVRGCLV-PLFFLLALGTVCSLADPADLE--GAANDITPLRE-GWPDTGLEDDELEDLDNEVKRQFSPGKRLLLLGKRQWVGGFPLEFEN
Ba_hero_a   MQEPRYNRGNLV-PLFLLLALGTVCCLAVPGDF----TANDITPLKEGGWLNNGLEDATLEELQNEVKRQFSPGKRLLLLGKRQWIGGMP---EL
Ba_hero_b   MQEPRYNRGNLV-PLFLLLALGTVCCLAVPGDF----TANDITPLKEGGWLNNGLEDATLEELQNEVKRQFSPGKRLLLLGKRQWIGGMP---EL
Op_vivi     MQEPRYNRGNLV-PLFLLLALGTVCCLADHVDLD---DANDIAPLKE-GWPDNRLEDATLEELQNEVKRQFSPGKRLLLLGKRQWVGGMP---EM
Op_perf     MQELRYNRGNLV-PLFLLLALGTVCCLADPVNDI---SANDITPLRE-GWPDNELAEEPYEG----VKRQFSPGKRLLFLGKRQWVGGMP---EM
Cl_cana     MQELRYNRGYLV-PLFLLLALGTVCCLADPADLE---TTNDITPLRE-GWPDNGLEDETFEEMQ--VKRQFSPGKRLLFLGKRQWVGGMP---EM
Op_exim_1   MQELRYNRGYLV-PLFLLLALGTVCCLEDPADLE--GTANDITPLRE-GWPDNGLEDETFEELQNEVKRQFSPGKRLLFLGKRQWVGGMP---EM
Op_exim_2   MQELRYNRGYLV-PLFLLLALGTVCCLEDPADLE--GTANDITPLRE-GWPDNGLEDETFEELQNEVKRQFSPGKRLLFLGKRQWVGGMP---EM
Op_liod_a   MQEARYYRGCLG-PLFLLLALGTVCCLADPADLE--DAANDVTPLRE-DWPGNDLEDVTLEELQYEVKRQFSPGKRLILLGKRQWVGGMP---EM
Op_liod_b   MQEARYYRGCLG-PLFLLLALGTVCCLADPADLE--DAANDVTPLRE-DWPGNDLEDVTLEELQYEVKRQFSPGKRLILLGKRQWVGGMP---EM
As_bidw     MQEPRYNRSCLLSPLLLLLALGTV-CFADVGELD--DTANDVTPPKE-GWTNNALEDESLDALENEVKRQFSPGKRLLLLGKRQWVGGMP---ET
Op_oedi     MQEPRYNRSCLLSPLLLLLALGTV-CFADVGELD--GTANDVTPLKE-GWTNNALEDESLDALGNEVKRQFSPGKRLLLLGKRQWVGGMP---ET
As_love     MQEPRYHRTCLLSPILLLLALGTL-CRADGGELD--RTANDIAPLSN-GWTNNALEDDALEVLDNEVKRQFSPGKRLLLLGKRQWVGGMP---EM
Op_john     MQESRYHRGCLL-PLLFLLALGTV-CLADLGDLELEGAANDIRPLRE-GWPENGLE----EELENEVKRQFSPGKRLLLLGKRQWIGGMP---EM
Op_lyma     MQEPRYRRGCLV-PLLLLIALGTV-CLADLGDLELEGTANDITPFRE-GWPDNGLEDETLDELENEVKRQFSPGKRLLLLGKRQWIGGMP---EM

Am_cipu     SDDPFSPDKRQFSAGKRQFSAGKRQFSAGKRQFSAGKRQFSAGKRQWLGGEEE---YDPEE----------NLNMETRQFSAGKRQFSAGKR---
Mi_grac     VDMPEE---RQFSAGKRQFSAGKRQFSAGKRQFSAGKR-----------------------------------------------------------
Op_angu     VDMPET---RQFSAGKRQFSAGKRQFSAGKRQFSAGKRQFSAGKRQWVGGEEDDGLEENDDMKRQFSAGKRQFSAGKRQFSAGKRQFSAGKR---
Op_lame     VDMPET---RQFSAGKRQFSAGKRQFSAGKRQFSAGKR-------QWVGGEPEE--WEDEDMKRQFSAGKRQFSAGKRQFSAGKRQFSAGKR---
Op_impr     DDM------------KRQFSAGKRQFSAGKRQFSAGKRQFSAGKRQWVGGFPLE--FEDEDVKRQFSAGKRQFSAGKRQFSAGKRQFSAGKR---
Ba_hero_a   VDMPET---RQFSAGKRQFSAGKRQFSAGKRQFSAGKR-------QWVGGEPD---VLNQDEKRQFSAGKRQFSAGKRQFSAGKRQFSAGKRQFS
Ba_hero_b   VDMPET---RQFSAGKRQFSAGKRQFSAGKRQFSAGKR-------QWVGGEPD---VLNQDEKRQFSAGKRQFSAGKRQFSAGKRQFSAGKR---
Op_vivi     VDMPET---RQFSAGKRQFSAGKRQFSAGKRQFAAGKR-------QWVGGEPDE--FD-EAQKRQFSAGKRQFAAGKRQYAAGKRQFTAGKR---
Op_perf     VDMPET---RQFSAGKRQFSAGKRQFSAGKRQFSAGKRQFSAGKRQWVGGEP-----DEEEEKRQFSAGKRQFSAGKRQFSAGKRQFSAGKR---
Cl_cana     VDMPET---RQFSAGKRQFSAGKRQFSAGKRQFSAGKR-------QWVGGEPDE--FDDEEEKRQFSAGKRQFSAGKRQFSAGKRQFSAGKR---
Op_exim_1   VDMPET---RQFSAGKRQFSAGKRQFSAGKRQFSAGKR-------QWVGGQPDL--LDDEEEKRQFSAGKRQFSAGKRQFSAGKRQFSAGKR---
Op_exim_2   VDMPET---RQFSAGKRQFSAGKRQFSAGKRQFSAGKR-------QWVGGQPDL--LDDEEEKRQFSAGKRQFSAGKRQFSAGKRQFSAGKR---
Op_liod_a   VDMPET---RQFSPGKRQFSPGKRQFSPGKRQFSPGKRQFSPGKRQWVGGESDE--FEDEEEKRQFSPGKRQFSPGKRQFSPGKRQFSPGKR---
Op_liod_b   VDMPET---RQFSPGKRQFSPGKRQFSPGKRQFSPGKR-------QWVGGESDE--FEDEEEKRQFSPGKRQFSPGKRQFSPGKRQFSPGKR---
As_bidw     VDMPET---RQFSAGKRQFSAGKRQFSAGKRQFSAGKR-------EWMDDGPDM--LEEEDEKRQFSAGKRQFSAGKRQFSAGKRQFSAGKR---
Op_oedi     VDMPET---RQFSAGKRQFSAGKRQFSAGKRQFSAGKR-------EWMDDGPNM--LEEEDEKRQFSAGKRQFSAGKRQFSAGKRQFSAGKR---
As_love     VDMPET---RQFSAGKRQFSAGKRQFSAGKRQFSAGKR-------EWM-DEPDM--LDEEDAKRQFSAGKRQFSAGKRQFSAGKRQFSAGKR---
Op_john     VDMPQT---RQFSAGKRQFSAGKRQFSAGKRQFSAGKR-------QWIGGAED----ENEEAKRQFSAGKRQFSAGKRQFSAGKRQFSAGKR---
Op_lyma     VDIPQT---RQFSAGKRQFSAGKRQFSAGKRQFSAGKR-------QWIGGEDD----ANEEAKRQFSAGKRQFSAGKRQFSAGKRQFSAGKR---
```

```
Am_cipu      ----QFSAGKRDWEEE-LTPEEL--MDMFQAPETRQFSAGKRQFSAGKRQFSAGKR-------QWVGGE--EEYDPEEMLNMATRQFSAGKR---
Mi_grac      ------------------------------------------QFSAGKRQFSAGKRQFSAGKRQWVGG---------------------------
Op_angu      ----QFSAGKRDWEETELTPEEF--MDMIPLPETRQFSAGKRQFSAGKRQFSAGKR-------QWVGGD--LEYEPEEDLDMETRQFSAGKRQFS
Op_lame      ----QFSAGKRDWEDE-LTPEDL--MDILPAPETRQFSAGKRQFSAGKRQFSAGKR-------QWVGGE----YNPDDMLDMET-----------
Op_impr      ----QFSAGKRDWEE--LTPEDL--SDIVAAPETRQFSAGKRQFSAGKRQFSAGKR-------QWVGGM----ENPDDMLDMETRQFSAGKR---
Ba_hero_a    AGKRQFSAGKRDWEEENLTPQDLLALDMLPLPETRQFSAGKRQFSAGKR--------------QWVGGE--LEYDPNEMLDMETRQFSAGKR---
Ba_hero_b    ----QFSAGKRDWEEENLTPQDLLALDMLPLPETRQFSAGKRQFSAGKR--------------QWVGGE--LEYDPNEMLDMETRQFSAGKR---
Op_vivi      ----QFSAGKRDWEEEELTPEDLLALDMLPVPETRQFSAGKRQFSAGKRQFSAGKR-------QWVGGD--LEYNPEEMLDMETRQFSAGKR---
Op_perf      ----QFSAGKRDWEEDNLTPQDLLALGMLPIPETRQFSAGKRQFSAGKRQFSAGKR-------QWVGGE--QEYDPEDMLDMETRQFSAGKR---
Cl_cana      ----QFSAGKRDWEEEDLTPQDLLALEMLPAPETRQFSAGKRQFSAGKRQFSAGKR-------QWVGGE--TEYNPEDMLDMETRQFSAGKR---
Op_exim_1    ----QFSAGKRDWEEEDLTPQDLLALEMLPLPETRQFSAGKRQFSAGKRQFSAGKR-------QWVGGE--QEYNPEDMLDMETRQFSAGKR---
Op_exim_2    ----QFSAGKRDWEEEDLTPQDLLALEMLPLPETRQFSAGKRQFSAGKRQFSAGKR-------QWVGGE--QEYNPEDMLDMETRQFSAGKR---
Op_liod_a    ----QFSPGKREWDND-LTPEDLLAMGLLPAPETRQFSPGKRQFSPGKRQFSPGKR-------QWVGGE--LEYNPDDMLEMEARQFSPGKR---
Op_liod_b    ----QFSPGKREWDND-LTPEDLLAMGLLPAPETRQFSPGKRQFSPGKRQFSPGKR-------QWVGGE--LEYNPDDMLEMEARQFSPGKR---
As_bidw      ----QFSAGKRDWEQD-LTPEDYLAMEMLPAPETRQFSAGKRQFSAGKRQFSAGKRQFSAGKRQWVGGD----YDPEELLDMETRQFSAGKR---
Op_oedi      ----QFSAGKRDWEQD-LTPEEYLAMEMLPAPETRQFSAGKRQFSAGKRQFSAGKRQFSAGKRQWVGGD----YDPEELLDMETRQFSAGKR---
As_love      -----------DWRQD-LTPEELLAMEMLPAPETRQFSAGKRQFSAGKRQFSAGKRQFSAGKRQWVGGE----YDPEELLNMEARQFSAGKR---
Op_john      ----QFSAGKRDWEEH-LTPEEYLAMEMMPAPETRQFSAGKRQFAAGKRQFSAGKR-------QWIGGQEEQEYNPDDFLDMETRQFSAGKR---
Op_lyma      ----QFSAGKRDWEQN-LNPEEYLAMEMLPAPETRQFSAGKRQFSAGKRQFSAGKR-------QWIGGDEGQEYNPDDFLDMATRQFSAGKR---

Am_cipu      ----QFSAGKRQFSAGKRQWVGGEE--AFLPEMDTRQFSAGKRQFSAGKRQFSAGKRQFSAGKR-------DDGETNILDEILEAEPDLAEA--E
Mi_grac      ---------------------------DVLPDMETRQFSAGKRQFSAGKRQFSAGKR--------------D-SDTHILDEILEAHPDAA-AEGA
Op_angu      AGKRQFSAGKRQFSAGKRQWVGG----DVLPEMETRQFSAGKRQFSAGKRQFSAGKRQFSAGKR-------D-ADTDILDQILNADTTEE----E
Op_lame      -----------------------------------RQFSAGKRQFSAGKRQFSAGKR--------------D--ETNILDEIL--DPAADDALAE
Op_impr      ----QFSAGKRQFSAGKRQWVGGMENPDDMLDMETRQFSAGKRQFSAGKRQFSAGKR--------------D--ETNILDEILEADPAGEDALAE
Ba_hero_a    ----QFSAGKRQFSAGKRQWVGG----DVLPEMDTRQFSAGKRQFSAGKRQFSAGKR--------------D--ETNILDEILEADPAAENALSE
Ba_hero_b    ----QFSAGKRQFSAGKRQWVGG----DVLPEMDTRQFSAGKRQFSAGKRQFSAGKR--------------D--ETNILDEILEADPAAENALSE
Op_vivi      ----QFSAGKRQFSAGKRQWVGG----DALPEMETRQFSAGKRQFSAGKRQFSAGKR--------------D--ETDILDEILQAEPEAEDAFSE
Op_perf      ----QFSAGKRQFSAGKRQWVGG----DVLPEMDTRQFSAGKRQFSAGKRQFSAGKR--------------D--ETNILDEILDAEPAAANALSE
Cl_cana      ----QFSAGKRQFSAGKRQWVGG----DVLPEMDTRQFSAGKRQFSAGKRQFSAGKR--------------D--ETNILDEILEAEPAAANALSE
Op_exim_1    ----QFSAGKRQFSAGKRQWVGG----DVLPEMDTRQFSAGKRQFSAGKRQFSAGKR--------------D--VTNILEEILEAEPAAVDALSE
Op_exim_2    ----QFSAGKRQFSAGKRQWVGG----DVLPEMDTRQFSAGKRQFSAGKRQFSAGKR--------------D--VTNILEEILEAEPAAVDALSE
Op_liod_a    ------------------------------------QFSPGKRQFSPGKRQFSPGKR--------------D--ETNILDEILEAEPAAENALSE
Op_liod_b    ------------------------------------QFSPGKRQFSPGKRQFSPGKR--------------D--ETNILDEILEAEPAAENALSE
As_bidw      ----QFSAGKRQISAGNRQWVGG----EALPEMETRQFSAGKRQFSAGKRQFSAGKR--------------D--ESNILHEILNAEPAAANSLSE
Op_oedi      ----QFSAGKRQFSAGKRQWVGG----EALPEMETRQFSAGKRQFSAGKRQFSAGKR--------------D--ETNILDEILDAEPAAANSLSE
As_love      -----------QFSAGKRQWIGG----EALPDMETRQFSAGKRQFSAGKRQFSAGKR--------------D--ETNILDEILAAEPAVANALSE
Op_john      ------------------QWIGG----DVIPDMETRQFSAGKRQFSAGKRQFSAGKRQFSAGKRQFAAGKRD--DTNILDEFLEANPAENDALSE
Op_lyma      ----QFNPGKRQFSAGKRQWIGG----DAIPNMETRQFSAGKRQFSAGKRQFSAGKR--------------D--ETNILDEILENDPAAENALSE
```

TRH-2

```
Am_cons_2   MLSVACVSPAILL-LSIVSVC---NTVTAEDHALTENENKYLFDRLLLNLRSSANRQKALNDLIRNYIPLDH--SARNKNVLLEVLANLGNVPFD
Am_squa     MFSVTRVSSAILLSAAVLSLW---HTASADDNALTDSENKYLFDRLLLNLRSSENRHKALNDLIRNYIPLDHSTSARNKNVLLEVLANLGDVPFD
Cl_cana     -----CVSSVLFL-LSAVSVW---STVEADDNALTDSENKYLFDRLLHNLRSSENRQKALSDLIRNYIPVDQ--STRNKNILLEVLANLGNVPFD

Am_cons_2   FGGP-ADE-KSATGTADF-WQDD-IDDLEDN--RVNIQL---DDLSKRWGNGPGPRGKRFAMEANSRVIRRDGHGHGPGPRGKRQGPRGKKQGPR
Am_squa     FGGP-ADNTAAATNSEDF-WKDDVADDLEET--RVNIQL---DDLSKRWGSGPGPRGKRFATAVNSRVRRRD----GPGPRGKRQGPRGKKQGPR
Cl_cana     FGGP-AEDPSVNQITQDMQWSADDFNDIESRQNLLNNQL---DDLNKRWGSGPGPRGKRFGVEVNSRVRRRD----GPGPRGKRQGPRGKKQGPR

Am_cons_2   GKKDEEDENEYCLKNPCLQTIARTKYVCDCTEKEDPQKCQAIVED--Q
Am_squa     GKKDEED-----------------------------------------
Cl_cana     GKKEDEE-SEFI--KPCWQTMAKKDYLCDCTNEEDPTNCQIIGGNTHK
```
