# Supplementary material for: Discovery of novel representatives of bilaterian neuropeptide families and reconstruction of neuropeptide precursor evolution in ophiuroid echinoderms
Source: Open Biol. 2017 Sep 6;7(9):170129. doi: 10.1098/rsob.170129 (PMC5627052; doi:10.1098/rsob.170129)
Supplement: Figure S12 [file rsob170129supp12.docx]

>Ophionotus victoriae Neuropeptide-F/Y 1 precursor mRNA partial

AGCTTTGGACGCTATACTATCCGGACAGTACCGATCACATCTTCGCTATGGCAAACGGTTTAATCCAACGCTATTGAAAGATAATAGTATAACCAATTCTGCCGCCATGTCTGGAGGCGTGCATTATGG
